# Effectiveness of a reduced dose of ready-to-use therapeutic food in community-based management of severe acute malnutrition: A non-inferiority randomized controlled trial in the Democratic Republic of Congo

**DOI:** 10.1371/journal.pmed.1004606

**Published:** 2025-05-16

**Authors:** Julien Ntaongo Alendi, Cécile Salpeteur, Steve Botomba, Alemayehu Argaw, Victor Nikièma, Jean-Baptiste Mayavanga, Benjamin Guesdon, Marie Petry, Uwimana Sebinwa, Sophie Bruneau, Aimée Mupuala Masaya, Florence Mbiya Muadi, Samuel Mampunza Ma Miezi, Marie-Claire Muyer

**Affiliations:** 1 University of Kinshasa, Kinshasa, Democratic Republic of the Congo; 2 Research, Analyses & Learning Service, Expertise & Advocacy Dept, Action Against Hunger, Montreuil, France; 3 Department of Nutrition, School of Public Health, University of Kinshasa, Kinshasa, Democratic Republic of the Congo; 4 Department of Food technology, Safety, and Health, Faculty of Bioscience Engineering, Ghent University, Ghent, Belgium; 5 National Nutrition Program, Ministry of Public Health, Kinshasa, Democratic Republic of the Congo; 6 Nutrition & Health Service, Operations Department, Action Against Hunger, Montreuil, France; 7 Department of Pediatrics, University Clinics, University of Kinshasa, Kinshasa, Democratic Republic of the Congo; 8 Neuro-psycho-pathological Center, University of Kinshasa, Kinshasa, Democratic Republic of the Congo; The Hospital for Sick Children, CANADA

## Abstract

**Introduction:**

The reduced Ready-to-use Therapeutic Food (RUTF) dose strategy was demonstrated effective in recovering children with Severe Acute Malnutrition (SAM) in ideal conditions and in a context of food security. The present study was conducted to provide further evidence on the effectiveness of reduced RUTF dose in a context of high food insecurity and post conflict humanitarian crisis, and with routine health staff.

**Methods and findings:**

An individually randomized non-inferiority trial was conducted in 968 children aged 6–59 months suffering from SAM without medical complications in 14 health centers in the Bonzola and Nzaba health zones of Kasaï Oriental, Democratic Republic of Congo (DRC). Children were randomly assigned to either a control group receiving the standard WHO (World Health Organization) RUTF dose at the time or an intervention group receiving a reduced RUTF dose starting from the third week of treatment. The primary outcome was weight gain velocity from admission to discharge from treatment, while secondary outcomes included anthropometry measurements, programmatic outcomes and relapse. Mixed effects linear and logistic regression models with the health center as random intercept were used to compare differences between the two study groups. Close to 94% of the children were from severely food insecure households. There was no difference in weight gain velocity between the two groups (4.88 ± 2.36 g/kg/d reduced dose group versus 5.09 ± 2.28 g/kg/d standard dose group; difference −0.09 g/kg/d (95% CI [−0.33, 0.15]; *p* = 0.46). Programmatic outcomes were also similar between the two groups: recovery rate (64.3% versus 67.0%), loss to follow-up (4.69% versus 5.23%), defaulter rate (2.65% and 1.88%), relapse rate over 3 months (2.86% versus 2.40%) and mean length of stay (42 versus 43 days). Nevertheless, the rate of mid-upper arm circumference (MUAC) gain from the third week onwards was lower in the reduced-dose group than in the standard-dose group, with a mean difference of −0.13 mm/week (95% CI [−0.25, −0.01]; *p* = 0.04). There was no difference in terms of serious adverse events, in the reduced versus standard dose: weight loss (2.24% versus 1.26%), weight stagnation (14.9% versus 17.0%), and medical complications (4.08% versus 3.77%). Important effect modifiers identified were: child sex, child age, season of admission and missed treatment visits.

**Conclusions:**

The strategy of a reduced RUTF dose starting from the third week of treatment is as effective as the standard dose strategy on weight gain velocity and programmatic outcomes in a context of severe food insecurity. However, MUAC gain velocity was lower in the reduced dose group. Future studies should investigate the effectiveness of a reduced dose strategy among sub group of children with high risk factors.

**Trial registration**: International Standard Randomized Controlled Trial Network (ISRCTN15258669).

## Author summary

**Why was this study done?:**

- Ready-to-use therapeutic food (RUTF) is an enriched peanut paste high in calories and nutrients to make up for lost weight and tissue mass, in the management of children suffering from severe acute malnutrition (SAM).
- Many programs report that these children do not achieve the expected weight gain for the amount of RUTF they receive, highlighting that its dosage lacks evidence. Meanwhile WHO also recommended in 2023 the use of a reduced dose of RUTF strategy after a careful evaluation of the child and household situation.
- We aimed at providing robust evidence for effectiveness of a reduced RUTF dosage strategy in a very high food insecurity setting, with a post conflict humanitarian crisis and under a community based SAM management program.

**What did the researchers do and find?:**

- We conducted a non-inferiority individually randomized controlled trial comparing the effectiveness of a reduced RUTF dosage to a standard dosage including a total of 968 children aged 6–59 months suffering from SAM without medical complications, in 14 health centers in Kasaï Oriental, DRC
- The RUTF dosage strategy was based on weight categories, providing standard dose for the first 2 weeks of treatment, protecting the highest weight gain period, then reducing from the third week of treatment until discharge.
- The study results showed that a reduced RUTF dosage is as effective (not inferior) as the standard RUTF dosage in terms of velocity of weight gain, recovery rate, length of stay on treatment, non-response to treatment or relapse within 3 months of recovery. No difference was found in medical complications arising during treatment between the two dose groups. However, children who received a reduced dose had a lower rate of MUAC gain than children who received the standard dose.

**What do these findings mean?:**

- The study results suggest that children with SAM without medical complications can be treated successfully with a reduced dose of RUTF in settings with limited resources or difficulties in the supply of RUTF.
- The limitations of the study lie in the lack of measurement of dietary intake to measure its effect on the community based management of SAM.
- An economic evaluation is also needed to understand the cost benefits of a reduced dose strategy in order to facilitate its adoption at scale in DRC and other humanitarian crisis contexts.

## Abstract 3

In this non-inferiority randomized controlled trial, Julien Ntaongo Alendi and colleagues assess a reduced dose of ready-to-use therapeutic food to treat children with severe acute malnutrition in the Democratic Republic of Congo.

## Introduction

In 2022, more than 13.7 million (2.1%) children under five years old suffered from severe acute malnutrition (SAM) worldwide [1]. In the Democratic Republic of Congo (DRC), one of the worst-affected countries, 7% of children suffered from SAM in 2023 [2]. Difficulties of geographical accessibility and the absence of an efficient supply system make it very difficult to access treatment [3,4].

Faced with aforementioned challenges, field workers in some settings reported to use a reduced quantity of RUTF to treat children with SAM [5]. Although the quantities distributed were reduced, the children treated were recovered [6]. To date, only few scientific studies have evaluated the effectiveness of a reduced dose of RUTF in the management of SAM [7,8]. A 2023 systematic review and meta-analysis commissioned by WHO included three studies with an overall high risk of bias for two of the studies and some concerns for the third study [9]. The review reported the non-inferiority of reduced dose strategy on health outcomes. However, the authors cautioned that the evidence is insufficient and of limited certainty to make firm conclusions.

A cluster randomized trial conducted in rural western Sierra Leone in a post-conflict setting compared standard and reduced RUTF dose strategies in the management of global acute malnutrition in children [10]. The study reported comparability of reduced versus standard RUTF dosage strategies. However, this study has serious limitations in comparability of the study arms including use of different admission and recovery criteria. A non-inferiority Randomized Controlled Trial (RCT), Optima 2 study, was carried out in the Kamwesha health zone, Kasai province in the DRC in children aged between 6 and 59 months suffering from global acute malnutrition, where a small subsample of children with SAM were included [7]. The results of the sub study showed that the reduced dose was not inferior to the standard dose in treatment of SAM in a context of severe food insecurity. In this study, RUTF dosage was determined based on MUAC of children at admission. Another non-inferiority RCT (the MANGO study) evaluated a reduced dose of RUTF starting from the third week of treatment in the outpatient management of children with SAM aged 6–59 months in Burkina-Faso [8]. The study showed that the reduced dose of RUTF was as effective as the standard dose. Nevertheless, a slightly lower velocity of height growth was reported in children who received the reduced dose. This study used RUTF dosage strategy based on children’s weight at admission. However, children with edema were not included in the study as the national protocol instructs such children should receive inpatient care. Furthermore, this study was carried out under ideal conditions, with good household food security status and the engagement of research staff in implementing the study at the health centers, making it difficult to extrapolate the findings for humanitarian crisis contexts.

Generally, there is still not enough evidence to firmly conclude the non-inferiority of reduced RUTF dose strategies in a combination of features such as strictly following WHO admission and discharge criteria, in a real program setting, in a context of high food insecurity and humanitarian crisis situation. The present study was conducted to assess the effectiveness of a reduced dose of RUTF in the management of SAM in children aged 6–59 months on the rate of weight gain (g/kg/d) from admission to discharge. The study was conducted in Kasaï Oriental province, DRC, where household food insecurity and humanitarian crisis are paramount.

## Methodology

### Study design and participants

The EfRAMAS study (which stands for Effectiveness of a Reduced dose of RUTF in the management of SAM, in French) is an individually randomized non-inferiority trial evaluating the effectiveness of a reduced dose of RUTF, as compared to the standard dose treatment. The study was conducted from August 25, 2021 to March 2022, in 14 health centers located in the Bonzola and Nzaba health zones, in Kasaï Oriental, DRC. In 2017, the Grand Kasaï region suffered armed conflict, with over 5,000 deaths, 1.4 million people displaced and more than 600 schools and health centers destroyed [11]. Over 31% of the population of Kasaï Oriental faced severe food insecurity in 2021 [12]. More than 30% of children suffered from acute malnutrition in Bonzola and Nzaba health zones which were reported as a nutritional alert by the DRC’s National Nutrition Program in August 2020 bulletin [13]. There were also high rates of diarrheal diseases (>10%), malaria (>5%), measles (>4%), and acute respiratory illnesses (1%).

The study was carried out as part of a community-based acute malnutrition management program set up by Action Against Hunger (AAH) ensuring drug and RUTF supply, covering health staff costs, technical coaching of health staff and data monitoring. Children of age 6–59 months who were severely acute malnourished, and brought to health facilities, were included in the study. Selection criteria for health centers included: a high patient flow, being geographically accessible and covered by a telephone network. Community health workers (CHW), supported by AAH, were trained and incentivized to identify children with SAM criteria in the community and to refer them for appropriate care at health center. They were also responsible for home visits during study follow up. At presentation, nurses conducted anthropometry assessment, checked for the presence of edema, and conducted appetite test. SAM was defined as having a weight-for-height z-score (WHZ) < −3 SD, and/or MUAC < 115 mm, and/or bilateral edema (+, ++), according to the national protocol [14]. Exclusion criteria included the presence of medical complication, poor appetite, being allergic to RUTF ingredients, admitted for SAM in the last 6 months, having a sibling already included in the study, or presenting with a physical deformity preventing anthropometry measurement. The nurse explained the research procedure to the family caregiver and requested their written consent for participation. Then study children were randomly allocated to either the standard or the reduced dosage group by assigning the next unique identifier number in the randomization list in the order of their arrival. The study is reported according to the CONSORT 2010 extension for reporting randomized non-inferiority and equivalence trials [15].

### Randomization and masking

Children were individually randomized to the standard or reduced dose groups. Block randomization using 14 health centers as blocks, was applied. Randomization lists were generated from a list of the 14 health centers and the number of children expected per health centers between 31 and 97, using www.randomization.com by an independent researcher not involved in the study. The randomization lists were printed and then sent directly to the nurses in the health centers. On admission, each child was given a unique identifier corresponding to his or her study group. The nurses and supervisors were not blinded of allocation of children to study arms.

### Ethics

In carrying out this study, the principles of the Declaration of Helsinki were strictly respected. The EfRAMAS study protocol was approved by the Ethics Committee of the School of Public Health of the University of Kinshasa (ESP/CE/127/2021). Caregivers were asked to give their written informed consent before inclusion of their child into the study. They were informed that they were free to participate and that they could withdraw at any time without giving any particular explanation. All children whose family caregivers did not agree to participate in the study or did not meet the study inclusion criteria still received treatment for SAM according to the national protocol in DRC. Confidentiality of participants’ information was guaranteed and electronic data was password protected. The very tight work schedule of the research team requiring staying in the field with restricted access to internet during the preparation of the study was a challenge to register our study before the start of children enrolment. As a result, the study protocol was registered at the International Standard Randomized Controlled Trial Network (ISRCTN15258669) on September 11, 2021, i.e., two weeks after the start of enrolment.

### Procedures

In both groups, each child was monitored weekly from admission to discharge from treatment, for a maximum of 12 weeks. All children, regardless of group, received the same amount of RUTF during the first two weeks (Table 1). Starting from the third week of treatment, the amount of RUTF in the reduced dose group was reduced by 22% to 50% depending on the child’s weight. In the standard dose group, children continued to receive the same amount of RUTF until the end of treatment. RUTF was provided in sachets containing 92 g of the enriched peanut paste providing 500 kcal each (Nutriset SA, France). All children admitted to the study, regardless of group, received systematic medical treatment according to national protocol [14].

**Table 1:** Quantity of RUTF distributed according to child’s weight and study group.

Weight (kg)	Sachets/weeks			Percent of reduction RUTF in reduced group from week 3
	Standard dose	Reduced dose		
	Admission to discharge	Weeks 1–2	Week 3 to discharge	
3.0–3.4	9	9	7	22.2
3.5–4.9	11	11	7	36.4
5.0–6.9	14	14	7	50.0
7.0–9.9	21	21	14	33.3
10.0–14.9	28	28	14	50.0

RUTF: ready-to-use therapeutic foods. kg: kilogram.

The children received medical treatment according to the symptoms presented. Caregivers received a health and nutritional consultation by the nurses during the weekly visit, and are reminded of the importance of giving each child the prescribed dose of RUTF. For each child who missed a weekly visit, the CHW were incentivized to make a home visit to check if the child was well and the reason for the absence. Children who developed complications during treatment were referred to the inpatient treatment and then reintegrated into their respective study group once the complication was managed. For children declared recovered, follow-up was carried out once every 14 days for 3 months, as stipulated in the national protocol [14]. No nutritional treatment was administered during this fortnightly follow-up due to the absence of a supplementary feeding program.

Nurses, CHW and data entry agents were trained over 3 days and took part in a pre-test exercise. At admission, information on socio-demographic and household characteristics was gathered from caregivers. Household Food Insecurity Access Scale (HFIAS) was applied to assess the food security status of households [16]. The scale scores household situation from 0 (good food security) to 27 (severe food insecurity). Based on this score, households were classified as food secure, mildly food insecure, moderately food insecure or severely food insecure. Anthropometrics, nutritional edema and clinical status data were collected weekly during treatment. MUAC was measured using a MUAC tape to the nearest 1 mm, weight to the nearest 100 g using an electronic scale (SECA 876, SECA, Hamburg, Germany), height using a wooden measuring board (UNICEF) to the nearest 1 mm, and bilateral nutritional edema were assessed according to severity (none (0); mild (+); and moderate (++)). Data entry agents extracted data from patient records using tablets on site with the Kobo Collect application (Kobo Collect v2022.4.4).

### Outcomes

The primary study outcome was the weight gain velocity (g/kg/day) between admission and discharge. It is calculated by dividing the total weight gained during treatment (weight at discharge − weight at admission) in grams, divided by the weight at admission in kg and the length of stay in days. We also calculated the weight gain velocity starting from third week of treatment, when dose reduction occurred (weight at discharge − weight at third week) in grams divided by weight at admission in kg and length of stay from third week to discharge (in days). Weight gain velocity was chosen as it is a continuous outcome, which enables detecting even small differences between the reduced and standard dose groups instead of a programmatic outcome such as recovery based on attained weight for height and MUAC cut-off values.

Secondary study outcomes included length of stay (in days) defined as time elapsed between admission and discharge from treatment, regardless of the mode of discharge (i.e., recovery, default, death, non-response, or false discharge). Recovery is considered when a child is presented with a WHZ ≥ −1.5 and MUAC ≥ 115 mm and no edema for two consecutive visits. Default was considered when a child was absent for more than two consecutive weeks and confirmed alive by CHW follow-up visit. Lost to follow-up happened when a child was absent as declared by nurse, for more than two consecutive weeks but the child could not be traced. Death was recorded when a child died at any time during treatment. Non-response was defined when a child was not recovered until the twelfth week of treatment. False discharge applied to children who were discharged as recovered by the nurse, yet were found to be not recovered at the analysis stage. Duration of edema melting (in days) was the time needed for the edema to completely disappear. Relapse was considered when a recovered child was found to have SAM after discharge at any time over 3 months. MUAC gain velocity was calculated using the gain in mm per week in MUAC divided by the length of stay from admission to discharge of the child, and again for the period starting from third week of treatment. Medical complications are defined by the occurrence of an episode of fever for ≥3 days, and/or diarrhea (having ≥3 loose stools/day) for ≥3 days, and/or cough for ≥3 days and/or vomiting for ≥3 days and/or reoccurrence of edema, according to caregiver report and nurse’s assessment. A stagnant weight was identified as <0.1 kg weight gain between two consecutive visits. Loss of weight was identified as ≥5% weight loss between two consecutive visits. Any serious adverse event was considered in the presence of medical complications, stagnant weight, loss of weight and/or reoccurrence of edema.

### Data management and statistical analyses

In this study, the expected mean (SD) rate of weight gain was 5 g/kg/d (2.6) and the expected difference between the study groups was 0 g/kg/d with a non-inferiority margin of 0.5 g/kg/d. Assuming a power of 80%, a significance level of 5%, and expected attrition rate of 20%, the required sample size was 804 (402 in each group). Following the use of WHO unisex WHZ table at health centers, some girls with moderate acute malnutrition were misclassified as having SAM. For this reason, we further inflated the total sample size to 1,200 children.

Data were extracted from Kobo in coma separated value format, and data cleaning and analyses were conducted using Stata 17 (StataCorp, LLC, TX, USA). Implausible and missing values were verified with the original patient record. Missing height values were replaced by height measurement from previous visit. Missing weight and MUAC values were replaced by the average of the previous and next visits. Anthropometric indices of WHZ, WAZ and height-for-age Z-score (HAZ) were calculated using WHO 2006 child growth standards [16]. Extreme values of anthropometric indices were discarded from analyses using cut off values for WHZ: −5 to +5; for WAZ: −6 to +5; for HAZ: −6 to +6.

The statistical analyses plan for this study was published on the 15/05/2023 on the United Kingdom (UK) Clinical Registry (https://www.isrctn.com/ISRCTN15258669). Descriptive statistics were presented using percentage, median or mean depending on the type of study variables. Outcomes variables were assessed for normality using histograms and Q–Q plot of the outcomes and model residual terms. Continuous outcome variables were compared between the two study groups using mixed-effects linear regression models with health center as a random intercept. Mixed-effects logistic regression models with health center as a random intercept were used for binary outcomes. The evolution of recovery rates between study groups was compared using the Cox proportional hazards model, and the resulting survival curve was presented using a Kaplan–Meier curve. As a sensitivity analysis, we applied two definitions of recovery rate: (i) based on 2 consecutive consultations as per standard protocol, and (ii) based on only one consultation as per routine programmatic practice.

Health center, child weight, height, sex and age, month of admission, and edema were used as adjustment factors for the models. All subjects randomized to the study, regardless of treatment outcome, were included in the intention-to-treat (ITT) analysis. In per protocol (PP) analyses, children were included if they received the correct dose of RUTF and completed the treatment, as recovered, deceased or non-responder (i.e., excluding confirmed defaulters, lost to follow-up, mistakenly declared recovered by the program and consent withdrawals). Children were considered not taking the correct dose if they were given incorrect dose for more than 2 weeks according to their study arm and weight category. Furthermore, we conducted pre-planned subgroup analyses on rate of weight gain and other secondary outcomes (recovery rate, defaulting rate, non-response to treatment, false discharge, loss to follow up, relapse over 3 months, serious adverse events, medical complications during treatment, weight gain velocity starting from third week). Pre-specified subgroup factors included child sex, age (<12 versus ≥12 months), admission criteria (MUAC-only, WHZ-only, edema-only, MUAC and edema, WHZ and MUAC), occurrence of edema at any visit, season of admission, morbidity (yes or no), missed visits (yes or no) and household food security status. Subgroup analyses were conducted in the presence of significant interaction (*p* < 0.1) between the study arms and the aforementioned subgroup variables.

### Role of funding source

The funders had no role in study design, data collection and analysis, decision to publish, or preparation of the manuscript.

## Results

### Participants’ characteristics

**Fig 1:**
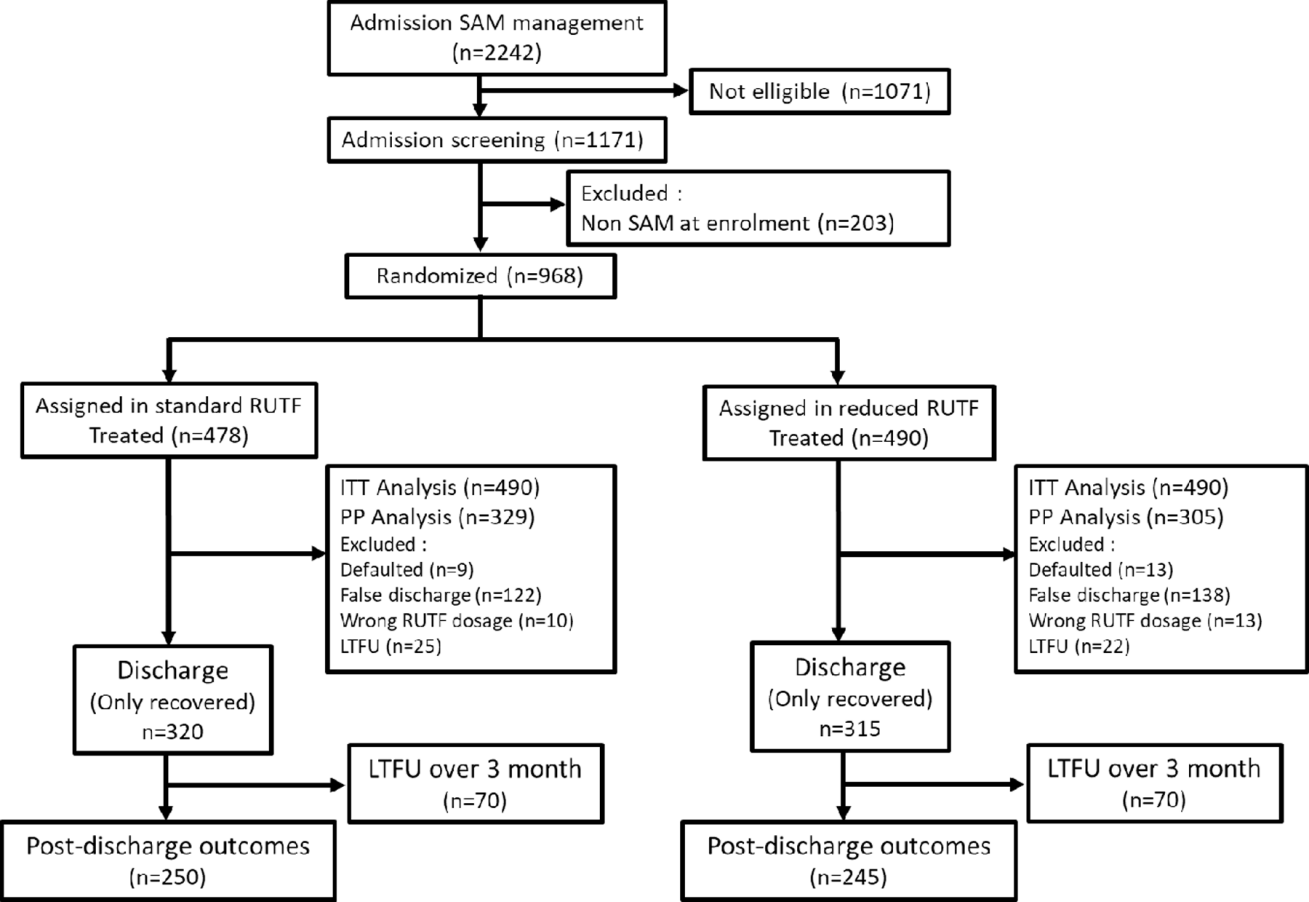
Patient flowchart. SAM: severe acute malnutrition; RUTF: read-to-use therapeutic food; ITT: intention to treat; PP: per protocol; LTFU: lost-to-follow up.

Between 25th of August and 19th of November 2021, 2,242 children were admitted to the 14 health centers, of whom 1,171 were recruited. However, because of the use of WHO unisex table, resulting in misclassification of some children (*n* = 203) with moderate acute malnutrition as having SAM, only 968 children (478 in the standard and 490 in the reduced dose group) were included in the analyses (Fig 1) [17]. The household and sociodemographic characteristics of participants at admission were comparable between the study groups (Table 2). The mean (SD) age was 29.5 (13.8) months, with only 11% being <12 months, 54.2% male and 2.89% with a low birth weight according to caregiver report. The mean (SD) weight was 7.97 (1.69) kg, mean height was 78.3 (9.36) cm and mean MUAC was 113 (5.70) mm. Regarding admission criteria, 44.3% of children were admitted on the basis of MUAC alone, 22.7% on the basis of WHZ alone, 6.40% with nutritional edema, 26.1% on the basis of MUAC and WHZ, and 0.41% on the basis of edema and MUAC. In total, 93.6% of the children lived in a situation of severe household food insecurity and 69.6% were vaccinated against measles.

**Table 2:** Baseline characteristics of 968 enrolled children with SAM^a^.

Characteristics	*n*	Reduced RUTF	Standard RUTF
Age, months	968	29.1 ± 13.5	30.0 ± 14.1
Age categories	968		
<12 months, *n* (%)		49/490 (10.0)	47/478 (9.59)
≥12 months, *n* (%)		441/490 (90.0)	431/478 (88.0)
Male, *n* (%)	968	268/490 (54.7)	257/478 (53.8)
Weight, kg	968	7.98 ± 1.70	7.96 ± 1.69
Height, cm	968	78.3 ± 9.25	78.4 ± 9.48
MUAC, mm	968	114 ± 5.91	113 ± 5.47
WHZ	901	−2.95 ± 0.87	−2.96 ± 0.84
HAZ	968	−3.23 ± 1.73	−3.30 ± 1.68
WAZ	902	−3.79 ± 0.92	−3.82 ± 0.92
Stunted (HAZ < −2)	968	370/490 (75.5)	365/478 (76.3)
Admission criteria	968		
MUAC-only		212/490 (43.3)	217/478 (44.3)
WHZ-only		121/490 (24.7)	99/478 (20.2)
Edema-only		36/490 (7.35)	26/478 (5.31)
WHZ and MUAC		118/490 (24.1)	135/478 (27.6)
MUAC-edema		3/490 (0.61)	1/478 (0.20)
Low birth weight	968	13/490 (2.65)	13/478 (3.14)
Urban	968	260/490 (53.1)	260/478 (54.0)
Health center = <30 min return trip	968	284/490 (58.0)	284/478 (61.3)
Caregiver’s age (years)	944	28.7 ± 6.58	29.1 ± 6.69
Mother has no formal education	968	34/490 (6.94)	34/478 (8.16)
HFIAS category	968		
Food-secure		10/490 (2.04)	6/478 (1.26)
Mild food-insecure		3/490 (0.61)	8/478 (1.67)
Moderate food-insecure		15/490 (3.06)	20/478 (4.18)
Severe food-insecure		462/490 (94.3)	444/478 (92.9)
Open defecation	968	69/490 (14.1)	69/478 (14.9)
Measles vaccine (card confirmation)	968	343/490 (70.0)	331/478 (69.2)
Breastfed	968	339/490 (69.2)	339/478 (70.9)

^a^*Values presented as mean* ± *standard deviation or *n* (%). SAM: MUAC < 115 mm, and/or WHZ score < –3 and/or edema. HAZ: height-for-age Z. MUAC: mid-upper-arm circumference. RUTF: ready-to-use therapeutic food. WAZ: weight-for-age Z. WHZ: weight for-height Z. HFIAS: household food insecurity access scale.*

### Primary outcome

**Fig 2:**
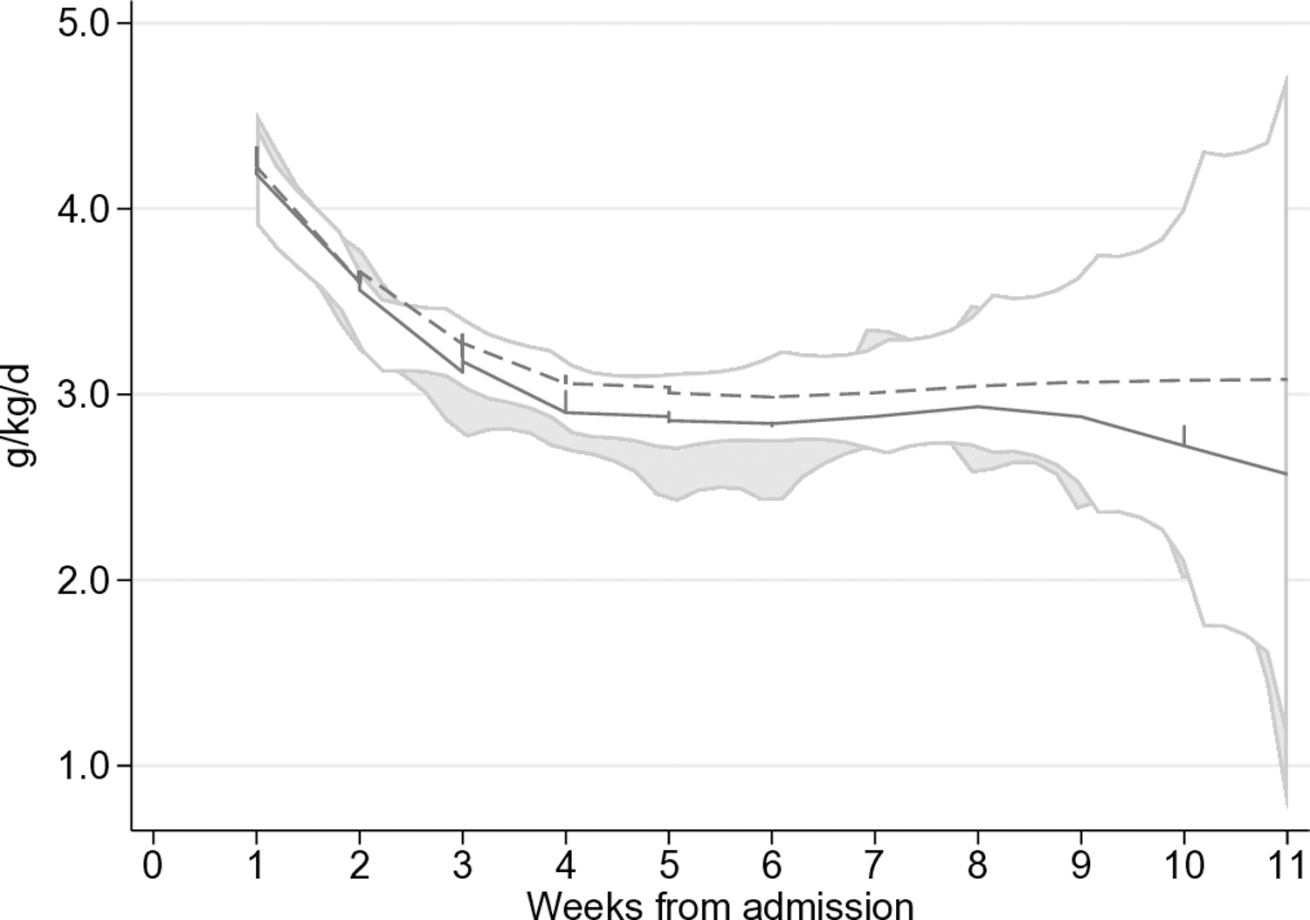
Weekly weight gain velocity (g/kg/day) of children with SAM randomized to reduced or standard RUTF dose, modelled using the mean weight of each group per visit. g/kg/d: grams per kilogram per day. Solid line: reduced dose. Dash line: standard dose.

**Fig 3:**
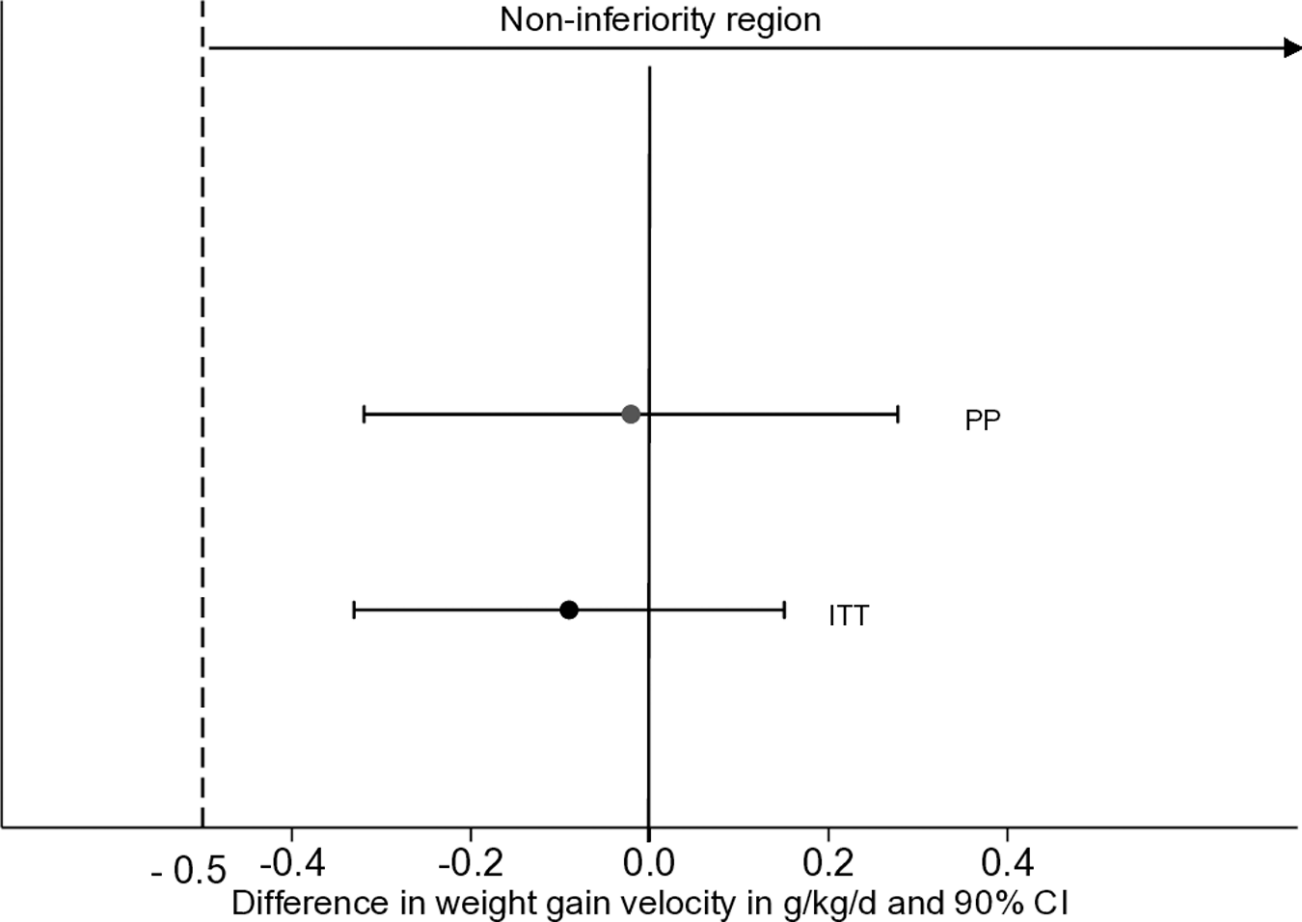
Difference in mean weight gain velocity (g/kg/day and 90% CI) in children with SAM randomized to reduced dose compared with standard dose in ITT and PP confirming non-inferiority. ITT, intention to treat; PP, per protocol; SAM, severe acute malnutrition; g/kg/d: grams per kilogram per day.

In ITT analysis, the mean (SD) weight gain velocity (g/kg/d) from admission to discharge was similar between the reduced dose (4.88 ± 2.36) and the standard dose (5.09 ± 2.28) groups with a difference of −0.09 g/kg/d (95% CI [−0.33, 0.15]; *p* = 0.46) (Table 3). The weight gain velocity was greatest in the first two weeks, then declined rapidly (Fig 2). Although the mean rate of weight gain decreased from the third week of treatment (4.26 ± 2.13 g/kg/d for the reduced dose versus 4.52 ± 2.22 g/kg/d for the standard dose), there was still no difference in ITT analyses −0.15 g/kg/d (95% CI [−0.38, 0.09]; *p* = 0.22). Non-inferiority of weight-gain velocity in the reduced dose versus the standard dose was confirmed in ITT (inferiority rejected, *p* = 0.46) and in PP analyses (inferiority rejected, *p* = 0.89) (Fig 3). No difference −0.00 g/kg/d (95% CI [−0.25, 0.25]; *p* > 0.999) in weight gain velocity was observed for children declared recovered or defaulters −0.59 g/kg/d (95% CI [−2.72, 1.54]; *p* = 0.59). Results were similar for PP analyses.

**Table 3:** Weight and MUAC gain velocities^a^ of children with SAM randomized to reduced or standard RUTF dose.

Outcome	Reduced RUTF		Standard RUTF		Unadjusted model		Adjusted model	
					Difference (95% CI)	*p*-value	Difference (95% CI)	*p*-value
***Admission to discharge***								
**Weight gain velocity (g/kg/d)**								
ITT	490	4.88 ± 2.36	478	5.09 ± 2.28	−0.21 (−0.46; 0.05)	0,11	−0.09 (−0.33; 0.15)	0.46
PP	302	4.79 ± 2.36	329	5.04 ± 2.35	−0.26 (−0.58; 0.06)	0.11	−0.02 (−0.32; 0.28)	0.89
Recovered	315	5.34 ± 2.21	320	5.56 ± 2.10	−0.23 (−0.52; 0.06)	0.12	−0.00 (−0.25; 0.25)	1.00
Defaulted	13	2.70 ± 2.29	9	2.81 ± 2.28	−0.10 (−1.96; 1.75)	0.91	−0.59 (−2.72; 1.54)	0.59
**MUAC gain velocity (mm/w)**								
ITT	490	2.17 ± 1.00	478	2.32 ± 0.95	−0.15 (−0.26; −0.04)	0.01	−0.08 (−0.18; 0.03)	0.16
PP	302	2.13 ± 1.04	329	2.31 ± 0.92	−0.17 (−0.30; −0.04)	0.01	−0.08 (−0.21; 0.05)	0.21
***After two weeks*** ^*^								
**Weight gain velocity (g/kg/d)**								
ITT	455	4.26 ± 2.13	440	4.52 ± 2.22	−0.24 (−0.48; 0.01)	0.06	−0.15 (−0.38; 0.09)	0.22
PP	273	4.24 ± 1.97	298	4.55 ± 2.22	−0.32 (−0.60; −0.03)	0.03	−0.14 (−0.41; 0.14)	0.32
**MUAC gain velocity (mm/w)**								
ITT	455	2.11 ± 1.09	440	2.31 ± 1.01	−0.18 (−0.30; −0.06)	0.00	−0.13 (−0.25; −0.01)	0.04
PP	273	2.14 ± 1.13	298	2.36 ± 0.96	−0.20 (−0.35; −0.0)	0.01	−0.12 (−0.28; 0.04)	0.13

^a^*Values presented as *n* or mean* ± *standard deviation.*

* *Before the third week, 45 children (5%) were already discharged. ITT: intention to treat. MUAC: mid-upper arm circumference. PP: per protocol. RUTF: ready-to-use therapeutic food. SAM: severe acute malnutrition. mm/w: millimeter per week. g/kg/d: grams of weight gain per body weight per day.*

On subgroup analyses, there was a significant interaction (*p*-interaction = 0.001) between study group allocation and age of children (<12 months versus ≥12 months) (Table B in S1 Text). However, group differences remain non-significant within both age categories. Similarly, we found significant interaction (*p*-interaction = 0.079) for missing visits (being absent for at least one week consultation versus no absence), whereas group differences within each subgroup remained non-significant.

### Secondary outcomes

**Fig 4:**
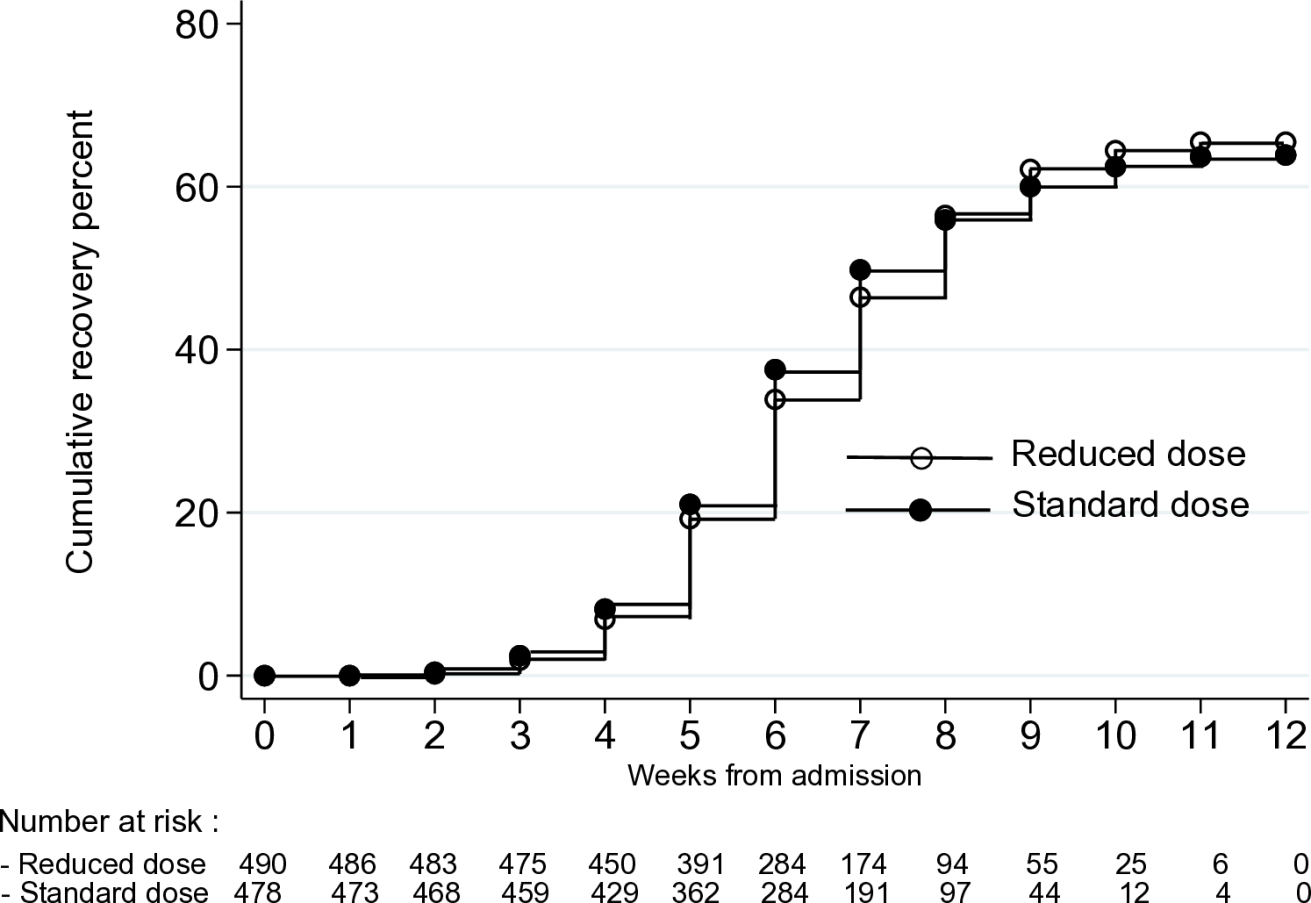
Weekly recovery proportion among children with SAM randomized to reduced or standard RUTF dose.

Recovery rate was similar for the reduced (64.3%) and standard dose (67.0%) groups with a difference of −1.53 (95% CI [−7.64, 4.58]; *p* = 0.62) (Table 4). Similarly, time to recovery using the Cox proportional hazard model showed no difference between the two study groups (Hazard Ratio: 1.04; (95% CI [0.88, 1.23]; *p* = 0.63) (Fig 4). No significant interaction was observed between study groups and the subgroup factors mentioned in the statistical analysis on recovery rate (Table E in S1 Text). Sensitivity analyses for recovery rate using the programmatic criteria of one visit was not significantly different between the two study groups (*p* = 0.387). The proportion of defaulters in the reduced versus standard dose groups (2.65% versus 1.88%; *p* = 0.67), lost to follow-up children (4.69% versus 5.23%; *p* = 0.19), false discharge children (28.2% versus 25.5%; *p* = 0.57) and non-responders (0.41% versus 0.21%) were also similar between the two study groups. We found similar results with PP analyses (Table A in S1 Text).

**Table 4:** Programmatic outcomes^*^ in the reduced and standard dose groups of children using ITT analyses.

Outcome	Reduced RUTF		Standard RUTF		Unadjusted difference (95% CI)	*p*-value	Adjusted difference (95% CI)	*p*-value
Recovery	490	315/490 (64.3)	478	320/478 (67.0)	−2.79 (−8.56; 2.98)	0.34	−1.53 (−7.64; 4.58)	0.62
Default	490	13/490 (2.65)	478	9/478 (1.88)	0.85 (−1.27; 2.97)	0.41	0.34 (−1.55; 2.23)	0.24
Died	490	0	478	1/478 (0.21)				
False discharge	490	138/490 (28.2)	478	122/478 (25.5)	2.80 (−2.68; 8.27)	0.32	1.64 (−4.01; 7.29)	0.57
Non responders	490	2/490 (0.41)	478	1/478 (0.21)				
Lost-to-follow up	968	6/490 (0.62)	478	25/478 (5.23)	−0.64 (−3.58; 2.31)	0.67	−0.66 (−3.67; 2.36)	0.19
Edema melting	39	14 [7–15]	26	7 [7–14]	1.63 (−1.82; 5.07)	0.35	1.96 (−1.51; 5.43)	0.27
Serious undesirable effects								
Weight loss	490	11/490 (2.24)	478	6/478 (1.26)	1.12 (−0.83; 3.07)	0.24	0.18 (−1.24; 1.60)	0.83
Stagnant weight	490	73/490 (14.9)	478	81/478 (17.0)	−1.88 (−6.39; 2.64)	0.41	−2.64 (−7.31; 2.03)	0.26
Medical complication	490	20/490 (4.08)	478	18/478 (3.77)	0.32 (−2.09; 2.73)	0.79	−0.02 (−2.40; 2.36)	0.99
Length of stay, days	490	42 [35–51]	478	43/478 [35–56]	−0.31 (−2.04; 1.41)	0.72	0.03 (−1.62; 1.68)	0.97
Relapse	245	7/490 (2.86)	250	6/478 (2.40)	0.46 (−2.36; 3.28)	0.75	0.27 (−1.56; 2.10)	0.10

* Values are presented as mean ± standard deviation, or *n* (%), or IQR [min, max]. During post-treatment follow-up, 140 children were lost to follow-up, 70 in each group. RUTF: ready-to-use therapeutic food. CI: confidence interval.

MUAC gain velocity (mm/week) from admission to treatment discharge was similar between the two study groups with a difference of −0.08 (95% CI [−0.18, 0.03]; *p* = 0.16) (Table 4). However, MUAC gain velocity after two weeks was significantly lower in the reduced dose group as compared to the standard dose group in ITT analyses with a difference of −0.13 (95% CI [−0.25, −0.01]; *p* = 0.04), but not statistically significant difference was found in PP analyses −0.12 (95% CI [−0.28, 0.04]; *p* = 0.13). Subgroup analyses revealed the presence of interaction between study arms and season of admission (*p*-interaction = 0.015) and missed visits (*p*-interaction = 0.068). MUAC gain velocity was inferior among the reduced dose group in children who were admitted during dry season with a difference of −0.24 (95% CI [−0.42, −0.05]; *p* = 0.011). Similarly, the reduced dose group had a lower MUAC gain velocity than the standard dose among children who attended all treatment visits −0.28 (95% CI [−0.54, −0.02]; *p* = 0.035).

Length of stay (in days) was similar between the two study groups (median (IQR) reduced versus standard dose: 42 (35−51) versus 43 (35−56); with a difference of 0.0 (95% CI [−1.6, 1.7]; *p* = 0.97)). Difference between reduced and standard dose groups on length of stay was modified by sub group factors such as child sex (*p*-interaction = 0.050), and season of admission (*p*-interaction = 0.081). Duration of edema melting (days) was higher among reduced dose group although not statistically significant (median (IQR) reduced versus standard dose: 14 (7−15) versus 7 (7−14); difference of 2.0 (95% CI [−1.5, 5.4]; *p* = 0.27)).

The proportion of children with serious adverse events was 18.8% including weight loss (2.24% versus 1.26%; *p* = 0.83), weight stagnation (14.9% versus 16.9%; *p* = 0.26) and medical complications (18.4% versus 19.0%; *p* = 0.56) (Table 4). There was no case of reoccurrence of edema. During treatment, only one child died in the standard-dose group. This was a child with multidrug-resistant tuberculosis, who was immediately admitted and transferred to a tuberculosis treatment center, where he died.

There was no difference in terms of anthropometric measurements at treatment discharge for all children declared recovered between study groups (Table 5). In total, the rates of stunting (HAZ < −2), underweight (WAZ < −2), wasting using WHZ < −2, and wasting using MUAC < 125 mm were 77.5%, 80.6%, 14.8%, and 10.1%, respectively.

**Table 5:** Anthropometry measurements^*^ of children at discharge by study groups.

Outcome	*n*	Reduced RUTF	Standard RUTF	Unadjusted model		Adjusted model	
				Difference (95% CI)	*p*-value	Difference (95% CI)	*p*-value
Weight, kg	635	9.83 ± 1.82	9.77 ± 1.81	0.09 (−0.18; 0.35)	0.53	−0.04 (−0.11; 0.03)	0.26
Height, cm	635	79.39 ± 9.15	78.87 ± 8.72	0.57 (−0.80; 1.94)	0.42	−0.02 (−0.11; 0.07)	0.67
MUAC, mm	635	128.65 ± 3.25	128.63 ± 3.14	0.08 (−0.37; 0.54)	0.72	−0.06 (−0.41; 0.29)	0.72
WHZ	635	−1.20 ± 0.75	−1.26 ± 0.72	0.06 (−0.05; 0.17)	0.26	−0.01 (−0.08; 0.06)	0.75
HAZ	612	−3.13 ± 1.55	−3.26 ± 1.51	0.15 (−0.09; 0.38)	0.23	0.06 (−0.06; 0.17)	0.32
WAZ	635	−2.67 ± 0.98	−2.78 ± 0.97	0.13 (−0.02; 0.27)	0.08	0.02 (−0.06; 0.10)	0.62

* Values are presented as mean ± standard deviation. RUTF: ready-to-use therapeutic food. HAZ: height-for-age Z. MUAC: mid-upper-arm circumference. WAZ: weight-for-age Z. WHZ: weight for-height Z.

## Discussion

The results of this RCT demonstrated that the reduced dose strategy is non-inferior to the standard dose in terms of velocity of weight gain, even in a context of severe food insecurity. Furthermore, the study showed that the reduced dose is effective at rehabilitating children with SAM in a community-based management of SAM setting. The effectiveness of the reduced dose was also similar to that of the standard dose in terms of various programmatic outcomes such as recovery, default, non-response, relapse rates and length of stay in treatment. However, children in the reduced dose group had a lower velocity of MUAC gain in ITT analyses. Furthermore, the duration of edema melting was longer in the reduced dose group although this was not statistically significant. Finally, anthropometric status of children in the two groups were similar at the end of treatment.

The velocity of weight gain was maximal in the first two weeks, then decreased rapidly from the third week onwards, as seen in other studies [8,18]. Weight gain velocity in this study was close to 5 g/kg/d, in line with WHO recommendations [19], and higher than observed in the majority of SAM management programs [6,20,21] and in RCTs conducted in Burkina Faso, the DRC and Malawi [8,22]. On the other hand, the velocity of weight gain seems to be lower in the reduced dose group starting from week 5 of treatment (Fig 2), although this was not reflected in the group comparison for the whole period of treatment (Table 3). These observed differences towards the end of the treatment visits may suggest that the reduced dose may not be as effective as the standard dose in a small sub group of children with relatively delayed recovery. These findings support WHO guideline that cautions the use of a reduced dose after a careful assessment of the specific child’s and its household situation is done [19].

WHO recommends an energy requirement of 155–185 kcal/kg/d for children with SAM to achieve a weight gain of 5–10 g/kg/d [19]. On the other hand, Sachdev and colleagues [23] argue that an energy intake of 92–110 kcal/kg/d would be enough to attain a weight gain velocity of 5 g/kg/d while avoiding calorie overfeeding. This could explain why children in the reduced dose group showed similar recovery rates to children in the standard dose group in our study. However, the decreased amount of essential nutrients intake in the reduced dose group can have an impact on the composition of weight gain (lean and fat masses) [24]. Unfortunately, body composition was not assessed in the current study. The MANGO trial in Burkina Faso reported a reduced dose is not inferior in lean body mass accretion compared to standard dose. However, the latter study was conducted in a different setting where household food insecurity was not high.

The recovery rate in the present study was 65.5% when applying the most stringent WHO criteria and was 92% when applying less stringent routine practice criteria (using a unisex WHZ chart and only one visit to qualify for recovery). According to the SPHERE standards, the expected recovery rate is at least 75% [25]. The rates of mortality (0.2%), defaulter (3.3%), and non-response to treatment (0.45%) meet the 2019 SPHERE standards. The length of stay in the current study (6 weeks) is on the higher margin of SPHERE standards of 4–6 weeks. The rate of relapse (2.63%) to SAM over 3 months after treatment was similar to the one found in the MANGO study (2.1%) in Burkina Faso [8], but lower than that found in a study in Nepal (33%) [26]. Furthermore, our study cohort remains with high rates of stunting (77.5%), underweight (80.6%) and wasting (14.8%) when discharged from treatment. These results indicate the need for post treatment nutritional and health interventions in this setting.

MUAC gain velocity after two weeks was lower for children who received the reduced dose compared with the standard dose, contrary to what was found in other studies including MANGO [8] and Optima 2 [7,8]. Furthermore, the duration of edema melting was longer in the reduced dose group although this was not statistically significant. Our subgroup analyses indicate important effect modifiers on the comparability of the reduced and standard dose groups such as child sex, child age, admission criteria, missed treatment consultations, and season of admission. However, our analyses comparing the two study groups within each effect modifier factor using small sample sizes did not find statistically significant differences. Furthermore, these analyses did not take into account potential false discoveries due to multiple testing. As a result, future studies with adequate sample size are required to further investigate the effectiveness of the reduced dose among high-risk children characterized by a combination of the above mentioned factors.

The average amount of RUTF sachets prescribed to children was 126 in standard dose and 108 in reduced dose groups, resulting in 14.3% (18/126) savings on RUTF purchase costs. By reducing the quantity of RUTF distributed to children suffering from SAM, it will be possible to treat more children with the same resources compared with the standard dose. This strategy allows indirectly increasing the coverage of SAM treatment programs, by reinvesting savings made per treatment case in new admissions of children suffering from SAM, as recommended by WHO. This is especially critical in a country like the DRC where the government, through the PRONANUT (National Program of Nutrition), is facing barriers to increase the coverage of services for children with SAM.

The current study has strengths and limitations requiring attention. In the current study context, household food insecurity was highly prevalent suggesting the generalizability of our findings to other similar settings. This was further enhanced by testing the reduced dose under a program setting. On the other hand, children with edematous SAM were relatively underrepresented in our study. Furthermore, the performance of the reduced dose seems to be inferior in this subgroup of children as shown by the duration for disappearance of edema. Therefore, the findings among subgroup of children with edema should be interpreted carefully. We had relatively higher rate of lost to follow-up cases compared to other similar studies [8,22], which can be explained by the fact that, in this study, follow-up was carried out as per the standard protocol, whereas in the other studies cited, follow-up was closer. Caregivers and nurses were not blinded. However, this seems to have a limited impact on the objective anthropometry measurements. We did not directly measure RUTF sharing, which might have had a different effect on the two study groups. Regardless of this, we did not find a significant difference between the study groups on the primary outcome velocity of weight gain. Finally, measurement of additional parameters would have enhanced the interpretation of our findings. These include a dietary intake study to understand the role of family foods among children with SAM managed in outpatient care, and body composition assessment to see the comparability of the reduced and the standard dose in terms of fat and fat-free mass accretion.

This RCT confirms the effectiveness of a reduced dose of RUTF during management of children with uncomplicated SAM in a context of food insecurity in terms of weight gain velocity and programmatic outcomes. Relapse rate was also similar between the two dose strategies. However, the reduced dose seems to be inferior on MUAC gain velocity when the dose reduction occurs. Futures studies should investigate the effectiveness of a reduced RUTF dose conditioned at reaching a MAM status according to the new WHO guideline. These studies also need to investigate the reduced dose strategy using adequate sample of high-risk children characterized by a combination of effect modifiers.

## Supporting information

S1 EfRAMAS protocol Overall study protocol. (PDF)

S1 EfRAMAS statistical plan Statistical analysis plan of the study. (PDF)

S1 CONSORT checklist (DOC)

S1 Text Results of sub-group analyses. (DOCX)

## Abbreviations

AAH: action against hunger
HAZ: height-for-age z-score
HFIAS: Household Food Insecurity Access Scale
ITT: intention-to-treat
LTFU: lost-to-follow up
MUAC: mid-upper arm circumference
PP: per protocol
RCT: randomized controlled trial
RUTF: ready-to-use therapeutic food
SAM: severe acute malnutrition
WHZ,: Weight-for height z-score;
WAZ,: weight-for age z-score.
