# Supplementary material for: Effectiveness of a reduced dose of ready-to-use therapeutic food in community-based management of severe acute malnutrition: A non-inferiority randomized controlled trial in the Democratic Republic of Congo
Source: PLoS Med. 2025 May 16;22(5):e1004606. doi: 10.1371/journal.pmed.1004606 (PMC12084047; doi:10.1371/journal.pmed.1004606)
Supplement: S1 EfRAMAS protocol — (PDF) [file pmed.1004606.s001.pdf]

<u>Research protocol</u>

EfRAMAS project

Efficacy of a reduced dose of ready-to-use therapeutic food (RUTF) in children under 5 with uncomplicated severe acute malnutrition (SAM) compared to a standard dose: a controlled, randomized trial of no -inferiority without additional staff in the health zones of Bonzola and Nzaba, city of Mbuji-Mayi, Kasaï Oriental, DRC, in 2021

Version 6 dated 05.01.2022

Administrative information

**TITLE**	**EfRAMAS project - Efficacy of a reduced dose of ready-to-use therapeutic food (RUTF) in children under 5 with uncomplicated severe acute malnutrition (SAM) compared to a standard dose: a controlled trial, randomized non-inferiority without additional staff in Bonzola and Nzaba health zones, city of Mbuji-Mayi, Kasaï Oriental, DRC, in 2021**
**Format modeled on the SPIRIT**	Internationally recommended format for clinical trials that will allow registration
**Recording of the test**	IRSCTN https://www.isrctn.com/ (site WHO)
**Protocol version**	Version 6 dated 05/01/2022
**Applicant / Sponsor of the trial**	Action against Hunger
**Funding sources**	FCDO
**Roles and responsibilities**	
**Main contributors**	
**PRONANUT**<br>**Scientific Co-Lead**	• Julien NTAONGO, principal researcher, doctoral student at the University of Kinshasa, technical expert at PRONANUT; j.ntaongo@gmail.com; Phone: +243826083356<br>• Jean-Baptiste MAYAVANGA, Statistics expert with PRONANUT; jbmayavanga@gmail.com; Phone: +243816511967
**University of Kinshasa**<br>**Thesis director**	• Mapunza Ma Miezi Samuel, Professor of Neuropsychiatry / Child Neuropsychiatry Unit;samuel.mampunza@unikin.ac.cd
ESP Kinshasa<br>Scientific lead	Marie-Claire MUYER, Professor, at ESP Kinshasa; muel_telo@yahoo.fr; Phone: +243811452711<br>Steve BOTOMBA, Assistant at ESP Kinshasa;botombasteve@gmail.com; Phone: +243816547701
ACF in Kinshasa<br>Administrative lead of the consortium	Marie PETRY, former Head of the Health Nutrition Department; mariep3@gmail.com<br>Uwimana SEBINWA, Head of Nutrition and Health Department;rddnut@cd-actioncontrelafaim.org; Phone: +243 81 70 86 218<br>Cédric KADIMA, Deputy Head of the Health Nutrition Department; rddnutadj@cd-actioncontrelafaim.org; Phone: +243 82 22 32 387<br>Victor NIKIEMA, RP Research Nutrition Health rpnsrech-mj@cd-actioncontrelafaim.org; Phone: +243824001998
ACF in Paris<br>Scientific co-lead	
Total duration of the study	Sophie BRUNEAU, Operational Technical Manager in Health Nutrition; sbruneau@actioncontrelafaim.org; Phone: +<br>Cécile SALPETEUR, Referent for research projects in Nutrition and Health; csalpeteur@actioncontrelafaim.org; Phone: +33 1 70 84 73 49

Executive summary

context

More than 47 million children suffered from acute malnutrition around the world before the COVID 19 pandemic. Disruption of economic systems, decline in access to health and nutrition services resulted in an increase of 20 % (or approximately 10.4 million new children) of the number of children who would suffer from acute malnutrition in 2021 mainly in low and middle income countries (Asia and Sub-Saharan Africa including the DRC). It is in this context that UNICEF, UNSCN, PAM and WHO insist on the immediate implementation of 5 urgent actions, including the early detection and treatment of wasting (acute malnutrition) in children. The treatment of uncomplicated SAM is community-based using systematic medical and nutritional treatment (distribution of Ready-to-Use Therapeutic Foods, RUTF) according to the strategy of community-based care of acute malnutrition (PCMA). Previous evaluations have shown that CMAM remains effective in the management of SAM despite low body weight gain, possibly due to inadequate practices (sale, sharing of RUTF, misuse of RUTF, lack of time to deal with patients. children ...), compared to treatment at the hospital. Studies (COMPAS, OPTIMA and MANGO) have tested the effectiveness of a reduced dose of RUTF under certain conditions and among different populations. The MANGO study confirmed the efficacy of a reduced dose in uncomplicated SAMs living in eastern Burkin Faso, under artificially ideal conditions. This is how the EfRAMAS project is proposed in order to assess the effectiveness of a reduced dose of RUTF without additional staff to collect data including a certain food insecurity in the DRC.

Goals

To assess the effectiveness of a reduced dose of RUTF on the speed of weight gain in children aged 6 to 59 months suffering from severe acute malnutrition, without medical complications in a context of food insecurity and without additional personnel to collect the data. The additional objectives are to evaluate the effect of the reduced dose of RUTF on the melting of edema, performance indicators (cure rate, abandonment rate, death rate, internal reference rate) and duration processing. In addition, the psychomotor development of children during the management of SAM, the degree of acceptance of the reduced dose by local communities and the food security situation of study participants will be assessed.

Intervention

The children admitted to this study will be divided into two groups: control and intervention. For the first two weeks, all children in both groups will receive the same standard dose of RUTF. From the third week, the dose of RUTF will be reduced for the intervention group according to their weight.

Study design

The EfRAMAS project is a randomized, controlled, non-inferiority study without additional staff to collect data and in a food insecure situation in children with SAM without medical complications. Children admitted to the study will be individually randomized into two groups, one receiving the standard dose (control) and the other the reduced dose (intervention). They will be closely monitored with weekly anthropometric and clinical measurements taken by the usual staff of the health centers and with the support of ACF supervisors, in order to assess the good progress of recovery.

Methodology

Anthropometric measurements including weight, height, MUAC and nutritional edema will be taken once a week on each child, from admission to discharge, using tools (scales, measuring rod, PB tape, …) Existing in health centers. The age and sex of the children will also be entered upon admission in order to help calculate the anthropometric indicators.

Psychomotor Development will be assessed by analyzing the skills and abilities acquired in the four main areas on admission, on discharge and then in the sixth month.

Food security will be assessed on admission according to the household food insecurity access scale (HFIAS) method proposed by the Food and Nutrition Technical Assistance Project (FANTA). Food and Nutrition).

Under study, a qualitative survey using focus groups, in-depth interviews and field observations will make it possible to assess the degree of acceptance of the reduced dose of RUTF by local communities and by nursing staff.

An economic evaluation of the cost of treatment will be made during the study by comparing the reduced dose approach to the standard dose approach.

Participants

All SAM children without medical complications from 6-59 months according to the national protocol (PT index <- 3 Z score and / or PB <115mm and / or bilateral edema (+, ++) as well as a positive appetite test) are eligible for the study. The information about the study will be explained to the parents or legal guardians in the local language, the latter will have the choice to refuse or to involve their children in the study. If the parents or legal guardians agree to have their children participate in the study, they will sign a free and informed consent. Parents or legal guardians refusing their children to participate in the study will have access to the uncomplicated SAM management program in force at the same health center. SAM children with complications, with negative appetite for RUTF, will be excluded from the study, or those whose parents or legal guardians have refused to participate. A unique identifier (ID) will be assigned to each enrolled child. The list of names of the children participating in the study with

their identifier will be kept under lock and key in each health center, in order to guarantee confidentiality throughout the study. MAS children will be recruited at the UNTA level, whether they come directly from the community or whether they are referred from preschool consultations (CPS). Community intermediaries will ensure awareness-raising on acute malnutrition in order to facilitate recruitment on time. The list of names of the children participating in the study with their identifier will be kept under lock and key in each health center, in order to guarantee confidentiality throughout the study. MAS children will be recruited at the UNTA level, whether they come directly from the community or whether they are referred from preschool consultations (CPS). Community intermediaries will ensure awareness-raising on acute malnutrition in order to facilitate recruitment on time. The list of names of the children participating in the study with their identifier will be kept under lock and key in each health center, in order to guarantee confidentiality throughout the study. MAS children will be recruited at the UNTA level, whether they come directly from the community or whether they are referred from preschool consultations (CPS). Community intermediaries will ensure awareness-raising on acute malnutrition in order to facilitate recruitment on time.

A total of 1000 SAM children aged 6-59 months will be recruited from 14 UNTAs for this study. Randomization will be individual and will be done at the level of each UNTA.

Abbreviations

ACF	Action against Hunger
IYCF	Nutrition for Young Children
RUTF	Ready-to-use therapeutic food
CS	Health center
CPS	Preschool consultation
DPM	Psychomotor Development
DSMB	Data and Safety Monitoring Board - patient safety committee (1 nutrition specialist + 1 biostatistician)
ESP	School of Public Health
AND	Standard deviation (SD)
IT	Registered Nurse
ID	Username
MANGO	*Modeling an Alternative Nutrition protocol Generalizable to Outpatient,*Name of the Burkina research project that demonstrated the effectiveness of a reduced dose of RUTF
MAS	Severe Acute Malnutrition
MCZS	Head of Health Zone
WHO	World Health Organization
NGO	Non Governmental Organization
PT	Weight - Size index
PB	Brachial Perimeter
PCIMA	Integrated Management of Acute Malnutrition
PRONANUT	National Nutrition Program
DRC	Democratic Republic of Congo
RDPM	Psychomotor Developmental Delay
RECO	Community Relays
Unicef	UNICEF
UNTA	Outpatient Therapeutic Nutritional Unit
UNTI	Intensive Therapeutic Nutritional Unit
ZS	Health Zone

Contents

Administrative information....2
Executive summary....3
I. Introduction....10
1.1. Context and rationale....10
1.2. Purpose and Objectives....13
1.3. Research hypotheses....14
1.4. Trial design....14
II. Methodology....16
2.1. Participants, interventions and outcomes....16
2.1.1. Study framework....16
2.1.2. Participants....18
2.1.3. Interventions....19
2.1.4. Results....24
2.1.5. Places of inclusion of children....25
2.1.6. Sample size....26
2.1.7. Recruitment....26
2.2. Randomization....27
2.2.1. Sequence generation....27
2.2.2. Randomization concealment mechanisms....27
2.2.3. Implementation....27
2.2.4. Blinding (masking)....27
2.3. Data collection, management and analysis....28
2.3.1. Data collection methods....28
2.3.2. Data managment....37
2.3.3. Statistical analyzes....37
2.4. Monitoring....38
2.4.1. DSMB Committee (Data and Safety Monitoring Board).....**Erreur ! Signet non défini.**
2.4.2. Prejudices....38
III. Ethical considerations and publications....40
3.1. Authorization from the research ethics committee....40
3.2. Protocol amendments....40
3.3. Free and informed consent or assent....40
3.4. Confidentiality....41
3.5. Declaration of interests....41
3.6. Data accessibility....41

3.7. Ancillary and post-test care 41
3.8. Publication policy 42
IV. Project risk analysis and mitigation measures 43
V. Budget 44
VI. Chronogram of activities 45
VII. Bibliographical references 46
VIII. Appendices 49
Annex 1: Information for participants 49
Annex 2: Informed consent form 52
Annex 3: Assessment of psychomotor development 53
Annex 4: Acceptability study of a reduced dose of RUTF for the management of SAM children 60
Annex 6: Typology of Health Areas 84
Annex 7: Distribution of participants in 10 UNTAs by ZS .... **Erreur ! Signet non défini.**
Annex 8: List of variables according to specific objectives 85

Table of illustrations

List of paintings

Table 1: Health situation, Nzaba and Bonzola health zones, January and February 2021 17
Table 2: RUTF dose per group in number of sachets per week 19
Table 3: Criteria for interrupting or modifying the study protocol .... **Erreur ! Signet non défini.**
Table 4: Criteria for leaving the study outside of the cure 21
Table 5: Medical treatment of children admitted to the study 22
Table 6: Results of the appetite test 23
Table 7: Health areas selected for the study 26
Table 8: Sources of funding for the study at the date of the protocol 44

List of Figures

Figure 1: Design selection, randomization and monitoring of participants 15
Figure 2: Study site map, ZS Bonzola and Nzaba, Kasaï Oriental, DRC 18

I. Introduction

1.1. Context and rationale

More than 47 million children suffered from acute malnutrition around the world before the COVID 19 pandemic (1) mostly in low- and middle-income countries, particularly in Africa and Asia(2). According to estimates, around 10.4 million new children will suffer from acute malnutrition in 2021 mainly in the Middle East (Yemen) and Sub-Saharan Africa including the Democratic Republic of Congo (DRC)(3) (4). The DRC will bear the heaviest burden because the country will account for more than 30% of this total, i.e. around 3.3 million children are said to be suffering from acute malnutrition as of now.(4). This increase would be due to the negative effects of confinement, even of short duration: disruption of economic systems with a reduction in GDP per capita of 6 to 7% (standard deviation 2-4%), decrease in access to health services and nutrition in low and middle income countries(3) (5).

The increase in the number of children suffering from acute malnutrition would be accompanied by an approximately 25% reduction in access to health and nutrition services, experts estimate there will be more than 128,605 additional deaths in children under 5, more than 52% of which came from Sub-Saharan Africa (6).

The consequences of the pandemic would also lead to an increase in the number of people living under the extreme poverty line, approximately 140 million additional individuals. (5) and lead to a situation of acute food insecurity reaching 265 million people (7). Access to basic social services, including health and nutrition, has also experienced a sharp decline following the COVID 19 pandemic, like what happened with the great Ebola epidemic in Africa. the west(8).

It is in this context that the leaders of four United Nations agencies (UNICEF, FAO, WFP and WHO) launched an urgent appeal "Child malnutrition and COVID-19: the time to act is now"(9)in which they propose 5 urgent actions to be implemented to limit the impact of COVID-19 on infant nutrition: i) Safeguard and promote access to nutritious, safe and affordable food; ii) Invest in improving maternal and child nutrition during pregnancy, childhood and early childhood; (ii) Reactivate and strengthen services for the early detection and treatment of wasting and the treatment of wasting in children; iv) Maintain the provision of nutritious and safe school meals for vulnerable children; and v) Develop social protection to ensure access to nutritious food and essential services(9).

The 3rd recommendation of the heads of the United Nations agencies concerns the early detection and treatment of wasting (acute malnutrition) in children(9). This work is only interested in severe acute malnutrition (SAM) which is defined by a very low weight-for-height index (PT) lower than –3 z-scores at the median, indicative of severe and visible wasting, and / or by a low MUAC of less than 115 mm and / or by the presence of bilateral nutritional edema on the feet(10). Acute malnutrition occurs when the body is deprived of calorie and micronutrient intake due to insufficient food intake, poor nutrient absorption due to illness or anorexia, for a relatively short time. Children under five are most vulnerable to malnutrition(11) because of their nutritional needs increased by growth and immunity not yet acquired in the face of various common pathologies (respiratory, digestive).

The management of acute malnutrition has two components: systematic medical treatment and nutritional treatment. In addition to routine antibiotic therapy and deworming, medical treatment includes vaccination against measles and vitamin A supplementation.(12). In addition, any childhood illness identified on admission such as malaria, scabies, diarrhea, etc. receives appropriate treatment during the treatment of SAM.

The nutritional treatment of severe acute malnutrition consists essentially of the administration of ready-to-use therapeutic foods (RUTF). For children who suffer from SAM without complications, the management is done on an outpatient basis with RUTF, a peanut paste enriched with powdered milk, vegetable oils, sugars, vitamins and minerals.(13) (14), according to the strategy of integrated management of acute malnutrition (PCIMA) unlike previous protocols where children were treated with F75 and F100 milks in a hospital environment (15) (16) (14).

A series of bottleneck analyzes (17)carried out in the West and Central Africa region in 2018 documented key issues regarding the delivery and uptake of outpatient SAM care. Major issues include lack of trained human resources, support costs, long waiting times, stockouts and geographic access. RUTF stock-outs are one of the obstacles to accessing and participating in the PCIMA program. These ruptures would be due to the large volume and the difficulties encountered in the transport of RUTF(18). In the DRC, for example, only 15 to 20% of children suffering from acute malnutrition, i.e. 495,000 to 660,000, could have access to acute malnutrition treatment services.(19). Because all of the countries (73 in total) that benefit from the PCIMA program are totally dependent on UNICEF or the Clinton Foundation, it is difficult

to ensure the sustainability of the PCIMA. The high cost of RUTF (around 50% of the total cost of PCIMA), would prevent respective governments from ensuring their own supply(20).

It is in this context that the actors in the field have implemented adaptations of the protocol to face the constraints encountered. In Niger, MSF distributed 2 sachets of RUTF (1000 kcal) to more than 328 children aged 6 to 59 months, of whom more than 90% were aged 12 to 35 months. The beneficiaries showed a weight gain of 9.8 (± 4.6) g / kg / day for an average duration of treatment of 29 (± 13.5) days(21). This was also the case during an intervention in Burma in 2009, the NGO Action Contre la Faim (ACF) provided treatment for severe acute malnutrition without medical complications with reduced doses of RUTF. Until children reached a PT index ≥ -3 and MUAC ≥ 110 mm, the dosage was based on the child's weight. Afterwards, 500 kcal / day or 1 sachet of RUTF per day (until discharge) was distributed to each child regardless of their weight. A weight gain velocity of 4 g / kg / day (3 to 5.7 kg) was observed for each child for an average duration of treatment of 42 days (28 to 56 days). This experience in Burma shows that it is possible,(22). It should be noted that in practice it is observed the sale or sharing of RUTF distributed to children within the framework of the PCIMA.(23). This implies that children sometimes actually consume less RUTF than what is distributed.

The evaluation carried out on 3083 children in Myanmar between 2009 and 2010 showed that a reduced dose of RUTF, associated with routine care, allows to have the same effectiveness as the standard dose. In the outlook, this study recommended carrying out similar but randomized research to verify the effectiveness of a reduced dose of RUTF for the management of SAM.(22).

The recent results of the MANGO study, carried out in Burkina-Faso, showed that the reduction in the dose of RUTF after two weeks of treatment of children suffering from SAM, defined by PT <-3zscore and / or MUAC <115 mm and no edema at all, reduced neither weight gain (3.4 g / kg / day; 95% CI -0.4 to 0.4; p = 0.92) nor rate of girth gain brachialis (BP), does not affect healing or prolong the duration of treatment (with a median of 56 days; interquartile range [IQR] 35-91) although a small negative effect (0.2 mm / week; 95% CI: 0.04 to 0.4; p = 0.015) was observed on linear growth especially for the youngest subjects (24). This study was able to show that children with a reduced dose of RUTF had the same body composition as those who received the standard dose, and came out cured with a fat-free mass almost at the same level as that of healthy children in the community.(25). In terms of energy, reducing the dose

of RUTF leads to a reduction in the daily intake but nevertheless makes it possible to cover the daily energy needs of children treated for uncomplicated SAM.(26).

The MANGO study demonstrates that a reduced dose of RUTF would be as effective as the standard dose in the management of uncomplicated SAM under ideal conditions. It is in this context that we propose to carry out the EfRAMAS project to confirm this in a situation of food insecurity and without additional staff to collect data on children who suffer from SAM in the DRC.

1.2. Purpose and Objectives

Goal

The aim of this study is to help improve the management of severe uncomplicated acute malnutrition.

Goals

a. Main objective

To assess the effectiveness of a reduced dose of RUTF in children 6 to 59 months with SAM (defined by PT <-3zscore and / or MUAC <115mm and / or edema +, ++) without complications in a context of 'food insecure and no additional staff to collect data.

b. Specific objectives

Primary objective

To assess the effectiveness of a reduced dose of RUTF in the management of SAM in children aged 6 to 59 months on the rate of weight gain (g / kg / d) from admission to discharge .

Secondary objectives

To assess the effectiveness of a reduced dose of RUTF in the management of SAM in terms of:

- **Duration of edema melting** (in days) since admission.
- **Duration of stay** (in days);
- **Performance indicators (cure rate, dropout, mortality, non-respondent, transfer to hospital)**
- **Treatment cost savings** (average cost and total cost per child treated);
- The growth velocity of anthropometric indices
- **Psychomotor development** children
- **Degree of acceptance** a reduced dose of RUTF by healthcare workers and the community
- **Relapse rate** up to 3 months after the “cured” exit from the program.

Research hypotheses

Main hypothesis

Children receiving the reduced dose will have the same weight gain from admission to discharge as children receiving the standard dose of RUTF with a non-inferiority margin of 0.5g / kg / day.

Secondary assumptions

- Children receiving the reduced dose of RUTF will have the same performance indicators (cure rate, death, drop-out, relapse) as those receiving the standard dose;
- Children receiving the reduced dose of RUTF will have the same length of stay as those receiving the standard dose;
- Children receiving the reduced dose of RUTF will have the same psychomotor development as those receiving the standard dose;
- Treatment with a reduced dosage of RUTF will be less expensive than treatment with the standard dose.

1.3. Trial design

The EfRAMAS project is a randomized, controlled, non-inferiority clinical trial. Randomization will be done at the individual level in each of the 14 UNTAs selected for the study.

Bulk randomization of variable size will be used. After randomization, the participants will be distributed randomly either in the control group or in the intervention group.

Each participant will receive the dose of RUTF corresponding to the group to which he belongs: standard dose for the control group and reduced dose for the intervention group (Table 2).

Apart from nutritional support, participants will also receive systematic medical treatment (Table 5).

After recovery (according to the admission criteria, if PT ≥ - 1.5 z-score at two consecutive weighings, and PB ≥ 125 mm and absence of nutritional edema for 14 days), follow-up of participants will be ensured every 15 days up to 3 months after discharge as provided for in the national protocol (12). During this follow-up, the children will receive medical treatment if necessary according to the symptoms (malaria, etc.) and a nutritional product (fortified flour or ASPE) if available. Children who develop complications during treatment will be referred to UNTI and then return to their respective study group after the complication has been resolved.

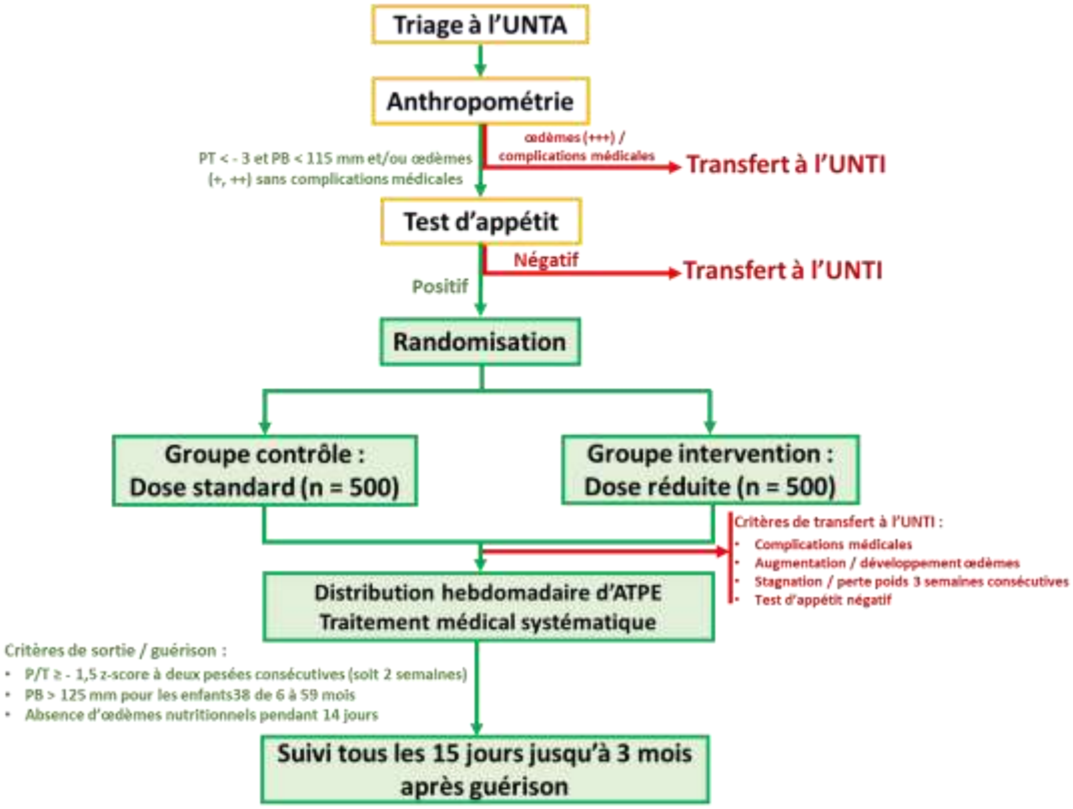

Figure 1 : Design selection, randomization and monitoring of participants

II. Methodology

2.1. Participants, interventions and outcomes

2.1.1. Study framework

The EfRAMAS project will be carried out in 14 UNTAs in the health zones of Bonzola and Nzaba, in the province of Kasai Oriental in the DRC.

Kasai Oriental is part of the provinces of the greater Kasai area, which is made up of 5 provinces. In 2017, the area of Greater Kasai was the victim of a conflict between the militiamen of Kamwina Nsapu and the Armed Forces of the DRC. Starting from the province of Kasai Central, the conflict spread to affect all the provinces of the Grand Kasai area. It is one of the bloodiest conflicts in the Greater Kasai area, with more than 5,000 dead, 1.4 million displaced people and more than 600 schools and health centers destroyed.(27) (28) (29). In addition to schools and health centers, the militiamen loot and ransack everything they cannot take.

More than 70% dependent on agriculture, the population of Kasai loses not only crops (for seasons A, September - December 2016 and season B, February - July 2017) but also tillage equipment. This resulted in an inflation of more than 100% with as a corollary the worsening of malnutrition of children under 5 and pregnant women.(30) (31). According to forecasts from the integrated analysis of food and nutritional security (IPC) carried out in September 2020, more than 31% of the population of Kasai Oriental will be in a situation of very high food insecurity by June 2021.(32).

These two ZS are located in the city of Mbuji Mayi, capital of the province of Kasai Oriental (Figure 1). The Bonzola ZS has 15 health centers (CS) and 1 General Reference Hospital and the Nzaba ZS has 18 health centers (CS) and 1 General Reference Hospital. Each ZS has an UNTA. The average admission per month was 401 and 635 MAS children respectively in the ZS of Bonzola and Nzaba, at the start of 2021. With the exception of the 2 UNTAs in Bonzola and 5 in Nzaba, all these UNTAs are functional and organize screening. active. Regarding staff training, a total of 32 (14 women and 18 men) and 27 (16 women and 11 men) providers respectively in Bonzola and Nzaba are trained on PCIMA. For the Integrated Management of Childhood Illnesses (IMCI), only 4 providers (1 woman and 3 men) are trained in the Bonzola health zones only. A man and a woman are trained on Reproductive Health in Bonzola against two men and a woman in Nzaba. Of the 1,241 Community Relays in the ZS de Bonzola, 1,004 of them or 81% are active, including 125 trained in active screening, 110 in ANJE and 11 in

maternal PB. In Nzaba, only 662 of the 1290 RECOs are active, including 10 trained in active screening and 30 in IYCF.

The DRC has not been spared by the COVID pandemic. It has recorded a cumulative 36,576 confirmed cases and 1 probable case as of June 16, 2021 since the epidemic was declared on March 10, 2020, including 857 deaths, i.e. a case fatality of 2.3%. Fortunately, the province of Kasai Oriental is not one of the most affected provinces. It has recorded 24 confirmed cases since the start of the pandemic and has experienced a period of at least 29 days without recording cases (Situation report n 167/2021 of 16/06/2021 from the Ministry of Health).

In the third quarter of 2020, the ZS of Bonzola and Nzaba reported respectively i) 33% and 30% of children under 5 years of age malnourished with a MUAC <125 mm according to data from the sentinel sites and ii) 23% and 26 % of malnourished pregnant women with a MUAC <230mm, ie these two areas are reported on nutritional alert in the SNSAP bulletin of August 2020. The two health areas are priority n ° 2 of the revised humanitarian response plan (33).

In January and February 2021, the health situation in the ZS is summarized by the following table:

Chart 1 : Health situation, Nzaba and Bonzola health zones, January and February 2021

**Indicators**	**ZS Bonzola**	**ZS Nzaba**
Acute respiratory infection in children <5 years	314 (1%)	895 (2%)
Malaria (Attack rate) in children <5 years	2,629 (7%)	2,986 (5%)
Diarrhea (Attack rate) in children <5 years	4,104 (10.20%)	7,644 (11.64%)
Measles (Attack rate) children <5 years	4	8
HIV in the general population	3.99%	2.25%
Women who adopted FP	31.42%	12.51%
Mothers / caregivers sensitized to IYCF	52.64%	81.12%
Exclusive breastfeeding in children <6 months	70.69	93.4
IMCI access for children <5 years old	96.59%	69.52%
Anti-measles vaccines for children <5 years	1,164 (3%)	2,080 (4%)
Vit A (dose) children <5 years	9,150	16,750
MAS in 2020 (SMART ACF month, 2020)	2,382 (7%)	2,106 (4%)

Source: Data from the ACF survey on Mbuji Mayi (2020)

The populations of these 2 ZS live mainly from agriculture and artisanal diamond mining (95%). Geographical accessibility (road and air) is relatively good and there is good telephone coverage. The province experiences two periods of welds (February - March and September - November). The population has poor access to drinking water.

At the same time as the EfRAMAS project will take place, ACF will lead a support intervention to the Ministry of Health for the management of acute malnutrition in these 2 HZs.

The main axes of this technical support are:

- Integrated Management of Severe Acute Malnutrition (PCIMA);
- Integrated Management of Childhood Illnesses (IMCI);
- Family planning ;
- Prenatal Consultations (ANC);
- Post-Natal Consultations (CPON).

In order to materialize its support, ACF organizes the following activities:

- Capacity building of health care providers;
- Bonuses for the functioning of health facilities and the encouragement of service providers;
- Strengthening of the community component by giving bonuses to community relays;
- Support for trained providers;
- Provision of care monitoring tools;
- Supply of inputs including RUTF, drugs etc.

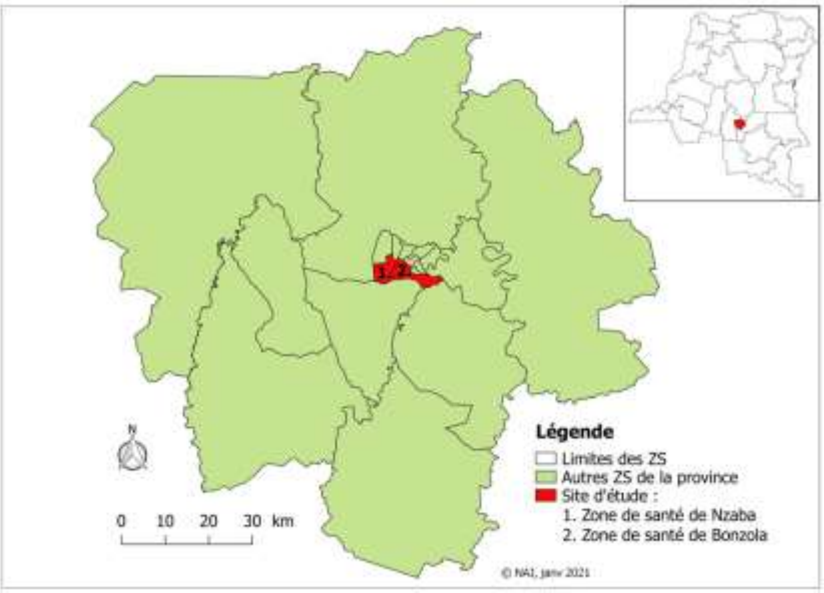

Figure 2 : Study site map, ZS Bonzola and Nzaba, Kasaï Oriental, DRC

Apart from ACF, other partners have supported or are supporting the ZS of Bonzola and Nzaba.

2.1.2. Participants

a. Inclusion criteria

To participate in this study, individuals must meet the following criteria:

- Be between 6 and 59 months old;

- To be MAS: PT <-3 Z-score and / or PB <115mm and / or bilateral edema (+, ++);
- Not having any medical complications;
- Have a good result on the appetite test;
- Live in the selected health zone;
- Consent to participate in the study (informed consent);
- Not have other siblings already admitted to the study. When there are several participants in the same siblings, only the first will be included in the EfRAMAS project. The others will be covered according to the national program but will not be part of the study.

Non-inclusion criteria

- Appetite test inconclusive (negative test)
- Have another sibling member (MAS) already admitted to the study;
- Have a declared allergy to peanuts and / or milk;
- Have received treatment for SAM in the past 6 months, including readmissions after program drop-out, relapses and medical transfers;
- Malformation or disability or chronic pathologies that may affect food intake, such as a cleft palate, cerebral palsy, trisomy 2, sickle cell anemia ...

2.1.3. Intervention

As part of the EfRAMAS project, the intervention will consist of the management of SAM with all the care provided for in the national protocol. The only difference will be in the dosage of RUTF over time. Both groups will receive the same dose of RUTF for the first two weeks. Then in the intervention group, the reduction in the dose of RUTF will take place from the third week of treatment (Table 2). The intervention will begin concomitantly in both groups.

Chart 2 : Dose of RUTF per group in number of sachets per week

Weight (kg)	Standard dose control group		Reduced dose intervention group			
	Admission-Discharge		Weeks 1-2		Week 3 - discharge	
	Bags / week	Kcal / kg / d	Bags / week	Kcal / kg / d	Bags / week	Kcal / kg / d
3.0-3.4	9	168-190	9	168-190	7	147-167
3.5 - 4.9	11	183-204	11	183-204	7	102-143
5.0 - 6.9	14	155-214	14	155-214	7	72-100
7.0 - 9.9	21	144-204	21	144-204	14	101-143
10.0 - 14.9	28	144-214	28	144-214	14	67-100

When a participant does not respect his date of appointment, the day he presents for the consultation, he will receive the number of sachets in proportion to the number of days remaining before the next appointment.

a. Criteria for transfer to UNTI

In accordance with the criteria set in the PCIMA protocol, the intervention will be interrupted and the child transferred to the UNTI if he finds himself in one of the following cases:(12) :

On admission:

- Any child under 6 months with a weight <3.5 kg;
- Mixed forms of malnutrition (marasmus and kwashiorkor);
- Presence of generalized edema (+++);
- If the appetite test fails (poor appetite);
- Presence of signs of medical complications.

During the weekly consultation:

- Increase / development of nutritional edema;
- If the appetite test fails (poor appetite);
- Appearance of signs of medical complications;
- Appearance of re-nutrition diarrhea leading to weight loss;
- Presence of one of the "treatment failure" criteria:
    - Weight loss for 2 consecutive weeks;
    - Weight loss of more than 5% of body weight at any one visit;
    - Weight stagnant for 3 consecutive weighings.

All the children admitted to the study and who will be transferred to UNTI, will then return to research in their respective groups. On their return, the children in the reduced dose arm will resume the planned dose the week they were transferred, so they do not restart the treatment from zero but continue it.

b. Modification or interruption of the intervention

During the implementation of this study, the intervention will be interrupted for each participant who presents (12):

- Withdrawal of informed consent;
- Discontinuation of treatment;
- Lost view ;
- Failure to process.

Chart 3 : Criteria for leaving the study outside of the cure

Criteria	Definition
Abandonment-confirmed	Patient absent for two consecutive weeks (declared abandonment in the third) sought by the RECOs and confirmed as having abandoned
Unconfirmed abandonment	Patient absent for two consecutive weeks (declared abandonment in the third) but his actual method of discharge (abandonment or death) not having been confirmed
Death	Participant whose death has been certified by a nursing staff
Lost view	Change of address, followed in another CS outside the study area
Treatment failure	Referred to UNTI following the appearance of danger signs (temporary discharge *)
Withdrawal of consent	Any time after randomization
Non-response to treatment (even if very rarely)	Children who do not recover after 12 weeks of treatment

*****_AT their return the children of the arm with reduced dose will resume with the dose planned for the week in which they were transferred, so they do not restart the treatment from zero but continue it_.

c. Strategies to improve protocol adherence

Every evening, the principal investigator, the ACF Research RP and the three data collectors check all the data collected in the field in the different UNTAs. Inconsistencies, outliers or missing information for certain variables are noted and communicated to ACF supervisors who follow up within 24 hours and strengthen the capacities of UNTA agents, and also to RECOs. During their visit to enter data, data entry clerks also communicate certain weaknesses observed to healthcare providers with a view to correcting the data as far as possible.

In some cases where trained providers have moved or left their posts, other providers will be trained by the research team to ensure the continuity of the study.

For each child who does not respond to an appointment, RECO makes a home visit to find out what is going on.

d. Systematic treatment (Relevant concomitant care and interventions)

All children admitted to the study, regardless of their group, will receive systematic medical treatment as shown in the table below:

Chart 4 : Medical treatment of children admitted to the study

Medications	Weight (kg)	Dosage (mg)	Number caps / tab (250 mg)
**Amoxicillin** (50-100 mg / kg / day) for 7 days on admission Dosage: 2 times a day	<5	125 mg * 2	1/2 cap * 2
	05 to 10	250 mg * 2	1 cap * 2
	10 to 20	500 mg * 2	2 cap * 2
	20 to 35	750 mg * 2	3 cap * 2
	> 35	1000 mg * 2	4 cap * 2
**Medications**	**Age**	**Dosage**	
**Albendazole** At the 4th week if not received in the past 6 months	<1 (years)	Do not administer	
	1 to 2 (years)	1/2 tablet	
	≥ 2 (years)	1 tablet	
**Vitamin A** At the 4th week if not received in the past 4 months	6 to 11 months	100,000 IU	
	≥ 1 month	200,000 IU	

e. Nutritional measurement and monitoring

Chart 5 : Nutritional monitoring and taking measures

Observations	Frequencies
MUAC measurement	Every week
Weight measurement	Every week
Search for edema	Every week
Appetite test	Every week
Temperature measurement	Every week
Complete clinical examination	Every week
Size measurement	Every week
Calculation of the PT index (z-score)	Every week
Nutrition education	Every week
Assessment of RUTF consumption	Every week
Home visit	Depending on the case: if absence or no answer
Vaccination	Complete the vaccination schedule at the 4th week
Assessment of psychomotor development	On admission, discharge, and 6 months after recovery
Vaccination	Complete the vaccination schedule at the 4th week of follow-up

At each visit (week), the mothers / carers of the children benefit from a nutritional equation session, the essential points of which are:

- RUTF is a medicine intended exclusively for the management of children with severe malnutrition and therefore it should not be shared or sold;

- Do not force the child to eat. One sachet of RUTF can be consumed in several meals;
- The child should always be given drinking water when taking RUTF;
- For children who are breastfeeding, breastfeed the child before giving RUTF;
- RUTF meets the daily nutritional needs of the child, always give it before other foods (for children who are not under breastfeeding);
- The accompanying person should wash their child's hands and face with soap before giving RUTF;
- Specify to the accompanying person the amount of RUTF that their child must eat per day;
- The treatment must be respected: for a good recovery, the child must be consulted every week for a minimum period of 1 month and a half;
- The accompanying person must return the empty sachets of RUTF and the sachets not consumed to the UNTA
- caregivers and family members should not consume RUTF instead of the child, even for reasons of breastfeeding or feeding other children.

Chart 6 : Appraisal of the appetite test based on the proportion of dough consumed

**Body weight (kg)**	**RUTF - paste in sachet** (Proportion of a whole bag 92g)		
	**Weak**	**Average**	**Good**
<4	<1/8	1/8 - 1/4	> 1/4
4 to 6.9	<1/4	1/4 - 1/3	> 1/3
7 to 9.9	<1/3	1/3 - 1/2	> 1/2
10 to 14.9	<1/2	1/2 - 3/4	> 3/4
15 to 29	<3/4	04/03 to 1	> 1
> 30	<1	> 1	

To have good results in the appetite test, the following measures must be observed:

- Wash your child's hands and face and hands before giving RUTF;
- Obtain a cup of drinking water;
- Find a comfortable place;
- Do not force the child to eat;
- Check if the child has eaten in the last 30 minutes.

2.1.4. Results

a. Main result

The main outcome of this study will be the velocity of weight gain for each participant in the two study groups (control and intervention group). The acceptable weight gain is 5 g / kg / day for each participant regardless of the group to which he belongs. This weight gain is an average calculated between the gain on admission and on discharge for all the children admitted to the study (intention to treat) and then only for those who are declared cured (per protocol).

To conclude that there is no inferiority, it is assumed that the reduced dose will not be less than the standard dose if the difference in weight gain is less than 0.5 g / kg / day. Therefore, participants in the intervention group should not show a weight gain <4.5 g / kg / day to conclude that the reduced dose of RUTF was not inferior.

b. Additional results

The additional results expected in this study, in the two groups (control and intervention), are:

- **The duration of the edema melting**: calculated in number of days, this indicator corresponds to the time taken for the lower limbs of SAM children to return to their normal volume. The expected result is the onset of melting of the edemas within the first 14 days after admission and their complete disappearance after 21 days of treatment.
- **The length of stay**: calculated in number of days, this indicator corresponds to the time elapsed between admission and discharge (PB≥12.5cm and / or PT ≥-3 Z score and / or edema melt). The maximum length of stay is 12 weeks or 3 months(12).
- **The cure rate:** A child will be considered cured if he meets the cure criteria according to his admission category: A PT index ≥ -1.5 or PB ≥125 mm during two consecutive visits and no edema during two visits (2 consecutive weeks.
- **The dropout rate:**it is the proportion of children confirmed alive who missed two successive visits. The abandonment is confirmed once after the visit of the RECO which confirms that the child is alive despite his 2 absences during the weekly visits. Children who have abandoned will be followed up to 3 months after the abandonment. For children who can be found, offer the caregivers a small snack at the single appointment 3 months after abandonment to take the anthropometric measurements.
- **Death rate :** the proportion of children randomized to the study who died during the intervention at any time.
- **Cost savings** (average cost saved and cost saved per child treated): this involves comparing the cost of treatment for children in care and declared cured in the two groups. We will use a societal perspective including the direct costs of drugs and RUTF

(purchase, transport, transport and storage) to assess the cost for each child, the HR costs involved in the care, the non-medical costs (food and transport. beneficiaries), indirect costs (loss of productivity).

- The growth velocity of the anthropometric variables and indices: it will be a question of evaluating the prevalence of stunting, underweight of the children included in the study in the two groups at the end of the treatment.

4) Secondary outcomes

- **Acceptance** of a reduced dose during SAM treatment by healthcare staff as well as the community: this involves assessing the perceptions of families and healthcare providers on the amount of RUTF received, consumption practices and 'uses and their attitude to dose reduction.
- **The relapse rate:** this is the proportion of children in care for SAM and declared cured who become SAM again in the 3 months after the end of treatment.
- **Psychomotor development** : This sub-study aims to assess the proportion of children with psychomotor developmental delay (DPM) and to monitor their progress during treatment. Psychomotor Developmental Delay (RDPM) is understood to mean disorders in the acquisition of skills and competences in the areas of language, cognitive, motor and social interaction. The RDPM corresponds to a score of less than 70% in two of the four domains of the DPM(34). For this sub-study, it is expected that the MPD of children with RDPM will improve after their management.(35) (36) (37) due to the simple fact of the medical and nutritional intervention without particular action on the DPM during the treatment.

2.1.5. Places of inclusion of children

A total of 14 UNTAs will be selected for this study due to 7 UNTAs per health zone. The UNTA selection criteria are as follows:

- Have a good attendance: the selection will start from the most frequented UNTAs to the less frequented;
- Be geographically accessible;
- Be covered by a telephone network.

The table in appendix 6 shows the elements of the UNTA typology including the average monthly attendance, the percentage of malnourished children admitted per month, the number of population served by the health area, the number of trained staff in post, the number of days disruption of inputs (average per month), the days of PCIMA activity.

Chart 7 : Health areas selected for the study

Bonzola health zone		Nzaba health zone	
1	Mudiba	8	Of the market
2	Solola	9	Tudikolela
3	Tubondo 2	10	PMKO
4	Dubai	11	Lutulu
5	Kashala Bonzola	12	Airport
6	Nyongolo	13	Tarmac 2
7	Lubilanji	14	Tatu muya

2.1.6. Sample size

The size needed for this work will be calculated according to the following formula:

$$N = (Z1\text{-}\alpha + Z1\text{-}\beta)^2 \times 2\sigma^2 / (\Delta - \delta)^2$$

Or :

α represents the error of the first kind; β, error of the second kind; Δ, the Non-inferiority Margin; σ^2, Estimation of the variance and δ, Possible difference in expected efficacy between the standard dose and the reduced dose. As $\alpha = 0.05$ then $Z1\text{-}\alpha = 1.64$ and $Z1\text{-}\beta = 0.84$(38) (39) (40).

In this study the mean rate (SD) of weight gain for each participant is 5 g / kg / day (2.6) and the expected difference between the two groups (control and intervention groups) will be 0 g / kg / day with a non-inferiority of 0.5 g / kg / day. The minimum acceptable average rate of weight gain in any group will be 4.5 g / kg / day. Assuming a power of 80% and a significance level of 5%, 335 children are needed in each group to verify that the maximum true difference between the intervention groups is not more than 0.5 g / kg / day . Taking into account a dropout rate of 20%, the total sample size was rounded to 1,000 children.

2.1.7. Recruitment

Patients will be recruited from the UNTAs whether they come directly from the community, screened by the RECOs or whether they are referred from the HCs. Community intermediaries will ensure awareness in order to facilitate recruitment within the allotted time. We plan to enroll 2-3 children per day per UNTA. Which will take about 2 months for the recruitment.

On admission, anthropometric measurements including weight, height, MUAC and nutritional edema will be taken on all children. The gender and age of the children will also be entered upon admission.

On admission, for each child, in addition to anthropometric measurements:

- Psychomotor development will be assessed by a score;
- A socio-demographic questionnaire including a food insecurity score will be administered to the caregiver.

All participants will be recruited on the basis of informed consent of information given in the language of the caregiver, followed by a written and signed agreement. Confidentiality will be ensured for each individual who will be selected for the study via the anonymization of questionnaires and data protection.

2.2. Randomization

2.2.1. Sequence generation

Randomization by blocks of variable size will be used in this study in each of the 14 UNTAs. In order to ensure that the participants are evenly distributed, in each block there will be the same number of children from each group(41) (42).

2.2.2. Randomization concealment mechanisms

Once the randomization lists are generated, they will be printed and kept in two copies, one at the ACF headquarters in Paris and the other at the ESP in Kinshasa. The randomization list for each UNTA will be sent by sealed envelope to the nurse in charge of the corresponding health area (IT). Researchers will not have access to the footage. Only ITs will have access to the randomization sequences of the corresponding UNTA.

2.2.3. Implementation

Randomization will be done using www.randomization.com by an independent person who will not participate in the implementation of the study. Study participants will be recruited from the 14 UNTAs (due to 7 per ZS) selected and upon admission each will receive a unique identifier corresponding to their group in the study. The IT will receive special training to enable it to know how to randomly assign the identifier to UNTA participants.

2.2.4. Blinding (masking)

The statistician who will do the randomization will send the results in closed envelopes which will only be opened by the ITs of each selected UNTA. Neither the nurses who will take the anthropometric measurements nor the researchers will know which group each child

participating in the study belongs to. Due to the differing amounts of total RUTF sachets distributed, parents may deduct the dose their children will receive and thus not be blinded.

2.3. Data collection, management and analysis

2.3.1. Data collection methods

a. Data collection technique

Quantitative data : Anthropometric equipment (scales, measuring rod, tape measure) will be used to take the anthropometric measurements of the children. Children will only be measured once for each parameter (weight, height, PB, etc.) as in current practice.

Clinical data will be collected during an interview with mothers followed by a physical examination at each weekly consultation.

The socio-demographic and economic characteristics of the children and their families will be collected during structured interviews using a questionnaire taken on enrollment in the study.

The questionnaire will be sent in the language of the family caregiver by the existing local IT.

Qualitative data: the focus group, in-depth interviews and field observations will be used for the collection of qualitative data.

b. Data collection plan

Frequency of data collection	Admission	Weekly treatment max 12 weeks	Dump	Monthly follow-up +3 months after discharge	Monthly follow-up at +6 months after discharge
Selection for inclusion in the study	X				
Information, consent and randomization	X				
Sociodemographic questionnaire	X				
Physical examination	X	X	X		
Anthropometric measurements	X	X	X	X	X
Distribution of RUTF	X	X			
Psychomotor Development Score (DPM) Assessment	X		X		X
Individual interview for acceptability study					
FGD					

Staff recruitment and training

The study should be organized under conditions as close as possible to routine practice. As a result, it is the affected providers (2 per UNTA) in the selected health centers who will collect the main data. The ACF nutrition supervisors will be called upon to supervise the UNTAs and ensure that the strict application of the SAM treatment protocol is observed. The researchers will ensure the training of providers and the quality control of the data for the proper conduct of the study for the data collection phase. Only interviewers for the qualitative part and data entry agents will be recruited.

The investigators must be either anthropologists or health personnel who have already conducted focus groups and in-depth interviews in the past (at least one experience in 2 different studies). Data entry agents must be people with experience in collecting data on tablets (at least 2 different studies).

The teams leading the research, including IT, data entry officers, ACF supervisors, RECOS, sociological investigators, will be trained on the research protocol, taking anthropometric measurements, PCIMA, conducting structured interviews, 'assessment of the psychomotor development of participants and the use of tablets. In addition, training on the quality of the intervention, namely the PCIMA, will be organized by the investigation team. During all the trainings, an emphasis will be placed on practice and a standardization test will be organized (especially for the staff of the CS) to ensure the fidelity and accuracy of the anthropometric data taken by the participants.

The investigators will be trained on facilitating focus groups, in-depth interviews and taking notes.

All the teams (of the qualitative and quantitative part) will be trained on the respect and strict observance of this trial protocol.

The data will be secured every evening on a web server via an internet connection and an additional backup will be made on an external hard drive every Friday evening to serve as a backup in the event of a data crash.

c. Study pre-test phase

Before starting, a pre-test phase will be organized to assess the feasibility of the field study. All questionnaires will be tested before the study and back-translated to verify the correct translation of the words and questions, the newsletter and the consent form.

In each of the two health zones selected, two UNTAs not part of the study will be chosen for the pre-test.

The recruitment criteria will be applied as in the study itself. The agents of the selected UNTAs will be employed for the pre-test.

The pre-test will take place over three days and the teams will be made up of three people from the UNTAs selected for the study. In order not to disrupt the proper functioning of the UNTAs, the agents will take turns participating. Per day, in each team, the three agents will come from the three different UNTAs. The remaining two will cover work in their respective UNTAs.

After the pre-test, the data collected will be analyzed in order to assess the feasibility of the study and the mastery of the tools by the agents. After analysis, recommendations will be possible improvements.

d. Roles of the various stakeholders - Governance of the study

Promoter: ACF in DRC

Investigator Principal: Julien Ntaongo, doctoral student at the University of Kinshasa

Co-investigators :

- **ESP Kin**: Teacher. Marie-Claire Muyer and Dr Steve Botomba
- **PRONANUT** : Jean-Baptiste Mayavinga
- **ACF** : the research program manager (Victor NIKIEMA), the Health Nutrition RDD (Marie Pétry), the operational health nutrition technical referent (Sophie Bruneau), the health nutrition research projects referent (Cécile Salpéteur).

Thesis committee:

- **University of Kinshasa** :
  - Child and Juvenile Psychiatry Unit: Professor Samuel MAMPUNZA and Professor Florence MUADI MBIYA;
  - ESP: Professor Marie-Claire MUYER;
  - Pediatrics: Professor Aimée MUPUALA

e. Organization of bodies

The Working Group meets once a week, every Tuesday, to design the study. After the start of the implementation, the meetings will take place once a month for the follow-up, the search for solutions to the encountered challenges, then the cleaning of the data, their analyzes and the writing of the final scientific articles. A Report is produced tracing the exchanges and what is stopped for the protocol.

The Working Group convenes the COPIL for decision-making at the major stages of the research project: 1) validation of the final protocol, including the sub-studies, the budget and the timetable; 2) start of the data collection phase 3) any temporary suspension or modification to protocol or other during the study 4) preliminary results 5) final project report 6) results communication plan.

The WG presents to the COPIL the progress of the project, the decision options and analyzes of these options (consequences, risks, cost), and implements the decisions of the COPIL.

The COPIL or project steering committee is made up of decision-makers from the various project partners:

- **ESP Kinshasa**: Teacher. Marie-Claire Muyer, validating the scientific quality of the project;
- **ACF in DRC**: the Director Benjamin Viennot or his deputy by delegation, validating the mission strategy including operational research, investments in the research project, submission of proposals; the health nutrition RDD validating the technical strategy and the consistency of the research project with it, the resources put into the project (human time);
- **ACF in Paris**: the Regional Director of Operations or her deputy by delegation, validating ACF's humanitarian strategy in general and investments; the head of research at ACF validating scientific partnerships, respect for good research practices and the resources put into the project (human time), the head of the health nutrition sector validating the technical relevance of the research project, its prioritization among 'others in the health nutrition field, the resources put into the project (man time).

The principal investigator

- Write the protocol, design the questionnaires, with the co-investigators
- Trains teams, partners, agents, service providers in the study protocol, its tools, and quality control
- Ensures coordination between everyone and project communication with the support of ACF
- Ensures quality control at the start of the study then during the study, being present on site, sets up the monitoring of data entry, the correction if necessary, the locking up of consents, the protection of data, mutual accountability of all to obtain reliable data
- Organizes coordination meetings in Mbuji Mayi between ACF, the owners of health centers and / or their representatives for the proper implementation of care and concomitant research
- Prepare monthly reports and presentations of study progress to inform study partners and decision makers
- Conducts the final statistical analyzes from the data, writes scientific articles presenting the results obtained with the partners, formulates the conclusions, and suggests operational recommendations for the PCIMA.
- Contributes to the drafting of proposals to secure additional funding
- Presents the final results to the authorities and representatives of the participants, to the international nutrition community and to the scientific community in the field.

The ACF Research Program Manager

- Contributes to the study design, tools, training design
- Coordinates the study on the side of the ACF promoter: brings together the work team to build the study together, convenes the COPIL for decision-making (if temporary suspension, if fundraising, etc.)
- Ensures the possible recruitment of staff for the study
- Train study staff with the principal investigator
- Ensures the coordination between the various actors of the project: researchers, data entry agents, IT, ACF supervisors, UNTI etc. in order to seek the best continuity of care for the children and the smooth running of the study
- Contributes to data quality control, ensures supervision field visits
- Provide reporting on all meetings held by the project team
- Ensure compliance with the schedule of activities by alerting stakeholders
- Keeps the project calendar up-to-date (chronogram), the 1-page flyer for project communication, the Teams group with jointly shared documents
- Contributes to the drafting of proposals to secure additional funding
- Contributes to the writing of final scientific articles as a co-author
- Coordinates the presentation of results to the authorities between researchers and ACF, facilitates the organization of communication events as necessary

ACF supervisors :

- Ensure the first quality control of the data collected in the UNTAs;
- Ensure strict adherence to the SAM treatment protocol in UNTAs;
- Inform the investigators about any anomalies observed in the UNTAs;
- Strengthen the capacities of the UNTA teams which show certain weaknesses;

IT in the UNTA (nurses):

- Participate in research protocol training
- Recruit trial participants according to the inclusion criteria;
- Collect informed consents and keep them locked up before transmitting them to the principal investigator;
- Conduct medical and nutritional consultations with anthropometric measurements;
- Divide the participants into the two groups;
- Distribute RUTF to participants according to their group (control or intervention);

- Report / call by phone to find out how to manage any particular case asking questions about the research protocol, keep a debriefing time at the end of the week to share with researchers the constraints encountered and find solutions together
- Inform of any absences and replacement

Socio-anthropological investigators (assistants and note taker):

- Identify, with help from community relays, focus group participants and in-depth interviews;
- Obtain informed consent from participants in focus groups and in-depth interviews;
- Lead in-depth interviews and focus groups;
- Ensure note-taking during in-depth interviews and focus groups;
- Ensure the recording of in-depth interviews and focus groups with the permission of the participants; transcribe each interview into French in an anonymized Word document with unique identifying number and date, and keep the confidential register of corresponding identifiers and identities up to date
- Transmit to investigators the recordings and transcriptions made and the identification register.

Data entry clerks (ESP):

- Participate in the study protocol training
- Go to the health centers to enter on a tablet all the data of the study - the socio-demographic, economic and anthropometric characteristics of the participants; with respect for data confidentiality, data backup and protection procedures;
- Report any difficulty or question to the researchers to avoid making decisions alone that could affect the quality of the data, not to take initiatives on the data without the researcher's agreement and to note in a logbook all the actions carried out on the data (corrections, modifications etc.)
- Participate in data entry coordination meetings to provide detailed feedback on errors encountered and failure to complete UNTA forms and questionnaires, to health center owners via the researcher and the research PR
- Ensure the transcription of in-depth interviews and focus groups.

f. Organization of the collection

At UNTA level

On admission, if a patient meets the study inclusion criteria, the receiving provider explains how the study is going and requests informed consent to include them in the study. If consent is obtained, the nurse assigns an ID to the child that matches the randomization ID. The randomization group is not mentioned. Information on socio-demographic characteristics is collected by IT.

The provider responsible for medically examining the child and taking the anthropometric measurements examines and provides information on the measurements. Anthropometric measurements will be taken according to the PCIMA protocol once a week when the child visits the center until the cured discharge or up to 12 weeks maximum.

- PB will be measured using a PB tape on the left arm halfway between the acromion and the olecranon;
- The weight using a SECA scale depending on whether or not the child can stand up;
- The height will be measured using a wooden measuring board in the supine or upright position depending on whether the child is <24 months or ≥24 months (or ≥ 87 cm) respectively;
- Edemas will be assessed according to their importance (0; + if limited to the feet; and ++ if limited to the legs).

The medical treatment is given to the child according to what is defined in the PCIMA protocol.

Finally, the child receives the amount of RUTF planned for the week as well as health education. It is the same provider in charge of the admission who will ensure the distribution of RUTF and will know who receives the reduced dose according to the IDs of the children. This provider will not follow up on children to keep blind. Each participant receives the quantity of RUTF described in Table 1 (presented above).

All the information on the identifier, the socio-demographic characteristics and the anthropometric measurements are noted in the individual follow-up sheet of the corresponding participant.

<u>Assessment of psychomotor development</u>: Depending on the sampling interval, the evaluation of psychomotor development will be carried out on 40% of the sample size, by means of the ASQ 3 score and will include two parts: the execution of the tasks by the child and the filling

of the questionnaire by the guides. For each child, the performance will only last between 10 to 15 minutes. The results of the assessment of psychomotor development are listed in the corresponding item.

The practical modalities of the DPM evaluation as well as the corresponding items are given in the appendix to this protocol (appendix 3).

For the children who will be selected for the Sub-study on psychomotor development, their anthropometric measurements (weight, height, MUAC, search for edema) will be taken during a visit after treatment when they respond to the appointments for the evaluation of the patient. psychomotor development: at the end of treatment (for children declared cured) then at six months. This information will be noted in their corresponding individual monitoring sheet.

In the community

These are mainly qualitative surveys to assess the degree of acceptance of the reduced dose of RUTF by the community. To achieve this, FGs, in-depth interviews and field observations will be carried out:

- ***Focus groups***: they will bring together an average of 8 people and will last between 45 minutes to an hour. Participants in FG will be accompanying children participating in the study. The drawing of lots will be carried out among those accompanying the last children followed. In each ZS, 3 focus groups will be carried out, one with the mothers, one with the fathers and one with the young people.

- ***In-depth interviews***: will mainly concern key informants including politico-administrative authorities, traditional authorities (village chief) healthcare providers (nurses from UNTAs), the two head doctors of ZS, healthcare providers (due to a nurse by UNTA selected) and community leaders (depending on their influence, 3 community leaders will participate per ZS).

- ***Field observations***: they will be carried out in the UNTAs, households of participants, markets and other strategic places of the corresponding ZS.

Triangulation of sources and methods will be used to improve data quality.

At the end of the FG, in-depth interviews and field observation, the reports of the day's progress, the notes taken as well as the recordings will be sent to the investigators.

The practical modalities and the tools to be used are given in the appendix to this protocol (appendix 4).

2.3.2. Data managment

The data will be collected on individual UNTA files by the IT then by double entry for 10% of the files, they will be recorded on tablets by the data entry officers recruited for the study. The principal investigator will regularly analyze the data collected after it has been sent to the central server and will provide feedback to the data collectors on the inconsistencies observed.

The principal investigator, the data entry agents under the supervision of the scientific manager, will have to check the quality of the data on a daily basis and provide feedback to the ACF supervisors and IT via the Research Program Manager for any corrections on the site.

Only the principal investigator and the Research Program Manager will have free access to the data during the study. The latter two are responsible for extracting the database from the server, making it anonymous and sharing it for statistical analysis.

2.3.3. Statistical analyzes

For the quantitative part of the study, the data will be summarized in Mean and Standard Deviation if they are normally distributed (normality test) for continuous variables and in absolute and relative frequencies for categorical variables.

The t-test and the chi-square test will be used respectively to compare the means and the proportions of the socio-demographic characteristics at admission for the quantitative and qualitative variables.

Linear regression analyzes will be used to determine the significant differences between the 2 groups of children for the quantitative variables.

Binary logistic regression analyzes will be used to determine the significant differences between the 2 groups of children for the binomial qualitative variables (2 response modalities).

Multinomial logistic regression analyzes will be used to determine the significant differences between the 2 groups of children for the multinomial qualitative variables (3 or more modalities).

All analyzes will be done in an adjusted and unadjusted model and a difference is statistically significant when the P-value is less than 0.05.

The results will be analyzed using statistical software, in this case STATA, version 17.

The main analyzes will be done by intention to treat (ITT) and per protocol for the main result, in this case, the speed of weight gain. The other secondary results will be analyzed only by intention to treat (ITT).

For the DPM, a score will be assigned to the participant and the sum will constitute the total score for each participant per domain.

For focus group data, after transcription of the recordings into Word 2019, thematic analysis will be done using Excel tables (matrix) or Nvivo® software.

2.4. Monitoring

2.4.1. Semi-internal security analysis

Composition : Data monitoring will be ensured by a researcher specializing in pediatric nutrition and a statistician who will be independent of the investigation team.

Role: Evaluate the progress of the study (safety of participants, proper conduct of the study, efficacy of the reduced dose). Once a month, the semi-internal security analysis committee will have access to the data for analysis in order to follow the progress of each participant. The strict application of the protocol, the other parameters including the undesirable and unexpected effects will also be regularly evaluated.

Report : the semi-internal safety analysis committee will prepare a summary report on the progress of the participants and the progress of the trial as well as the main recommendation to continue the study, or modify the protocol, or discontinue the study.

The practical modalities of the functioning of the semi-internal data security analysis committee will be detailed during the meeting sessions with its members before the start of the study.

2.4.2. Prejudices

The RUTF used for this study does not present any danger for the participants unless some are allergic to one of its ingredients (peanut paste, powdered milk for example). Children whose allergy is known before the start of the study will not be eligible for the study and treated outside the 2 groups.

Due to the reduction in the dose of RUTF, rigorous monitoring will be implemented in order to follow the clinical progress and the weight of the participants in the study.

At each visit, questions will be asked of participants and accompanying persons in order to identify potential unwanted or unexpected effects. If these effects are reported, the information is passed directly to the investigator and field partners for immediate corrective action.

III. Ethical considerations and publications

3.1. Authorizations

To start you will need certain permissions, namely:

- From the PRONANUT validation committee
- From the ESPK Ethics Committee
- The Governor of Kasai Oriental and the Head of Territory
- Notify MCZS in two zones

3.2. Authorization from the research ethics committee

Before starting the study, the overall protocol, questionnaires and all appendices, including the informed consent form, will be submitted to the ethics committee for its opinion.

3.3. Protocol amendments

If for one reason or another it was essential to make changes to the trial protocol, a dated register detailing the various changes to be made to the trial protocol will be put in place.

These modifications will only be effective once the ethics committee has validated them.

Participants will be notified of changes to the protocol and will be required to provide additional consent for the new changes to be applied to them.

3.4. Free and informed consent or assent

On admission, the provider should obtain the informed consent of each patient before including them in the study. He will make it clear to caregivers that they are free to choose whether or not their child participates in the study and that they can decide to stop participating at any time without fear of repercussions on their child's right to treatment.

All patients who do not meet the inclusion criteria and those who refuse to participate in the study will be treated according to the national PCIMA protocol.

When a patient decides to no longer participate in the study, at any time, he will be automatically treated according to the protocol of the DRC PCIMA.

The informed consent form is annexed to this protocol (annex 2).

3.5. Confidentiality

Throughout the study (data collection, data analysis, participant files, etc.) confidentiality and anonymity on the identity of participants will be kept in the files. Once the data has been collected, they will be stored on a server where only the investigator and the ACF Research Program Manager will have access. The latter two are responsible for cleaning the database to make it anonymous according to established procedures.

Special authorization from the participant or his tutor will be required before using a photo or other identifying element as shown in the attached form (Annex 1).

3.6. Declaration of interests

The investigators reassured that they had no conflict of interest in carrying out this study despite the fact that the principal investigator was also a technical expert in nutrition with PRONANUT, an organ of the Ministry of Health dedicated to nutrition. The participation of donors is excluded. The participation of the study sponsor (ACF) in the study is strong due to its dual role of co-investigator as well as sponsor.

3.7. Data accessibility

Access to the data will be protected by a code that will only be known to the principal investigator and the research program manager. Only these two people will have access to the raw data throughout the study period. It is up to them to make the databases anonymous before analysis. The final databases of the study (raw and cleaned) will be archived in the DRC by the ESP and in France by ACF and may be made available to researchers by making a written request to the body chosen according to the terms of data access request for each instance.

3.8. Ancillary and post-test care

During the study, all participants will benefit from systematic medical care as set out in the PCIMA protocol in force in the DRC.

Up to three months after the recovery of the last participant, close follow-up will be carried out and in the event of a relapse or other medical complications due to the trial, the participants will be taken care of immediately.

3.9. Publication policy

During the study, preliminary results will be presented to the members of the steering and monitoring committee.

At the end of the study, feedback workshops will be organized to inform local authorities and participants of the conclusions of the study in which they participated. The main results of this study will be published in the form of scientific articles and in peer-reviewed journals in Open Access in order to ensure universal access to the results. These results will also be presented in the form of a doctoral thesis with the University of Kinshasa in the DRC.

Once the articles are published, the actors involved in nutrition will be informed through the usual channels including CMAM Forum, EN-Net and Field Exchange. Participation in international conferences on nutrition will also help to publicize the findings of this study.

The results of this study constitute only guidelines and do not imply a modification of the national protocol of the PCIMA which remains the domain of the Ministry of Health in collaboration with its partners.

IV. Project risk analysis and mitigation measures

RUTF supply disruption

This is the main risk involved in providing adequate treatment for SAM children.

RUTF is indeed managed by UNICEF and the government, receiving funds to purchase and deliver RUTF. These funds are not always sufficient or guaranteed in the event of an unexpected increase in malnutrition cases.

RUTF comes from other countries where there is a manufacturing plant so it incurs an export <> import process and sometimes remains blocked in customs for long months.

It must then be transported and stored throughout the chain.

The main source of RUTF for this project is national through the usual circuit serving the health centers.

ACF has a stock of 3 months at the end of May 2021 for the nutritional program underlying the research but thereafter the supply depends on the central level.

Mitigation measures: ACF will closely monitor RUTF consumption by the program and research and for the two Health Zones concerned will strongly support in the capital as well as in the Kasai region the competent authorities to facilitate the supply and avoid any disruption.
.

Loss of access to health centers

The risks are low but exist due to flooding, conflict, or degraded roads.

Mitigation measures : we will equip, for the duration of the study, the ICP in charge of finding a telephone for remote monitoring and data sharing, we will ensure sufficient stocks of RUTF and amoxicillin and anti-malarial (3 weeks in advance) for all the health centers concerned.

Medical staff strike

The risk exists but experience has shown that it does not take much time and the Government is currently in talks with the union bench.

Mitigation measure: the ACF project currently underway provides financial support for the operation of FOSAs and in terms of incentive premiums for providers of two ZSs concerned by the study. In this context, the staff is less inclined to follow the strike movement as is currently seen. Otherwise, we arrange to have the health centers operate at least two in the morning to serve the children. Community distribution of RUTF can also be used.

Incomplete funding of the project at start-up

The lack of funds for the entire project presents an ethical risk of not being able to process the research data collected in year 1 until effective publication.

Mitigation measure : ACF with the ESP and the Pronanut will conduct the search for funding to cover the analyzes and publications of the results of the study.

V. Budget

Funding for the study will be provided by the partners listed in Table 7:

Chart 8 : Sources of funding for the study at the date of the protocol

Donors	Duration	Rising	
FCDO	January - December 2021	117,000 USD	Phase 1
FCDO	2022 - 2023	In the process of assembly	Phase 2 and 3
Other funding	2022 -2023	In the process of assembly	Phase 2 and 3

VI. Chronogram of activities

Activities	2021 (month)											2022 (month)												2023 (month)										
	2	3	4	5	6	7	8	9	10	11	12	1	2	3	4	5	6	7	8	9	10	11	12	1	2	3	4	5	6	7	8	9	10	11
**Programmatic activities**																																		
Recruitment and training of field teams								X																										
Launch of purchases								X																										
Project communication																																		
Meeting with local partners											X																							
Pilot phase - feasibility study																																		
**Implementation of the project in the field**																																		
Main study: Inclusion of children										X																								
Main study: Treatment of children included in the study (max 4 months after inclusion)													X																					
Main study: Follow-up of children after recovery (Up to 3 months)																X																		
Assessment of psychomotor development																			X															
Assessment of the acceptability of the reduced dose										X																								
Economic evaluation data collection													X																					
**Research activities**																																		
Submission to the validation committee (Min. Health)					X																													
Submission to the ethics committee						X																												
Data quality control																			X															
Data cleaning																					X													
Data analysis																							X											
Writing and submitting an article on the velocity of weight gain																					X													
Writing and submitting an article on the acceptability of the reduced dose of RUTF																								X										
Writing and submitting an article on psychomotor development																								X										
Writing and submission of article on economic evaluation																											X							
Thesis manuscript writing																															X			
Thesis defense																																X		
Drafting of the final project report																																		

VII. Bibliographical references

1. Unicef, WHO, World Bank Group. Joint malnutrition estimates, 2020 [Internet]. Available at: https://www.who.int/publications/i/item/jme-2020- edition
2. UNICEF. The State of the World 2019: Children, Food and Nutrition. Grow well in a changing world. Executive summary. New York: Unicef; 2019 Oct p. 24.
3. Headey D. Impacts of COVID-19 on childhood malnutrition and nutrition-related mortality. The Lancet. 2020; 396: 3.
4. Unicef. An estimated 10.4 million children in the Democratic Republic of Congo, northeastern Nigeria, the central Sahel, South Sudan and Yemen will suffer from acute malnutrition in 2021 [Internet]. [cited 21 Feb 2021]. Available at: https://www.unicef.org/drcongo/communiques-presse/enfants-malnutrition-aigue-2021
5. Laborde D, Martin W, Vos R. Poverty and food insecurity could grow dramatically as COVID-19 spreads. In 2020. p. 16-20.
6. Roberton T, Carter ED, Chou VB, Stegmuller AR, Jackson BD, Tam Y, et al. Early estimates of the indirect effects of the COVID-19 pandemic on maternal and child mortality in low-income and middle-income countries: a modeling study. Lancet Glob Health. 2020 Jul; 8 (7): e901-8.
7. COVID-19 will double number of people facing food crises unless swift action is taken | World Food Program [Internet]. [cited 15 Apr 2021]. Available at: https://www.wfp.org/news/covid-19-will-double-number-people-facing-food-crises-unless-swift-action-taken
8. Barden-O'Fallon J, Barry MA, Brodish P, Hazerjian J. Rapid Assessment of Ebola-Related Implications for Reproductive, Maternal, Newborn and Child Health Service Delivery and Utilization in Guinea. PLOS Curr Outbreaks [Internet]. 2015 Aug 4 [cited Apr 15, 2021]; Available at: index.html% 3Fp = 61210.html
9. Fore HH, Dongyu Q, Beasley DM, Ghebreyesus TA. Child malnutrition and COVID-19: the time to act is now. Lancet Lond Engl. 2020; 396 (10250): 517-8.
10. Garza C, Onis M. Relationale for developing a new international growth reference. Food and Nutrition Bulletin. 2004; 25 (1).
11. WHO. WHO child growth standards and the identification of severe acute malnutrition in infants and children. A Joint Statement. Geneva: WHO; 2009.
12. Ministry of Health / DRC. National Protocol for the Management of Acute Malnutrition. Kinshasa, Democratic Republic of Congo: Ministry of Health / DRC; 2016 p. 227.
13. Briend A, Lacsala R, Prudhon C, Mounier B, Grellety Y, Golden MH. Ready-to-use therapeutic food for treatment of marasmus. Lancet Lond Engl. 1999 May 22; 353 (9166): 1767-8.
14. WHO, PAM, UNSCN, Unicef. Community management of severe acute malnutrition. Joint statement by the World Health Organization, the World Food Program, the United Nations System Standing Committee on Nutrition and the United Nations Children's Fund [Internet]. 2007 [cited 19 Apr 2021]. Available at: https://www.who.int/nutrition/publications/severemalnutrition/978-92-806-4148-6_eng.pdf?ua=1
15. WHO, editor. The management of severe malnutrition: manual for use by doctors and other health personnel in managerial positions. Geneva; 2000. 63 p.

16. Collins S. Treating severe acute malnutrition seriously. Arch Dis Child. 2007 May; 92 (5): 453-61.
17. Subramaniyan M, Skoogh A, Salomonsson H, Bangalore P, Gopalakrishnan M, Muhammad AS. Data-driven algorithm for throughput bottleneck analysis of production systems. Prod Manuf Res. 2018 Jan 1; 6 (1): 225-46.
18. Chloe P, Samuel Hauestein S, Guerrero ES. Access for All Volume 2: What factors influence access to community-based treatment of severe acute malnutrition? [Internet]. London, United Kingdom; 2013 [cited 19 Apr 2021] p. 24. (Coverage Monitoring Network). Available at: https://www.coverage-monitoring.org/wp-content/uploads/2013/12/AAH-Policy-Paper2-06-12-13-updated.pdf
19. Ministry of Health / DRC. National Health Development Plan 2016-2020. Towards Universal Health Coverage. Kinshasa, Democratic Republic of Congo; 2016.
20. Dolan C, Khara T, Shoham AMA and J. Government experiences of scale-up of Community-based Management of Acute Malnutrition (CMAM): A synthesis of lessons (2012). 1 Feb 2012 [cited 19 Apr 2021]; Available at: www.ennonline.net/cmamgovernmentlessons
21. Sphere Project, editor. The sphere handbook: humanitarian charter and minimum standards in humanitarian response. Fourth edition. Geneva, Switzerland: Sphere Association; 2018. 406 p.
22. James PT, Van den Briel N, Rozet A, Israel A, Fenn B, Navarro - Colorado C. Low - dose RUTF protocol and improved service delivery lead to good program outcomes in the treatment of uncomplicated SAM: a program report from Myanmar. Matern Child Nutr. 2015 Apr 7; 11 (4): 859-69.
23. Ciliberto MA, Sandige H, Ndekha MJ, Ashorn P, Briend A, Ciliberto HM, et al. Comparison of home-based therapy with ready-to-use therapeutic food with standard therapy in the treatment of malnourished Malawian children: a controlled, clinical effectiveness trial. Am J Clin Nutr. 2005 Apr; 81 (4): 864-70.
24. Kangas ST, Salpéteur C, Nikièma V, Talley L, Ritz C, Friis H, et al. Impact of reduced dose of ready-to-use therapeutic foods in children with uncomplicated severe acute malnutrition: A randomized non-inferiority trial in Burkina Faso. Persson LÅ, editor. PLOS Med. 2019 Aug 27; 16 (8): e1002887.
25. Kangas ST, Kaestel P, Salpéteur C, Nikièma V, Talley L, Briend A, et al. Body composition during outpatient treatment of severe acute malnutrition: Results from a randomized trial testing different doses of ready-to-use therapeutic foods. Clin Nutr. 2020 Nov; 39 (11): 3426-33.
26. Nikièma V, Kangas ST, Salpéteur C, Ouédraogo A, Lachat C, Bassolé NHI, et al. Adequacy of Nutrient Intakes of Severely and Acutely Malnourished Children Treated with Different Doses of Ready-To-Use Therapeutic Food in Burkina Faso. J Nutr. 11 Feb 2021; nxaa393.
27. Lagrange MA. Disorder as the art of governing. The Kamwina Nsapu rebellion, symbol of Congolese evil. French Institute of International Relations, IFRI; 2017 Sep p. 24.
28. Rolley S. DRC: Violence in KasaÏ - Kamuina Nsapu: the death of a chief (1/3) [Internet]. RFI. 2017 [cited 24 Feb 2021]. Available at: http://webdoc.rfi.fr/rdc-kasai-violences-crimes-kamuina-nsapu/chap-01/
29. FIDH / ASADHO / LE / LOTUS Group - DRC. Massacres in Kasai: crimes against humanity in the service of organized chaos [Internet]. Kinshasa, Democratic Republic of Congo; 2017 Dec [cited 24 Feb 2021]. Available at:

https://reliefweb.int/sites/reliefweb.int/files/resources/rdc_704f_18_dec_2017_ultra-ultralight.pdf

30. Interpeace. Challenges and priorities in Kasai and Kasaï Central [Internet]. Interpeace; 2020 Oct [cited 24 Feb 2021] p. 16. Available at: https://www.interpeace.org/wp-content/uploads/2020/10/2020-Defis-et-priorites-Kasai-Report-.pdf
31. Food Security / DRC cluster. Food security threatened in the Kasaïs zone [Internet]. https://fscluster.org/democratic-republic-congo. 2017 [cited 24 Feb 2021]. Available at: https://fscluster.org/sites/default/files/documents/alerte_kasai.pdf
32. IPC. IPC Analysis of Acute Food Security. Kinshasa, Democratic Republic of Congo: Integrated Food Security Classification Framework / DRC; 2020 Sep
33. PRONANUT. Nutritional Surveillance, Food Security and Early Warning (SNSAP) [Internet]. Kinshasa, Democratic Republic of Congo: Ministries of Health / DRC; 2020 Oct [cited 23 Feb 2021] p. 24. Report No .: 41. Available at: https://reliefweb.int/sites/reliefweb.int/files/resources/bulletin_snsap_ndeg41_31_oct_2020_df.pdf
34. Shevell M, Ashwal S, Donley D, Flint J, Gingold M, Hirtz D, et al. CME Practice parameter: Evaluation of the child with global developmental delay. American Academy of Neurology. 2003 Feb 11; 60 (3): 367-80.
35. Larson LM, Yousafzai AK. A meta-analysis of nutrition interventions on mental development of children under-two in low- and middle-income countries. Matern Child Nutr. 2017; 13 (1): e12229.
36. Gorman KS. Malnutrition and cognitive development: evidence from experimental / quasi-experimental studies among the mild-to-moderately malnourished. J Nutr. 1995; 125 (8 Suppl): 2239S-2244S.
37. Freeman HE, Klein RE, Townsend JW, Lechtig A. Nutrition and cognitive development among rural Guatemalan children. Am J Public Health. 1980 Dec; 70 (12): 1277-85.
38. Herr M, Descatha A, Aegerter P. Non-inferiority and equivalence trials: key points of their methodology. Rev Internal Medicine. 2018 May; 39 (5): 352-9.
39. Julious SA, Campbell MJ. Tutorial in biostatistics: sample sizes for parallel group clinical trials with binary data. Stat Med. 2012 Oct 30; 31 (24): 2904-36.
40. Julious SA. Sample sizes for clinical trials with normal data. Stat Med. 2004 Jun 30; 23 (12): 1921-86.
41. Donner A, Klar N. Design and Analysis of Cluster Randomization Trials in Health Research [Internet]. Great Britean. 2000 [cited 26 Feb 2021]. 179 p. Available at: https://www.wiley.com/en-us/Design+and+Analysis+of+Cluster+Randomization+Trials+in+Health+Research-p-9780470711002
42. Morillon Y. Randomization theory. : 17.

VIII. Appendices

Annex 1: Information for participants

Principal investigator:
Julien NTAONGO ALENDI
Phone. : + 243 8 26 08 33 59
E-mail : j.ntaongo@gmail.com

INFORMATIVE MAIL

Project title

Efficacy of a reduced ration of RUTF in the treatment of severe acute malnutrition with uncomplicated edema (+, ++) in children 6 to 59 months
Before deciding if you are going to participate in this research, it is important that you understand why we are doing this research and what it will involve. Take the time to read this letter carefully and talk about it if you wish. Do not hesitate to ask us questions to clarify a point or to have more information. Take your time to decide if you want to participate. Please read on.

Why was I asked to participate?

We ask you if you and your child are willing to participate in this research because your child is between 6 and 59 months old, has been diagnosed with severe acute malnutrition without medical complications and is entitled to nutritional treatment at enriched peanut butter base that can be taken at home.

What is the aim of the research?

The object of this study is to find out whether the child could be cured with a smaller amount of fortified peanut paste - this is called ready-to-use therapeutic food (RUTF). In fact, by giving just enough to heal the child, we could save a little RUTF which would be used to treat other malnourished children. We have already tried to treat children with a little less product in Burkina Faso and we have found that they heal as much and the same as children who receive the standard dose. Here we are going to make two groups and draw lots for the child who receives the reduced dose and the one who receives the normal dose. As with medicines, it is not helpful to give more than the correct dose to cure a disease. If the treatment is well followed,

Can I choose whether my child will receive a standard or reduced ration?

It is necessary that the participants cannot choose for themselves which group to go to. The distribution will therefore be drawn at random. Either way, the healing process is followed closely for all children and if it turns out that yours is not gaining enough weight, he or she will be taken out of our study to return to treatment. usual with a standard dose at the same health center.

If I agree to participate, what will happen to me and my child?

If you agree on behalf of your child to participate in this research, you will be asked a few questions about your child, yourself and your family.
Your child's appetite will be tested by observing how he reacts to a small amount of RUTF. If your child enjoys RUTF and manages to take a dose, he will be assigned to one of two groups if you give your free and voluntary consent: standard ration or reduced ration. Groups will be drawn. You will therefore not be able to choose in which group your child will be. Your child will receive all the care they need since there is no difference in care between the two groups, only the amount of RUTF given changes a little.
You will be asked to follow the treatment every day and come back one set day per week for your child's follow-up medical consultation and to receive therapeutic foods, as always for the treatment of malnutrition.
Each week, when you return to the health center, your child will be seen by the owner of the health center. He or she will weigh him or her and measure his or her waist and arm circumference, and make sure that there is no edema. He / she will also ask you questions about your child's state of health and perform a full examination for possible illnesses.

Once your child has recovered from malnutrition and leaves the program, you will be asked to come back to the health center once every 2 weeks for 3 months. At each visit, the nurse will weigh your child and measure his height, his arm circumference and look for any edema. On this occasion, you will be asked questions about your child's state of health and the other foods he eats. If your child is absent, a RECO can contact you by phone or visit you at home to find out the reasons for this absence.

Are there any benefits or risks of participating?

The main advantage of your participation in the study is that your child will be treated for malnutrition and will receive the same care as that usually provided by the health center.
There are no other direct advantages to this participation, other than that your child will be followed a little more closely by the research team compared to regular treatment.
We hope that with your participation and that of your child, we will be able to develop such an effective and less expensive treatment for malnutrition that could ultimately benefit all children with SAM.
If your child is not gaining enough weight or losing weight, we will check your child for other illnesses and make sure they get the right treatment if needed. We'll also see how much RUTF he actually eats. We will advise you on the best way to make sure your child is gaining enough weight to heal. If they are not really gaining weight, we will make sure that your child is examined more closely by the medical team to understand why. If your child has symptoms such as fever, vomiting, diarrhea, etc., he will be referred to the hospital for treatment according to normal protocol.
The study is covered by insurance and the principal investigator will ensure the treatment and compensation for any adverse effects observed in the child following this study, effects related to the use of the nutritional product and / or the procedures for research, over the duration of the study.

Is my participation in the research confidential?

All information collected about you, your child and your family during this research will be treated with the strictest confidentiality. Prior to our analysis of the data, your name and contact details will be deleted from all information media concerning you, with the exception of the consent form. These forms will be kept locked away at the researcher's office. We will only take pictures of you and your child with your consent and will only use them for training and communication purposes about the study, if you allow us.

Do I have to participate?

No, you are not required to participate in this study. Even if you have agreed to participate, you are free to stop participating at any time, without having to justify yourself.
If you agree to participate, you will be given this informative mail which you will need to keep and you will be asked to sign a consent form. Once again, you can withdraw from the program at any time, without any justification on your part, and your child will be treated at the health center like any malnourished child according to ordinary protocol.
If you refuse from the start not to participate in the study or if you refuse to continue participating along the way, there will be no consequences for the child: your child will have the treatment at the health center as the other children according to the ordinary protocol. Your child's treatment and care will not be interrupted even if you decide to stop the study at some point.

Has this study been officially approved?

Yes, the study is approved on several levels. The nutrition studies validation committee bringing together more players around the national nutrition program validated the study protocol. The protocol was submitted to the National Health Research Ethics Committee reviewed and approved the study. This study is funded by FCDO through Action Contre la Faim.

Who to contact in case of a question or complaint?

For any complaint or question, you can contact a member of the health center team. You can also contact the study manager whose contact details are here:

***Julien NTAONGO* ALENDI**

Phone. : + 243 8 26 08 33 59
E-mail : j.ntaongo@gmail.com
Don't hesitate to ask questions, big or small. We will do our best to answer them.
Do you agree to participate in the study?
thank you very much

Annex 2: Informed consent form

	Yes	No
I confirm that I have read and understood the informative letter concerning the aforementioned study, and have had the opportunity to ask questions	☐	☐
I understand that my participation is voluntary and that I am free to withdraw from the research at any time, without having to justify myself, and that such a decision will in no way affect my child's rights to the treatment.	☐	☐
I understand that our participation in the study does not entitle me and my child to any specific treatment, compensation or gift	☐	☐
I agree, on behalf of my child or dependent child, to participate in the study	☐	☐
I agree that my data will be kept anonymously for 15 years on protected servers to be used for further scientific research to improve the health of children.	☐	☐
I agree that my data may be aggregated with that of other people for the purposes of subsequent scientific research to improve the health of children.	☐	☐

IMAGE RIGHT

I agree that any photos or videos in which I and / or my child will appear will be used anonymously for training and presentation purposes at conferences.	☐	☐

NAME AND SIGNATURE OF PARTICIPANT

Unique identifier of the MAS child enrolled: /…/…/…/…/.../ (5 digits)

Name of the child: ……………………………………………………………………………

Name of parent or legal guardian : ………………………………………………………………
..

Date: /…../……./…………/ (DDMMYYYY) Signature or fingerprint: ……………

NAME AND RESEARCH AGENT

Name of research officer: …………………………………………………………………

Date: /…../……./…………/ (DDMMYYYY) Signature or fingerprint: ………………….

Annex 3: Assessment of psychomotor development

1. Context and rationale

Psychomotor development (DPM) is the set of evolutionary processes of acquisition by an individual, from birth to adulthood, skills in the field of motor skills, cognitive, language and social interactions (1). We talk about psychomotor developmental delay (RDPM) when disorders interfere with this process. There is no universally accepted definition of RDPM. For the WHO, this is “Specific Mixed Developmental Disorders” without one of the components being predominant to be considered as the main diagnosis.(2). For France, these are disorders related to the standardized reference ages(3). More recently, a group of experts proposed the term "Early developmental disability (onset before the age of 5)" to refer to RDPM.(4). For this work, it is the American definition that will be considered, it is a delay of less than or equal to -2 SD below the average in at least two of the four areas of psychomotricity or an Intellectual Quotient (IQ) is less than 70(5).

Epidemiologically, more than 200 million children have psychomotor development disorders. Asia and Sub-Saharan Africa would concentrate the greatest number of these children(6). In the DRC, global statistics are not available but a recent study conducted in 2 provinces found a prevalence of 4.1 and 4.9 respectively for severe mental and motor retardation.(7).

The etiology of RDPM is multifactorial ranging from hereditary, familial and community factors (8). Malnutrition and particularly micronutrient deficiencies are associated with delayed child development leading later to poor school performance for children who are victims.(9) (10) (11).

The effects of malnutrition on psychomotor development depend on the stage and nature of the deficiency concerned. (12). Malnutrition is responsible for the functional isolation that causes RDPM by reducing social interactions, exploratory behavior, attention, motivation and a low level of activity(13) (14) (15). Functional isolation and its consequences will be all the more serious if it is the mother-child relationship (or the mothering environment, if applicable) that is disrupted.(17) (18). In addition, severe acute malnutrition has been shown to cause permanent anatomical and biochemical changes affecting brain development. Children with SAM reportedly have reduced brain size and EEG abnormalities(19) (20). More recently, researchers have shown that children with SAM have more pronounced RDPM in certain areas.(21). The consequences of SAM in early childhood would be felt for very long periods, sometimes throughout life, rendering victims incapable of being independent in

adulthood.(22). This RDPM would be reduced if children benefit from good nutritional care(23). If the care is provided correctly, the beneficial effects on psychomotor development are felt very quickly: on motor development in the 24 months following the treatment. The development quotient would improve from the second year(24) (25).

Often malnutrition occurs in a context of poverty where parents are unable to provide children with all the care and conditions necessary for their development in terms of DPM. To be reassured that the observed RDPM is due to malnutrition, it is necessary to go through the randomization of the participants. In addition, it is necessary to study together the other associated factors, including the psycho-emotional environment from which the child with SAM evolves.

This is the reason why this study is proposed in order to determine the prevalence of RDPM in SAM children and to assess their evolution after treatment in the ZS of Nzaba and Bonzola in the province of Kasai Oriental in the DRC.

2. Goals

at. Main objective

Contribute to improving the overall care of children with SAM without medical complications by integrating aspects of psychomotor development.

b. Specific objectives

The specific objectives of this study are:

- To determine the prevalence of RDPM in SAM children without medical complications in the ZS of Nzaba and Bonzola in the province of Kasai Oriental in the DRC;
- Evaluate the evolution of psychomotor development in SAM children without medical complications in the ZS of Nzaba and Bonzola in the province of Kasai Oriental in the DRC;

To compare psychomotor development in SAM children without medical complications as a function of the dose of RUTF received in the ZS of Nzaba and Bonzola in the province of Kasai Oriental in the DRC.

3. Methodology

3.1.Participants, interventions and outcomes

3.1.1. Study framework

The present work is a sub-study of a large study evaluating the effectiveness of the reduced dose of RUTF in the management of SAM without medical complications. The study will take place in the ZS of Nzaba and Bonzola in the province of Kasai Oriental in the DRC. This province experienced a great conflict between the militia of Kamwina Nsapu and the regular army of the DRC. The consequences of this conflict had very serious influences on the nutritional situation of children under 5 in the province, more than 30 and 33% of them would suffer from malnutrition respectively in the ZS of Nzaba and Bonzola. Knowing the consequences of both an unfavorable psychoaffective environment and malnutrition on psychomotor development, this is why this study in these ZS.

3.1.2. Participants

b. Inclusion criteria

To participate in this study, individuals must express additional consent to participate outside of the inclusion criteria of the overall study on the evaluation of the effectiveness of the reduced dose of RUTF in the management of SAM. without medical complications:

- Children aged 6 to 59 months;
- MAS (PT <-3 Z-score and / or PB <115mm and / or bilateral edema (+, ++);
- Without medical complications;
- Living in the selected health zone.

c. Non-inclusion criteria

- Congenital malformations which may affect psychomotor development;
- History of neurological pathologies with impact on psychomotor development;
- Any child of drug addicted mothers.

3.1.3. Expected results

- **Prevalence of psychomotor developmental delay**: assess the skills and abilities acquired in each of the 4 domains of DPM (Motor skills, language, cognition and sociability);
- **Evolution of psychomotor development according to the dose of RUTF received**: compare the improvement in the acquisition of skills and abilities acquired in each of 4 areas of DPM according to the dose of RUTF received according to the trial protocol.

3.1.4. Sample size

$$n = t^2 \times p \times (1-p) / m^2$$

- *n: Minimum sample size to obtain significant results for an event and a fixed level of risk*
- *t: Confidence level (the typical value of the 95% confidence level will be 1.96)*
- *p: estimated proportion of the population exhibiting the characteristic studied (0.50 is considered if p is unknown);*
- *m: Margin of error (generally set at 5%).*

Applying the above formula, the minimum sample for this study is 384 children. After rounding up, a total of 400 children will be included in the study on psychomotor development, including 200 in the intervention group (reduced dose) and 200 in the control group (standard dose).

3.1.5. Sampling technique

This is a systematic study with a survey step of 2. In practice, after the first child, it is the 3rd child who will be the next participant according to the inclusion criteria until reaching the necessary 400. for this study.

3.2. Recruitment

Patients will be recruited at the UNTA level whether they come directly from the community or whether they are referred from the CPS.

The participants will be distributed among the different UNTAs in proportion to the demographic weight.

On admission, anthropometric measurements including weight, height, MUAC, head circumference and nutritional edema will be taken on all children. To be recruited, each participant must have one must meet the inclusion criteria of the under study. Individuals with

SAM but who do not meet the criteria for the sub-study will be included in the parent study or managed according to the PCIMA protocol but will not be part of the sample for this study if applicable.

3.3. Data collection and analysis

3.3.1. Data collection methods

a. Data collection technique

Data collection will consist of taking the psychomotor development assessment test. For the present study, it is the test “Age, Stage and Questionnaires 3rd edition”, ASQ3 which will be used.

Taking the test

The test will take place in two stages:

- Observation of the realization of the activities by the child. These are 5 different items relating to:
  - Communication (language);
  - Motor skills (fine and gross);
  - Problem solving (cognition);
  - Individual or social skills (sociability).
- Complement of the test with the completion of a questionnaire of 30 items by the mother or the accompanying person.

Duration and pace of the assessment

For each participant, the test lasts between 10 to 15 minutes maximum. Each child will undergo three assessments, one on admission, one at discharge in the third month (exit from treatment for children declared cured) and one in the sixth month.

Data logging

The activities carried out by each participant are recorded in a summary results sheet allowing rapid interpretation.

Questionnaires are managed and corrected using the ASQ Web-Based® Screening software available online.

b. Data collection plan

Staff recruitment and training

The study must be organized in conditions as close as possible to road practice. As a result, the providers assigned to taking anthropometric measurements will also be responsible for evaluating the psychomotor development of each participant.

These providers will be trained by the psychomotor therapist in order to become familiar with the use of ASQ3 and the assessment of the psychomotor development of the participants.

c. Organization of the collection

Depending on the sampling interval, when a child meets the inclusion criteria, immediately after taking anthropometric measurements, providers assess their psychomotor development.

The assessment will be done on admission, after recovery and then once every six months until the end of the study. Even if they are declared cured and they no longer come to UNTA, providers will keep contact details on their addresses in order to continue DPM assessments.

3.3.2. Data analysis

Descriptive analysis (percentage, medians with interquartile space and extremes, means plus or minus standard deviation) will be performed for categorical data.

The chi-square test will be used for the comparison of proportions and the Student's t-test to compare the means in the two groups.

4. Bibliographical references

1. Brunet O, Lézine I. Scale of psychomotor development of early childhood. EAP; 1981. Dimensions 33.0x30.0 cm, book.
2. World Health Organization. International statistical classification of diseases and related health problems: ICD-10. 3, 3 ,. Geneva: World Health Organization; 2009.
3. Professional recommendations service / HAS. Proposals relating to individual screening in children aged 28 days to 6 years, intended for general practitioners, pediatricians, PMI physicians and school physicians. France: Haute Autorité de Santé, HAS; 2005 Sep p. 132.
4. Francoeur E, Ghosh S, Reynolds K, Robins R. An international journey in search of diagnostic clarity: early developmental impairment. J Dev Behav Pediatr JDBP. 2010 May; 31 (4): 338-40.
5. Shevell M, Ashwal S, Donley D, Flint J, Gingold M, Hirtz D, et al. CME Practice parameter: Evaluation of the child with global developmental delay. American Academy of Neurology. 2003 Feb 11; 60 (3): 367-80.
6. Lu C, Black MM, Richter LM. Risk of poor development in young children in low-income and middle-income countries: an estimation and analysis at the global, regional, and country level. Lancet Glob Health. 2016 Dec; 4 (12): e916-22.
7. Masaya AM, Cock PD, Natuhoyila AN, Ndosimao CN. Evaluation of the psychomotor development of the Congolese child by the Bayley scale for the development of children second edition. PAMJ - Clinical Medicine. 2020 Feb 18; 2 (56): 13.
8. Jimenez-Gomez A, Standridge S. A refined approach to evaluating global developmental delay for the international medical community. Pediatr Neurol. 2014;

9. Huffman SL, Schofield D. Consequences of malnutrition in early life and strategies to improve maternal and child diets through targeted fortified products. Matern Child Nutr. 2011 Sep 19; 7 (Suppl 3): 1-4.
10. Groce N, Challenger E, Berman-Bieler R, Farkas A, Yilmaz N, Schultink W, et al. Malnutrition and disability: unexplored opportunities for collaboration. Paediatr Int Child Health. 2014 Apr; 34 (4): 308-14.
11. van den Heuvel M, Voskuijl W, Chidzalo K, Kerac M, Reijneveld SA, Bandsma R, et al. Developmental and behavioral problems in children with severe acute malnutrition in Malawi: A cross – sectional study. J Glob Health [Internet]. [cited March 10, 2021]; 7 (2). Available at: https://www.ncbi.nlm.nih.gov/pmc/articles/PMC5735778/
12. Grantham-McGregor SM. Malnutrition, Mental Function, and Development. : 16.
13. Ruiz JDC, Quackenboss JJ, Tulve NS. Contributions of a Child's Built, Natural, and Social Environments to Their General Cognitive Ability: A Systematic Scoping Review. PLoS ONE [Internet]. 2016 Feb 3 [cited 2020 Dec 9]; 11 (2). Available at: https://www.ncbi.nlm.nih.gov/pmc/articles/PMC4739499/
14. Bornstein MH, Hendricks C. Screening for Developmental Disabilities in Developing Countries. Soc Sci Med 1982 [Internet]. 2013 Nov [cited 2020 Dec 9]; 97. Available at: https://www.ncbi.nlm.nih.gov/pmc/articles/PMC3638080/
15. Dasen PR, Super CM. The usefulness of a cross-cultural approach in studies of malnutrition and psychological development. In: Health and cross-cultural psychology: Toward applications. Thousand Oaks, CA, US: Sage Publications, Inc; 1988. p. 112-38. (Cross-cultural research and methodology series, Vol. 10).
16. Vygotsky L. Thought and language. Messidor / Social editions; 1985.
17. Van Rie A, Mupuala A, Dow A. Impact of the HIV / AIDS Epidemic on the Neurodevelopment of Preschool-Aged Children in Kinshasa, Democratic Republic of the Congo. Pediatrics. 2008 Jul; 122 (1): e123-8.
18. Bouville JF. The relational approach to child malnutrition in tropical settings. Sci Soc Health. 1996; 14 (1): 103-16.
19. Brown RE. Decreased brain weight in malnutrition and its implications. East Afr Med J. 1965 Nov; 42 (11): 584-95.
20. Barnet AB, Weiss IP, Sotillo MV, Ohlrich ES, Shkurovich M, Cravioto J. Abnormal auditory evoked potentials in early infancy malnutrition. Science. 1978 Aug 4; 201 (4354): 450-2.
21. AKIGUET-BAKONG S. Malnutrition and cognitive resources in infants aged 12 to 24 months. Sudlangues. 2008; (9): 63-84.
22. Liu J. Malnutrition at Age 3 Years and Externalizing Behavior Problems at Ages 8, 11, and 17 Years. Am J Psychiatry. 2004 Nov 1; 161 (11): 2005-13.
23. Tétinou F, Séraphin N, Nguefack F, Agbor A. Malnutrition in children with delayed psychomotor development. May 13, 2020;
24. Gorman KS. Malnutrition and cognitive development: evidence from experimental / quasi-experimental studies among the mild-to-moderately malnourished. J Nutr. 1995; 125 (8 Suppl): 2239S-2244S.
25. Freeman HE, Klein RE, Townsend JW, Lechtig A. Nutrition and cognitive development among rural Guatemalan children. Am J Public Health. 1980 Dec; 70 (12): 1277-85.

Annex 4: Acceptability study of a reduced dose of RUTF for the management of SAM children

1. Context and rationale

The present work is a sub-study nested within a clinical trial measuring the effectiveness of the reduced dose of RUTF in the management of SAM without medical complications. The study will take place in the Health Zones (ZS) of Nzaba and Bonzola in the province of Kasai Oriental in the DRC. This province experienced a great conflict between the militia of Kamwina Nsapu and the regular army of the DRC. The consequences of this conflict had very serious influences on the nutritional situation of children under 5 in the province. More than 30% and 33% of them suffer from malnutrition respectively in the ZS of Nzaba and Bonzola.

The treatment of SAM with RUTF has been implemented for over 15 years in different countries and settings with varying degrees of success. The advantages are clear in terms of decentralizing the treatment as close as possible to families since, for the least ill children, one medical and nutritional consultation per week is sufficient with the prescribed treatment to be taken at home. The cure rate often exceeds 75% for this type of outpatient care. However, few studies document the acceptability and uses of the nutritional product by families. Existing studies mainly focus on new formulations without dairy products, or with more essential amino acids, or a different essential fatty acid profile or even a variable zinc content.

The systematic review of the efficacy of SAM treatment with RUTF by Schoonees & al 2019 reports that RUTF remains effective in the management of SAM children compared to other dietary approaches (Schoonees & al, 2019).

A socio-anthropological study conducted in Burkina Faso at the start of the MANGO clinical trial indicated that RUTF is described as both medicine and food. This study also showed that RUTF is consumed by mothers of MAS children who believe that it will benefit the child through breastfeeding and the practices of sharing were essentially justified from a moral point of view (some parents or legal guardians cannot give RUTF to the SAM child while the other children watch without being able to have any) and cultural (it is common to share a new product with other members of the household). As for reducing the administered dose of RUTF, opinions were divided both at the level of beneficiaries and of health workers.

This study, which is carried out in a different context than that of Burkina Faso, aims to document the perceptions of families around the aspects of SAM treatment of one of the children of the family (benefits, constraints, side effects, sustainability), and practices linked

to the product (intra-family sharing of the ration, resale, mixing with other foods, daily distribution, under or over consumption over 7 calendar days, weekly balance or not, etc.), and in particular if the dose of RUTF during treatment in the context of the DRC.

2. Goals

2.1. Main objective

Evaluate the perceptions and practices of families, providers and influencers of children's health around the treatment of SAM without medical complications, and in particular with a reduced dose of RUTF, in order to judge the acceptability of a treatment SAM using a reduced dose of RUTF.

2.2. Specific objectives

- **Describe perceptions** families / care providers, influencers of children's health, around aspects of the treatment of SAM of one of the children in the family (benefits, constraints, side effects, durability, etc.), and in particular if the dose of 'RUTF during treatment.
- **Evaluate practices** (intra-family sharing of the ration and reasons, resale, mixing with other foods, daily distribution, under or over consumption over 7 calendar days, weekly remainder or not, etc.), and in particular if the dose of RUTF is reduced during processing.

3. Methodology

3.1. Participants and study area

The present study will take place in the Health Zones of Bonzola and Nzaba in the province of Kassai Oriental. The study will focus on the following populations:

- Parents or legal guardians of SAM children included in the study;
- Grandmother of children on RUTF
- Health care providers (health workers) involved in the management of SAM;
- Community relays involved in the care of SAM;
- Men and women in the general population
- Traditional healers and traditional healers
- Other resource persons could be identified during the study using the dynamic sampling approach.

3.2.Inclusion and exclusion criteria

- All designated targets present in the 14 health centers in the study area who have given their free and informed consent to participate in the study are eligible for the study.
- Targets outside the study area or targets in the study area who refused to participate will be excluded.

3.3. Sampling and sample size

Sampling will not aim for statistical representativeness but will be based on the representation of all key informants; the number of respondents and their choice then take into account the quality of the informant and the capacity of the singular situations analyzed to account for the phenomenon studied (Lefèbre P, 2002). Key informants will be chosen in each of the 2 health zones, either in health centers or in households and villages. The sample size per target is detailed in Table 1.

Table 1: Sample sizes according to tools and targets

**Collection technique**	**Collection tools**	**Targets**	**ZS from** Bonzola	**ZS from** Nzaba	**Total**
In-depth individual interview	Maintenance guide	Parent or legal guardians MAS children	3	3	6
		Grandmothers of MAS children	3	3	6
		Health workers	2	2	4
		Community relays	2	2	4
		Traditional healer	2	2	4
**Subtotal interview**					**24**
Direct observations	Observation Guide	Families of MAS children	2	2	4
**Subtotal direct observations**					**4**
Focus group	Focus group guide	Parents or legal guardians of MAS children	2	2	4
		General population woman	1	1	2
		Male of the general population	1	1	2
**Direct observation subtotal**					**8**

3.4. Data collection methods and techniques

3.4.1. In-depth interviews

Around 24 in-depth interviews will be carried out in the 2 health zones with the different targets in order to achieve saturation of information on the themes addressed. Interviews will be carried out using an individual interview guide. The interview will be audio recorded anddocument the following:

- Local names of the RUTF

- Perceptions of the product (access, effectiveness of RUTF on children's health, composition, taste, optimal use, perception of the quantity received, sufficient or not, etc.)
- Uses and consumption practices of RUTF (form of consumption, mixing with other foods, why of mixing, side effects, period of consumption, number of sachets consumed per day, number of sachets remaining per week)
- Sharing of RUTF with other children and members of the household (who else consumes: children, mother, grandmothers, fathers, why these shares: economic value of RUTF, social representation of RUTF (new product in the community); moral value (cannot give the product to one child while others watch and need etc).
- Level of acceptance of treatment with a reduced dose of RUTF (for or against a possible reduction in the dose, adherence to reduced RUTF-based treatment, implication of a reduction in RUTF in the community, etc.)

Parents or legal guardians of MAS children

Parents or legal guardians will be chosen in a representative (and not random) manner from among those present on site for the care of their SAM child. We will endeavor to choose a panel of diverse profiles (family tie = mother, grandmother, big sister, father, other family tie; age, profession, birth order of the child, cultural group). Parents or legal guardians of MAS children will be recruited from health centers.

Grandmothers of MAS children

Grandmothers of MAS children generally have a great influence on the care to be administered to children in the family space in the cultural context of Kasai. In this study, they are a source of key informants. Grandmothers of MAS children will be interviewed in homes other than those of parents or legal guardians if necessary

MAS Child Care Providers (Health Workers)

Health workers are health professionals involved in the care and monitoring of SAM children. They will be interviewed in health centers in the study area.

Most influential community representatives on child health

Children's health influencers will be identified during interviews with parents or legal guardians (dynamic sampling). This may involve community health workers, traditional

therapists, traditional midwives, community and / or religious leaders. They will be interviewed in health centers or in their homes.

3.4.2. Focus Group Discussions (FGD)

We will drive 8 FGDs with mothers and parents or legal guardians of MAS children, women from the general population, and men from the general population, providing information on the dominant trends and norms relating to our subject of study and will focus on the same themes as the interviews. The focus groups will be the subject of an audio recording followed by a transcription. Interviewers will use a focus-group interview guide + use of visual participatory tools.

3.4.3. Direct observations

At least 4 direct observations will be carried out within 4 families of SAM children included in the study, including 2 observations per health zone, lasting an "ordinary" family day during treatment (preferably , the first 2 days after obtaining the RUTF ration by the families). The observations will be recorded in a "logbook" according to the child's schedule with taking RUTF, preparing meals etc., and digitally on ODK / Kobo from the observations made.

3.4.4. Conduct of the investigation

Staff recruitment and training

The study will have to be carried out with few resources. As a result, it is the researchers who will conduct the planned interviews and focus groups after training, with the help of local translators and data entry officers, and the support of a French socio-anthropologist working with ACF.

Organization of the collection

The assessment of perceptions and practices will be carried out in two stages in order to document their evolution:

- On inclusion of children in the clinical trial,
- At the end of the study.

3.5.Data processing and analysis

The interviews carried out will be recorded on a dictaphone, translated into French, then transcribed in writing. A check of the transcription is made by the investigators themselves in order to ensure that the transcription is as faithful as possible to the comments made by the respondents. Data management will be done using Nvivo software according to the available license.The analysis will be based on a triangulation of the elements collected during the investigation. A textual analysis will be carried out according to the fields of the socio-anthropology of health.

3.6.Approval and ethical considerations

This study will respect the principles of general ethics in social sciences, and the principles in force in DR Congo. Shewill be part of the clinical trial which will be submitted to the opinion of the validation committee of the Ministry of Health and its partners, then to the National Ethics Committee.

To participate in this study, individuals must express additional consent to participate outside of the inclusion criteria of the overall study on the evaluation of the efficacy of the reduced dose of RUTF in the management of SAM. without medical complications. Thus the following ethical considerations will be respected:

Participant information: all participants will be informed about the object and objectives of the research. An information note will be prepared and read to participants. Those who wish can keep the information note

Free and informed consent: the interviews will be conducted only with those who have given their free and informed consent; consent will be oral.

Anonymity and confidentiality of data: To guarantee the anonymity of the participants, the interviews will be anonymous, the recordings agreed, translated, transcribed and numbered. No indication (name and first name, or location, etc.) allowing a participant to be identified will

appear in the search report. The data will be kept in a protected manner on the ACF cloud and ESP Kinshasa for the duration of the study until the actual final publications.

3.7.Expected results

- Perceptions around the treatment of SAM in DRC in these HZs, in particular among the 3 target groups, and in the event that the dose of RUTF is reduced;
- RUTF practices within families of SAM children and in communities including sharing, resale, mixing with other foods, durability of the prescribed dose until the next consultation, remaining RUTF per week, frequency of consumption per day, etc.

4. Bibliographical references

Ashworth A. 2006. Efficacy and effectiveness of community-based treatment of severe malnutrition. Food and Nutrition Bulletin 27: S24–48.

Briend A, Collins S. 2010. Therapeutic nutrition for children with severe acute malnutrition summary of African experience. Indian Pediatrics 47: 655–9.

Chaand, I., Horo, M., Nair, M. et al. Malnutrition in Chakradharpur, Jharkhand: an anthropological study of perceptions and care practices from India. BMC Nutr 5, 35 (2019)

Tadesse E, Berhane Y, Hjern A, Olsson P, Ekström EC. Perceptions of usage and unintended consequences of provision of ready-to-use therapeutic food for management of severe acute child malnutrition. A qualitative study in Southern Ethiopia. Health Policy Plan. 2015; 30 (10): 1334-1341.

Kajjura RB, Veldman FJ, Kassier SM. Maternal perceptions and barriers experienced during the management of moderately malnourished children in northern Uganda. Matern Child Nutr. 2020; 16 (4): e13022. Published 2020 Jul 6. doi: 10.1111 / mcn.13022

Kangas ST, Salpéteur C, Nikièma V, et al. Impact of reduced dose of ready-to-use therapeutic foods in children with uncomplicated severe acute malnutrition: A randomized non-inferiority trial in Burkina Faso. PLoS Med. 2019; 16 (8): e1002887. Published 2019 Aug 27.

UNICEF, Routine monitoring of the availability and use of ready-to-use therapeutic food (rutf) at the last mile (2018).

Lefèvre P, from Suremain CE. The contributions of socio-anthropology to public nutrition: why, how and under what conditions? Cah Détudes Rech Francoph. 2002; 12 (1): 77–85.

Yameogo Wme (2017). Final report. Study of perceptions and consumption of ready-to-use therapeutic foods (RUTF) in the health district of Fada N'Gourma in Burkina Faso

Information to be given before the start of the maintenance

Information

We are delighted that you were able to spare some of your time to participate in this discussion. We will first start by introducing ourselves. My name is ....

We are interested in your experience, ideas, comments, suggestions and recommendations on RUTF. This study will allow us to understand the representations and uses of ready-to-use therapeutic foods (RUTF). This will help us improve RUTF for the health benefit of malnourished children. All information will be kept confidential.

Annex 4.1. Interview Guide N ° 1 with parents or legal guardians and grandmothers of MAS children

Targets:

- Mothers of SAM children on RUTF
- Grandmothers of MAS children

Identification

- CSPS
- Town
- Dated
- Mother's age
- Mother's level of education
- Child's age
- RUTF consumed
- Duration of consumption:

I / Perception of malnutrition (manifestations, causes, consequence and treatment)

- What is malnutrition? How does it manifest itself?
- What do you think are the causes?
- How are children with malnutrition treated (traditional treatment)?
- Who will you see for your child if they are malnourished?
- What foods and drugs do you use to treat them?
- Do you think that we can cure malnutrition?
- What treatment have you found to be the most effective for your child's malnutrition?

II / Collection of RUTF

- What are the different types of RUTF that you know of? (Probe " RUTF 'red' = Plumpy'Nut and RUTF orange = Plumpy'Sup?)
- What is the difference between the two?
- What do you think of RUTF?
- What do RUTF contain?
- In which case / disease would you give RUTF to your child?
- Have you observed any positive effects of RUTF on malnourished children?
- Have you observed any negative effects of RUTF on the health of malnourished children?
- Do RUTF have positive effects sought / appreciated by other family members (especially adults)?
- Can RUTF be considered as normal foods / ingredients (reasons, desired effects)?
- What are the perceptions around RUTF ruptures?
- What about the amounts of RUTF given to children (insufficient, enough, too much)
- If we gave less, what would mothers think? (Efficiency)

III / Use of RUTF

- What RUTF is given to your child? (Probe RUTF 'red' (Plumpy'Nut) or ASPE orange (Plumpy'Sup)?
- How long has the child been consuming these foods?
- How easy is it to give RUTF to the child?
- At what time of day is the RUTF given; before or after other foods? Before or after breastfeeding?
- Where is the sachet stored during the day? Who could have access to it?
- Have you observed any difficulties giving RUTF to your child?
- What are the amounts (sachets) consumed by your child per day (Probe: recommended amount, less than recommended, more than amount)?
- Do you know how the number of RUTF sachets given to you was determined? (Why were you given this number of bags? Why has this number increased since the last time? Etc…)
- In how many days is the quantity received for a week consumed? Is it different during Ramadan? during the lean season? etc.
- Do you know the difference between (Plumpy'Nut) and ASPE orange (Plumpy'Sup)?
- If the child consumes less than expected, what use is made of the uneaten sachets or the remains of the opened sachet?
- Does it happen that RUTF sachets are shared with the siblings of the sick child or with other family members (reasons for sharing)
- In what form is RUTF given to the child? (Probe to mix in the porridge? In bread? Diluted in water)
- In what form does the child like RUTF (probe mix in porridge? In bread? Diluted in water)
- Does RUTF make the child thirsty? What drink does the child take with it?
- Does the child accept well to eat again the family dish after several weeks on the RUTF diet? or does it make difficulties?
- What are the strategies of mothers then so that the child eats the family dish?

IV / Child feeding under RUTF

- What other foods are given to sick children on RUTF?
- Have you observed any effects of these foods on the weight gain of the child? If yes, which ones ?
- In what situation do you give other foods to your child on RUTF?
- How effective are these foods compared to RUTF?
- For a child on RUTF, when are other foods introduced in his diet?
- Does the child latch on while being treated with RUTF? If so, before or after RUTF? how many times per day is the child breastfed while being treated with RUTF?
- Does breast milk affect the child's uptake of RUTF? If yes which ?
- Does RUTF (or treatment for malnutrition) affect breast milk intake? If yes which ?
- How do you feel about breast milk when the child is malnourished?
- How does breastfeeding work when the child is malnourished and has no RUTF?
- What positive effects have you observed of breastfeeding on the consumption of RUTF by the child?

What negative effects have you observed of breastfeeding on the consumption of RUTF by the child?

Perceptions of a reduction in the dose of RUTF

- Do you think SAM children are getting more RUTF than they need?
- What do you think of a possible reduction in the dose in the management of children?
- What impact can a reduction have on the community? adherence to treatment? Abandonment? Share ?

Annex 4.2. Interview guide N ° 2: Health workers

I / Identification

CSPS

Sex

Age :

Job :

II / Perception of malnutrition and recourse to healthcare

- What are the forms of malnutrition in the locality?
- What are the causes of malnutrition
- How do populations explain malnutrition?
- How do they traditionally treat malnutrition?
- When do mothers use the health center for malnutrition (State of the child)?

III / Use of RUTF

- What are the different types of RUTF available in your health center?
- Do you have any ruptures (when? Last rupture date when? And causes?)
- What is the process for prescribing child support (probing the child's first visit)
- What is the follow-up mechanism for the other visits (frequency, food delivered)
- What are the quantities of RUTF given to mothers?
- How do you make sure the child is consuming RUTF as prescribed?
- Do parents generally accept RUTF? (Why)
- What are the positive effects of RUTF on the health of malnourished children?
- What are the negative effects of RUTF on the health of malnourished children?
- What do you think about the amounts of RUTF given to children (insufficient, enough, too much)
- What do you think if we reduced the quantities given to children (would it be effective?)
- With the exception of children, who else consume RUTF in households (survey other sick or non-sick children?)

Do adults consume RUTF (for what effects: cite cases)

IV / Perceptions of a reduction in the dose of RUTF

- Do you think SAM children are getting more RUTF than they need?
- What do you think of a possible reduction in the dose in the management of children?
- What impact can a reduction have on the community? adherence to treatment? Abandonment? Share ?

Appendix 4.3: Interview guide N ° 3 for community relays

I / Identification

CSPS / village:

Profession:

Job :

Duration in the position:

II / Perception of malnutrition (manifestations, causes, consequence and treatment)

- What is malnutrition? How does it manifest itself?
- What do you think are the causes?
- How are children with malnutrition treated (traditional treatment)?
- Who will you see for your child if they are malnourished?
- What foods and drugs do you use to treat them?
- Do you think that we can cure malnutrition?
- What treatment have you found to be the most effective for malnutrition?

III / Collection of RUTF

- What do you think of RUTF? What do RUTF contain?
- What are the positive effects of RUTF on malnourished children
- What are the negative effects of RUTF on the health of malnourished children?
- Can RUTF be considered as food (reasons, desired effects)
- RUTF have effects in adults? (Which ?)

IV / Use of RUTF

- What are the quantities (sachets) consumed by the children per day (Probe: recommended quantity, less than recommended quantities, more than quantities)?
- What do you do when you find that the child is consuming less or more than the recommended amounts?
- If the child consumes less than expected, what use is made of the sachets not consumed?
- Who are the people who consume RUTF in households apart from sick children (Probe: other sick children in the household, other non-sick children)
- What do you think of this sharing practice?
- In your opinion, what are the reasons for this sharing (eco / social / cultural value?)
- In the community, are there adults who consume RUTF (Probe: Who in the household, desired effects)
- Are RUTF resold?

V / Child feeding under RUTF

- What other foods are given to sick children on RUTF?
- What are the effects of these foods on a child's weight gain?
- How effective are these foods compared to RUTF?
- For a child on RUTF, when are other foods introduced in his diet?
- What nutritional advice do you give to mothers of sick children under RUTF treatment?

VI / Perceptions of a reduction in the dose of RUTF

- Do you think SAM children are getting more RUTF than they need?
- What do you think of a possible reduction in the dose in the management of children?
- What impact can a reduction have on the community? adherence to treatment? Abandonment? Share ?

Appendix 4.4: Interview Guide N ° 4 for Traditional Healers / Traditional Healers

I / Identification

CSPS:

Town

Gender:

Age

II / Perception of malnutrition (manifestations, causes, consequence and treatment)

- What is malnutrition? (What is it called, how does it manifest?)
- What do you think are the causes?
- How are children with malnutrition treated (traditional treatment)?
- What traditional foods and medicines do you use to treat SAM children?
- What would you say to a mother who comes before you with a malnourished child seeking care? (Referral to a health center? Treated on site?)
- Do you have professional relations with the health center for malnourished children (orientation, follow-up, information exchange)

III / Food taboos

- In your village, what are the food prohibitions / taboos (animal meat, cereals, fruits, leaves, etc.) (name it / them and give the reasons)
- In your family, what are the food prohibitions / taboos (animal meats, cereals, fruits, leaves, etc.) (name them and give the reasons)
- What foods should you not eat in your community? (Name it / them and give the reasons)

IV Collection of RUTF

- What do you think of RUTF?
- How do you compare the effectiveness of RUTF compared to traditional products?

Are RUTF compatible with the treatments you give against malnutrition (how?)

Focus Group

Information to be given before the start of the discussion

Welcome to this discussion. We are delighted that you were able to spare some of your time to participate in this discussion. We will first start by introducing ourselves. My name is .... and I will moderate the discussion. In the team, we have .... who will take notes.

We are interested in your experience, ideas, comments, suggestions and recommendations on RUTF. This study will allow us to understand how to improve the representations and uses of ready-to-use therapeutic foods (RUTF). This will help us improve RUTF for the health benefit of malnourished children.

All information will be kept confidential.

Explain the rules of the discussion

This is a friendly discussion and therefore there is no right or wrong answer. We encourage you to relax and not hesitate to express and discuss your opinions. Participation is free and voluntary. We're going to provide the group with a series of open-ended questions or topics for discussion, and anyone who wants to can start responding. We will try to go around the group to hear a range of opinions from different people. As a reminder, you don't have to answer all of our questions, and you can skip questions. We would like to have only one person speaking at a time. When a person is speaking, there will be no interruption until that person has finished. The next person will then be invited to express their point of view. It does there will be no parallel discussions. Everyone can contribute to the discussion. You should feel free to agree or disagree, all in a cordial spirit. Please remember that what we are discussing here today is confidential. Do not discuss the private information disclosed in this group with others outside the group. Please silence your cell phones so as not to disrupt the discussion. We will spend about an hour and a half to two hours in our group discussion and you will be served refreshments at the end of the discussion. Please remember that what we are discussing here today is confidential. Do not discuss the private information disclosed in this group with others outside the group. Please silence your cell phones so as not to disrupt the discussion. We will spend about an hour and a half to two hours in our group discussion and you will be served refreshments at the end of the discussion. Please remember that what we are discussing here today is confidential. Do not discuss the private information disclosed in this group with others outside the group. Please silence your cell phones so as not to disrupt the discussion. We will

spend about an hour and a half to two hours in our group discussion and you will be served refreshments at the end of the discussion.

We have reviewed your consent forms, which describes the study in detail and gives us permission to speak to you. As a reminder, we will be using a digital recorder to record our conversation.

Do you have any questions before starting the discussion?

Moderator: switch on the digital recorder: "I am (NAME OF MODERATOR) and lead the group discussion (GROUP DISCUSSION IDENTIFICATION CODE) on [DATE] [START TIME] "

Annex 4.5. Focus Group Guide

Targets:

- Mothers of SAM children on RUTF
- Men of the general population
- Women in the general population

Identification

- Dated
- Num FG
- CSPS
- Group

I / Perception of malnutrition (manifestations, causes, consequence and treatment)

- What is malnutrition?
- How does it manifest itself?
- What do you think are the causes?
- How are children with malnutrition treated (traditional treatment)?
- Who will you see for your child if they are malnourished?
- What foods and drugs do you use to treat them?
- Do you think that we can cure malnutrition?
- What treatment have you found to be the most effective for your child's malnutrition?

II / Collection of RUTF

- What are the different types of RUTF that you know of? (Probe " RUTF 'red' = Plumpy'Nut and ASPE orange = Plumpy'Sup?
- What is the difference between the two?
- What do you think of RUTF? What are the different ways to get RUTF? If the RUTF market, what are the selling prices?
- What are the words of the mother towards her child / other children around RUTF?
- What do RUTF contain?
- In which case / disease would you give RUTF to your child?
- Have you observed any positive effects of RUTF on children (not malnourished)?
- Have you observed any negative effects of RUTF on children (not malnourished)?
- Have you observed any positive effects of RUTF on malnourished children?
- Have you observed any negative effects of RUTF on the health of malnourished children?
- Do RUTF have positive effects sought / appreciated by other family members (especially adults)?
- Can RUTF be considered as normal foods / ingredients (reasons, desired effects)?
- What are the perceptions around RUTF ruptures?
- What about the amounts of RUTF given to children (insufficient, enough, too much)
- If we gave less, what do mothers think? (efficiency)

III / Use of RUTF

- How easy is it to give RUTF to children?
- What time of day is RUTF given; before or after other foods? Before or after breastfeeding?
- Where is the sachet stored during the day? Who could have access to it?
- Have you observed any difficulties in administering RUTF to your children?
- What are the quantities (sachets) consumed by the children per day (Probe: recommended quantity, less than recommended quantities, more than quantities)?
- Do you know how the number of RUTF sachets given to you was determined? (Why were you given this number of bags? Why has this number increased since the last time? Etc…)
- In how many days is the quantity received for a week consumed? Is it different during Ramadan? during the lean season? etc.
- Do you know the difference between RUTF 'red' (Plumpy'Nut) and ASPE orange (Plumpy'Sup)?
- If the child consumes less than expected, what use is made of the uneaten sachets or the remains of the opened sachet?
- Does it happen that RUTF sachets are shared with the siblings of the sick child or with other family members (reasons for sharing)
- In what form does the child like RUTF (probe mix in porridge? In bread? Diluted in water)
- Does RUTF make the child thirsty? What drink does the child take with it?
- Does the child accept well to eat again the family dish after several weeks on the RUTF diet? or does it make difficulties? What are the strategies of mothers then so that the child eats the family dish?

IV / Child feeding under RUTF

- What other foods are given to sick children on RUTF?
- Have you observed any effects of these foods on the weight gain of the child? If yes, which ones ?
- In what situation do you give other foods to your child on RUTF?
- How effective are these foods compared to RUTF?
- For a child on RUTF, when are other foods introduced in his diet?
- Does the child latch on while being treated with RUTF? If so, before or after RUTF? how many times per day is the child breastfed while being treated with RUTF?
- Does breast milk affect the child's uptake of RUTF? If yes which ?
- Does RUTF (or treatment for malnutrition) affect breast milk intake? If yes which ?
- How do you feel about breast milk when the child is malnourished?
- How does breastfeeding work when the child is malnourished and has no RUTF?
- What positive effects have you observed of breastfeeding on the consumption of RUTF by the child?
- What negative effects have you observed of breastfeeding on the consumption of RUTF by the child?

Annex 4.6. Home observation grid

Indication: The investigator will stay a whole day in a household to note all the facts relating to the food consumption of children under RUTF.

The interviewer will be content to observe what is happening in the household with the help of the observation guide. It should remain neutral as much as possible without interfering in the actions and interactions observed. In case, for one reason or another, the investigator intervenes in the observed sequences, this should be clearly noted in the observation report.

Sequence description

Specify: Sequence observed

Place date ………………………………

Observed people

Child's age

Mother's age. …………………………………………. Mother's level of instruction

**RUTF consumption**	• Where are the RUTF kept (ask the mother at the end of the observation)?<br>• When are the RUTF given to the child? (Does the mother breastfeed the child first?)<br>• What are the quantities given at each moment and per day?<br>• How the child reacts (does he accept or not, asks for more?)<br>• How does the mother manage to get the child to take RUTF? (Is the child left alone to consume it?)<br>• Are there minimum hygiene rules followed before consuming RUTF? : hand washing, drinking water in a clean container / bowl consumed by the child while taking RUTF
**Consumption of other foods**	• What other foods are given to the child? (specify the form: solid, liquid, etc.)<br>• When are these foods given? (before / after RUTF?, at mealtimes? ...)<br>• Is it a food prepared for the child or taken from the family dish<br>• How much is given to the child (the unit will be that used by the mother: spoon, large or small ladle ...)<br>• How much of these products compared to RUTF (smaller, larger, or even<br>• Are these foods given at the same time as RUTF or at other times<br>• How children react to these foods (like or dislike)
**Sharing of RUTF in the household**	• Who else uses RUTF in the household?<br>• Does the mother drip when she gives the child (how much?)<br>• What use is made of the rest of the RUTF when the child does not consume the whole tablet?<br>• How other children react when the sick child is given RUTF (crowding around or indifference)<br>• How does the mother of the child react when the other children obviously want the sachets?

Annex 5: Economic evaluation

1. Context and rationale

The present work is a sub-study nested within a clinical trial measuring the effectiveness of the reduced dose of RUTF in the management of SAM without medical complications. The study will take place in the Health Zones (ZS) of Nzaba and Bonzola in the province of Kasai Oriental in the Democratic Republic of Congo (DRC). This province experienced a great conflict between the militia of Kamwina Nsapu and the regular army of the DRC. The consequences of this conflict had very serious influences on the nutritional situation of children under 5 in the province. More than 30% and 33% of them suffer from malnutrition respectively in the ZS of Nzaba and Bonzola.

A cost-effectiveness evaluation in the context of a reduction in the dose of RUTF in the management of SAM children showed that the reduced dosage of RUTF was economically beneficial for the treatment of malnutrition. : the cost of treating a child from admission to discharge in the reduced dosage arm of RUTF was $ 75 while the cost of treating a child with the standard dosage of RUTF was estimated at $ 91 for an average length of stay of 8 weeks on both sides (N'Diaye et al, 2021). However, an economic evaluation of the reduced dose has never been evaluated without additional staff to collect the data. A comparative study of reduced dose treatment compared to standard treatment in real conditions presents an opportunity to perform an economic evaluation in parallel. The hypothesis is that this could lead to a reduction in costs per child treated. This is the reason why this evaluation is proposed in the ZS of Nzaba and Bonzola in the province of Kasai Oriental in the DRC.

2. Goals

2.1.Main objective

To assess from a societal point of view, the economic cost of treating children with SAM without medical complications with a reduced dose of RUTF compared to treatment with a standard dose.

2.2.Specific objectives

The specific objectives of this study are:

- Estimate the costs from a societal point of view, including the costs borne not only by ACF and its partners but also by the beneficiaries of SAM treatment.
- Estimate the cost of SAM treatment for a child, with standard dose and with reduced dose
- Estimate the impact of a reduced dose on costs, taking into account its impact on efficacy results.

3. Methodology

3.1. Participants, interventions and outcomes

a. Study framework

The present work is a sub-study of a clinical trial measuring the effectiveness of the reduced dose of RUTF in the management of SAM without medical complications. The study will take place in the Health Zones (ZS) of Nzaba and Bonzola in the province of Kasai Oriental in the DRC.

b. Study population

It will be made up of children aged 6 to 59 months admitted to SAM treatment & included in the trial, ie 500 SAM children in each arm.

c. Type of economic evaluation

This is a parallel economic evaluation of the clinical trial, based on a static decision tree type model simulating the trajectory of all children with SAM included in the trial. SIf the non-inferiority hypothesis is verified in the DRC context, the type of analysis will be a Cost Minimization Analysis, otherwise it will be a Cost Effectiveness Analysis.

d. Societal Perspective

It includes all the costs borne by ACF and its partners, the health system and beneficiary households.

e. Efficacy data

The cure rate among all children enrolled in the study, and per arm, will be the main outcome of interest for the economic evaluation. We will explore the cure rate per arm at the end of the 12 weeks maximum treatment, and also we will be able to evaluate the cure rate including the children referred to UNTI who return to UNTA and positively complete their treatment.

f. Costs included

The costs included will relate to prospective costs that may modify the result, i.e. cost of the treated child including the costs of outpatient and inpatient treatment for children who will have complications:

- Direct medical costs (consultation costs for treatment, including drugs, exams, staff time, outreach activities, etc.)
- Direct non-medical costs (implementation time for staff, costs payable by beneficiaries),
- Indirect costs (loss of productivity of beneficiaries corresponding to the time spent receiving the consultation).

g. Excluded costs

Excluded costs (which do not change the result, for example the cost of the child treated):

- Research costs (researchers, team supervisors, research training)
- Costs of existing infrastructure (buildings, water, electricity, latrines, etc.)
- Support costs by ACF (ACF offices, ACF staff in DRC and Paris).

h. Time horizon of economic valuation

The cost study will only cover the period of enrollment of children in the study until the release of the last child in the cohort from treatment. This is currently estimated at a maximum of 3 months of treatment per child spread over several months, i.e. approximately 8 months from July 2021 to February 2022.

i. Expected results

a. Average cost per SAM child treated for a child on standard dose and on reduced dose
b. If a Cost Minimization Analysis then we will obtain a percentage of the total cost of treatment under reduced dose compared to that of children in the control group;
c. If a Cost Effectiveness Analysis then we will obtain a cost ratio per additional SAM child cured by the reduced dose approach

j. Sample size

The sample for this study will be made up of all the participants enrolled in the study at the end of the study.

3.2. Data collection and analysis

3.2.1. Data collection methods

a. Data collection technique

Data collection will consist of 2 modalities:

- Cost data :
  - Individual interviews with caregivers, caregivers, ACF staff, researchers

- Semi-structured group interviews with parents or legal guardians

- Study efficacy data :

this economic evaluation will use the efficacy study database, which will be made available within 6 months after obtaining the final efficacy results from the main study, i.e. after the final validation of these data by peers (after journal submission, actual review and re-submission of the revised article). The data will be anonymized by the principal investigator prior to data sharing.

b. Data collection plan

Staff recruitment and training

The collection of cost data will be delegated to existing ACF staff for the reduced dose acceptability sub-study, for which staff will be trained in individual and group interviews.

c. Organization of the collection

The collection of cost data will take place during the enrollment phase of the study, preferably for feasibility issues within health centers.

The collection of efficacy data will take place 1 year later or even later, when the data are validated by the principal investigator.

3.2.2. Data analysis

The estimated unit costs will be used as parameters in a decision tree model simulating the trajectory of all SAM children included in the clinical trial.

The model will follow each child in the cohort from registration to discharge by comparing the two groups. Each event modeled will be associated with its cost in the two treatment strategies in the DRC context.

The total costs of the two interventions compared will be calculated on the basis of the sum of each unit cost calculated for the nutritional follow-up consultation (weekly visits), adjusted to reflect the proportion of individuals who actually benefited from a follow-up visit in each arm of the clinical trial.

Cost data will be cleaned and analyzed using Microsoft Excel 2016 and STATA version 16 software and the decision analysis model will be developed with TreeAge software (TreeAge Pro 2017, Health Care Edition, Williamstown, MA).

The costs will be reported per consultation. The total cost of each arm will be presented globally, by type of cost (direct medical, non-medical, and indirect costs) and by major or

relevant expenditure item (RUTF, drugs, materials, consumables, human resources, communities).

No discount will be applied. Costs expressed in Congolese Francs before being converted into 2021 International Dollars using purchasing power parities.

Sensitivity analyzes will test the model and make it possible to evaluate the results obtained by the reduced dose in the best case and in a less favorable case.

3.2.3. Ethical aspects

The consent of the participants in the main study will include the mention of the economic evaluation.

This economic evaluation will be subject to the same ethical rules as the main study.

3.2.4. Dissemination of results

A publication will be submitted to a journal with a proofreading committee and in Open Access, ie without payment to be able to read it.

4. Bibliographical references

1. Kangas ST, Salpéteur C, Nikièma V, Talley L, Ritz C, Friis H, et al. Impact of reduced dose of ready-to-use therapeutic foods in children with uncomplicated severe acute malnutrition: A randomized non-inferiority trial in Burkina Faso. Persson LÅ, editor. PLOS Med. 2019 Aug 27; 16 (8): e1002887.
2. Schoonees A, Lombard MJ, Musekiwa A, Nel E, Volmink J. Ready-to-use therapeutic food (RUTF) for home-based nutritional rehabilitation of severe acute malnutrition in children from six months to five years of age. Cochrane Database of Systematic Reviews 2019, Issue 5. Art. No .: CD009000. DOI: 10.1002 / 14651858.CD009000.pub3.
3. N'Diaye, DS, Wassonguema, B, Nikièma, V, Kangas, ST, Salpéteur, C. Economic evaluation of a reduced dosage of ready-to-use therapeutic foods to treat uncomplicated severe acute malnourished children aged 6–59 months in Burkina Faso. Matern Child Nutr. 2021; 17: e13118.https://doi.org/10.1111/mcn.13118
4. Don Husereau, Michael Drummond & al. Consolidated Health Economic Evaluation Reporting Standards (CHEERS) —Explanation and Elaboration: A Report of the ISPOR Health Economic Evaluation Publication Guidelines Good Reporting Practices Task Force, Value in Health 16 (2013) 231–250.http://dx.doi.org/10.1016/j.jval.2013.02.002

Annex 6: Typology of Health Areas

Health Area	Geographic access	Living environment	PCIMA days	Population	Service use rate February 2021	Trained staff	Number of SAM children / month
**ZS Bonzola**							
Issue	Accessible	Urban	1 day (Thursday)	7 087	ND	6	
ND Grace	Accessible	Semi Urban	2 days (Monday and Friday)	24 160	ND	6	
Ksahala Bonzola	Accessible	Urban	Every day	15 372	33.7	4	
Tubondo 3	Accessible	Semi Urban	Every day	9 647	23.5	18	
Bimpe	Accessible	Urban	3 days (Tuesday, Thursday and Saturday)	16 879	ND	4	
Nyongolo	Accessible	Urban	ND	14 671	46.5	1	
Lubilanji	Accessible	Semi Urban	Every day	12,362	8.1	5	
Kasamayi	Accessible	Semi Urban	ND	18,279	29	4	
Ciaciacia	Accessible	Urban	1 day (Friday)	12 851	4.4	5	
Camp N'Sele	Accessible	Urban	6 days (Monday to Saturday)	10,419	2	4	
Cikisha	Accessible	Urban	3 days (Monday, Wednesday and Friday)	11 853	45.2	10	
Tubondo 2	Less accessible	Semi Urban	Every day	8,028	54.6	6	
The Grace of the Lord	Less accessible	Semi Urban	5 days	16,521	53.3	4	
Mudiba	Accessible	Urban	Every day	21,191	6.74	3	
Tubondo 1	Accessible	Semi Urban	ND	13,575	4.5	4	
**ZS Nzaba**							
Airport	Accessible	Urban	Every day	19 187	24	4	
Dinanga	Accessible	Semi Urban	5 Days (Monday to Friday)	18,412	2.4	5	
Of the market	Accessible	Semi-Urban	Every day	19 106	1	6	
Jerome	Accessible	Semi Urban	Every day	20,519	2.3	5	
Kidima Diba	Accessible	Semi Urban	All the day	17,984	1.1	6	
Lutulu	Accessible	Semi Urban	ND	21,779	28.9	7	
Luwaba	Less accessible	Semi Urban	1 day (Friday)	21,718	13	5	
Mayiba	Accessible	Semi Urban	Every day	19 525	17.3	4	
Mbikay	Less accessible	Urban	5 Days (Monday to Friday)	20,863	ND	6	
Mercy	Accessible	Urban	from monday to sunday	22 089	ND	7	
Mukankala	Accessible	Semi Urban	ND	20 814	19.5	7	
Mutombo katshi	Accessible	Semi Urban	Every day	19 973	81.1	5	
PMKO	Accessible	Semi Urban	Not integrated	8 089	29	4	
Tarmac I	Accessible	Semi Urban	5 Days (Monday to Friday)	20,914	8.1	8	
Tarmac II	Accessible	Urban	Every day	16,090	0.57	6	
Tatu muya	Accessible	Semi Urban	Every day	18 356	19.2	3	
Tudikolela	Accessible	Semi Urban	4 days (Monday to Thursday)	20,859	1.1	5	
Zimbabwe	Accessible	Semi Urban	6 days (Monday to Saturday)	21,241	50.5	9	

Annex 8: List of variables according to specific objectives

Specific objectives	Variables	Definition	Values	Ladder
**Primary objective**				
Evaluate, on an outpatient basis, without additional staff to collect data and in a food insecure situation, the effectiveness of a reduced dose of RUTF in the management of SAM with edema in children aged 6 to 59 months over the rate of weight gain (g / kg / d) of children aged 6 to 59 months from admission to discharge.	PB	Measurement of the Circumference of the arm halfway between the acromion between the olecranon in cm	Normal: ≥12.5cm<br>MAS: <11.5 cm<br>MAM: ≥11.5 and <12.5 cm	interval
	Cut	Child's length in cm	number	interval
	Weight	Body mass of the child in Kg obtained by weighing	number	interval
	Age	Number of months the child has lived since birth, reported by the caregiver or read on the growth card	number	interval
	PT index	Value obtained by comparing the weight of the child to the median weight of children of his height, obtained using a table	Normal: ≥-1 Z score<br>MAS: <-3 Z score<br>MAM: ≥-3 and <-2 Z score	Ordinal
	Presence of edema	Increased volumes of the lower limbs taking the bucket	Absent: normal volume<br>+ or ++	nominal
	Rate of weight gain in children	Ratio of the difference in weight between discharge and admission / initial weight / duration of treatment (g / kg / d)	number	interval
	SAM proportion without complications.	Ratio of all uncomplicated SAM children to the total of AD children received during recruitment	%	interval
	Sex	Child gender	Male<br>Feminine	nominal
	Level of education of the head of household	Level of study reached by the head of household	Any ; primary	ordinal
			Secondary; university	
	Head of household profession	Work performed by the head of household at the time of the study	Any ; Cultivator; Teacher ; civil servant; trader; taximan; other	nominal
	Household size	Number of people sleeping under the same roof sharing the same meal and who recognize the authority of the same chef	number	interval

Specific objectives	Variables	Definition	Values	Ladder
	Position in siblings	Position of the child among his biological siblings (at least one parent in common)	rank	ordinal
	Interbreeding interval	Number of months separating the child and his unborn child	number	interval
**Additional objectives**				
The duration of edema melting (in days) since admission.	Duration of onset of edema melting	Number of days between the start of treatment and the decrease in volume in the lower limbs	number	interval
	Duration of total melting of edemas	Number of days between the start of treatment and the complete disappearance of edema	number	interval
Evaluate the duration of treatment for each participant in the two groups	Duration of the treatment	Time between admission and discharge (PB≥12.5cm or PT ≥-3 Z score or edema melt)	number of days	interval
Assess dropout, cure, treatment failure and mortality rates	Dropout rate in each arm	Number of children who have abandoned treatment and who no longer come to the center even after 3 home visits / total of children followed in each arm	percentage	interval
	Recovery rate	Percentage of children having reached the discharge after 3 months of treatment	percentage	interval
	Proportion Transfer to hospital in each arm	Number of children being transferred to hospital during treatment (3 months) following a medical complication or if stagnation or loss of appetite / total children followed in each arm	percentage	interval
	Proportion of Deaths in each arm	Number of children who died during the stay at UNTA or during transfer to UNTI / total child followed in each arm	percentage	interval
Evaluate the cost and savings made with a reduced dose of RUTF compared to the standard dose	% savings in SAM treatment with reduced dose	Difference in cost between treatment with standard dose and treatment with reduced dose, expressed in%	percentage	interval
The growth velocity of anthropometric variables and indices	T / A index	Value obtained by comparing the height of the child to the median height of children of his age, obtained using a table	Normal: ≥-1 Z score<br>MAS: <-3 Z score<br>MAM: ≥-3 and <-2 Z score	Ordinal
**Secondary objectives**				
Evaluate the psychomotor development of before with	Motor skills	Abilities of the child to achieve voluntary or automatic movements of parts or the whole of his body		Interval

Specific objectives	Variables	Definition	Values	Ladder
MAS (both arms) after ttt in well-nourished children	Cognition	All the activities and internal processes inherent in the acquisition of knowledge, information, memory, thought, creativity, perception, as well as understanding and problem solving	Normal: > 70%<br>Delay: <70% in 2/4 areas	
	Language	Ability to express and perceive affective states, concepts, ideas by means of articulated signs or sounds		
	Sociability	Ability to interact with other members of the community (verbal or non-verbal: gaze, attitudes, gestures, etc.)		
Estimate acceptability of the reduced dose	Perception of dose reduction	Respondents' judgment regarding dose reduction	Nad<br>Less good<br>Good<br>Very good	ordinal
	Respondents' attitude	Respondents' favorable or unfavorable attitude towards reducing RUTF	No	ordinal
Assess relapse rates in each study group	Relapse rate at 4 months after discharge in each arm	Percentage of children with BP <12.5cm or PT <-3 Z score or reappearance of edema 4 months after discharge (healing) in each arm	Percentage	interval
Evaluate the concentration of micronutrients	Relapse rate at 4 months after discharge in each arm	Difference in amount of micronutrients (mg) in 100 ml of blood	Normal<br>Low (<normal amount)	interval
