# Supplementary material for: Effectiveness of a reduced dose of ready-to-use therapeutic food in community-based management of severe acute malnutrition: A non-inferiority randomized controlled trial in the Democratic Republic of Congo
Source: PLoS Med. 2025 May 16;22(5):e1004606. doi: 10.1371/journal.pmed.1004606 (PMC12084047; doi:10.1371/journal.pmed.1004606)
Supplement: S1 EfRAMAS statistical plan — (PDF) [file pmed.1004606.s002.pdf]

Statistical data analysis plan

Project: EfRAMAS

Effectivness of a reduced dose of Ready-to-use therapeutic food in uncomplicated severe acute malnutrition children compared with a standard dose

https://doi.org/10.1186/ISRCTN15258669

based on Protocol v7 20/12/2021

Version	Date	Updated on:	Clarifications
1	24/05/2022	First version	
2	11/06/2022	Tables and graphs	
3	22/09/2022	Main statistical analyses	
4	09/11/2022	Variables of interest	Variables to be considered for main outcom
5	02/12/2022	Itention to treat (ITT) and Per Protocol (PP)	Definition of criteria for ITT and PP
6	14/12/2022	Adjustments	
7	05/05/2023	Data management appendix	Protocol deviations / distribution error

Contributors

Names	Qualifacation	Contributions
Dr Julien Ntaongo Alendi	MD, Technical expert, Pronanut, PhD student	Design of the study protocol, questionnaires, information letter and consent application<br>Submission to the Ethics Committee<br>Declaration of the study on International Standard Randomised Controlled Trial Number (IRSCTN)<br>Contribution to the search for funding<br>Training of investigators and data collectors Sporadic supervision of data collection in the health center<br>Verification of data<br>Elaboration of the Statistical Analysis Plan Analysis of data<br>Interpretation of results<br>Writing as first author of the main scientific article<br>Submission of the article to a journal<br>Submission of the abstract to a conference and presentation
Dr Steve Botomba	MD, lecturer ESP	Design of the study protocol, questionnaires, information letter and request for consent<br>Contribution to submission to the<br>Ethics Committee Contribution to fundraising<br>Training of investigators and data entry personnel Sporadic supervision of data collection in the health center<br>Data verification<br>Contributions to the Statistical Analysis Plan<br>Contributions to data analysis<br>Interpretation of results<br>Contributions to writing the main scientific article
Prof Marie-Claire Muyer	MD, Head of Nutrition dept, ESP	Designing the study protocol, questionnaires, information letter and consent application<br>Contributing to submission to the Ethics Committee<br>Contributing to fundraising<br>Contributing to the Statistical<br>Analysis Plan<br>Contributing to data analysis Interpreting the results<br>Contributing to writing the main scientific article
JB Mayavanga	Head of MEAL unit, expert, Pronanut	Designing the study protocol, questionnaires, information letter and request for consent<br>Contributing to submission to the Ethics Committee<br>Contributing to fundraising<br>Training investigators and data entry staff<br>Sporadic supervision of data collection in the health center<br>Contributing to the Statistical Analysis Plan<br>Contributing to data analysis Interpreting the results<br>Contributing to writing the main scientific article
Victor Nikièma	PhD, Msc, AAH then IRC research coordinator	Design of the study protocol, questionnaires, information letter and consent form Submission to the Ethics Committee<br>Training of investigators and data entry officers Sporadic supervision of data collection in the health center<br>Data verification<br>Drafting of the Statistical Analysis Plan<br>Data analysis<br>Interpretation of results<br>Contributions to the drafting of the main scientific article
Cécile Salpéteur	Msc, Research project advisor in Nut & Health, AAH, France	Coordination of the study<br>Design of the study protocol, questionnaires, information letter and request for consent Submission to the Ethics Committee<br>Declaration of the study on IRSCTN<br>Contribution to the search for funding<br>Contributions to data verification

		Elaboration of the Statistical Analysis Plan<br>Interpretation of the results<br>Contributions to the writing of the main scientific article
Prof Patrick Kolsteren	PhD, MD, univ. Gent (Belgium)	Contributions to the Statistical Analysis Plan<br>Contributions to data analysis Interpretation of results<br>Contributions to writing the main scientific article
Dr Benjamin Guesdon	PhD, Research project advisor in Nut & Health, AAH, France	Contributions to the Statistical Analysis Plan<br>Contributions to data analysis<br>Interpretation of results<br>Contributions to writing the main scientific article

<u>Signatures</u>

SAP writer   Senior statistician   Clinical trial lead and co-lead

Julien Ntaongo   Prof Marie-Claire Muyer and Cécile Salpéteur

Contents

Liste des abréviations .... Erreur ! Signet non défini.
1. Introduction 5
2. Objective of the study 6
3. Study design 6
Intervention 7
Sample size 8
Selecting children 8
5. Non-inferiority hypothesis. 11
6. intention-to-treat versus per-protocol analysis 12
7. Flow of study participants; 13
8. Descriptive statisticss 13
9. General aspects of analysing the effects of the intervention 15
10. Weight gain velocity 15
11. Length of stay 16
12. Programmatic results 17
13. Événements indésirables graves et maladies 18
14. Relapse rate 20
15. Duration of edema melting 21
16. Références bibliographiques 22

Introduction

According to United Nations Children's Fund (UNICEF) estimates, around 10.4 million children suffered from acute malnutrition in 2021, particularly in the Democratic Republic of Congo (DRC) where over 30% of them live, i.e. around 3.3 million children (1,2). Paradoxically, it is in the most affected countries that access to severe acute malnutrition (SAM) treatment is most difficult (3). Treatment for children suffering from uncomplicated SAM on an outpatient basis consists of administering a broad-spectrum antibiotic and ready-to-use therapeutic food (RUTF), as well as other treatments if necessary, such as an antimalarial. This is integrated with inpatient care for children with complications, as well as community-based screening and follow-up (4-7).

Studies have shown that the dose of RUTF only allows a weight gain of between 1 and 5.5 g/kg/day in community care, in contrast to the theoretical expectation of 20 g/kg/day in hospital care (8). MAS children would therefore consume less RUTF than the dose distributed because of the sharing of RUTF (9,10). Based on adaptations to the protocol made by field workers (11), several scientific studies have tested the efficacy of a reduced dose of RUTF in the management of malnutrition (11-14). The reduced dose of RUTF in the management of acute malnutrition (moderate and severe) is as effective as the standard dose, even in a context of severe food insecurity (14). The reduced dose of RUTF is non-inferior to the standard dose in the management of acute malnutrition. In a so-called simplified approach (combined management of moderate acute malnutrition (MAM) and SAM with the same product), it is possible to manage a larger number of children, improve continuity of care between the treatment of SAM and that of MAM and thus prevent the deterioration of the condition of MAM children in SAM (12). The use of different recovery criteria between the two groups (intervention and control) in the cluster randomised trial in Sierra Leone (15) and the absence of a control group in the retrospective analysis of the community management of acute malnutrition (CMAM) programme in Myanmar (16) were major limitations in interpreting the results of these two studies. A randomised non-inferiority study conducted in Burkina-Faso (MANGO) showed that reducing the dose of RUTF after two weeks of treatment in children suffering from SAM without medical complications did not affect recovery or lengthen the duration of treatment, although a slight negative effect was observed on linear growth, especially in the youngest subjects (17). This MANGO study was carried out under optimal conditions with substantial additional resources and among a population with good food security, excluding children with nutritional edema, which requires verification that similar results can be obtained in a more

realistic setting before scaling up in other contexts. In the DRC, a number of approaches are being piloted, including one that involves treating all malnourished children with the same RUTF product, whether they are suffering from Moderate or Severe Acute Malnutrition. This would represent a three- to four-fold increase in the number of children to be treated by the healthcare system, which would require an increased logistics chain at national and regional level to bring the treatment as close as possible to these children. It is in this context that we are evaluating in the DRC the effectiveness of the reduced dosage of MANGO RUTF in a situation of severe food insecurity and under real conditions for children suffering from SAM, including those with nutritional edema. This strategy of reducing the dosage of RUTF to the most appropriate level for treating children suffering from SAM seems to us to be more promising and adapted to the context of the DRC.

This study was carried out in 14 health areas in the Bonzola and Nzaba health zones in Kasai Oriental.

This plan describes the planned statistical analyses of the EfRAMAS project data for the primary outcomes and some secondary outcomes.

The secondary outcomes - children's psychomotor development, degree of acceptance of a reduced dose of RUTF, savings in treatment costs - will each be the subject of a specific statistical analysis plan and are therefore not detailed in this document.

1. Objective of the study

The aim of this study was to evaluate the efficacy of a reduced dose of RUTF on the velocity of weight gain in medically uncomplicated SAM children aged 6-59 months compared with a standard dose.

2. Study design

The EfRAMAS project is an individually randomised, controlled, non-inferiority study comparing a group of SAM children receiving a reduced dose of RUTF, referred to as the "intervention group", with another group of SAM children receiving a standard dose of RUTF, referred to as the "control group".

Randomisation was carried out by a person independent of the study in Paris by block permutation using the online tool at www.randomisation.com. An individual randomisation list

was generated for each of the 14 health centers selected for the study in the Nzaba and Bonzola health zones in Kasaï Oriental. The allocation of children was 1:1 in each of the 2 groups.

Intervention

During the first 2 weeks, the 2 groups of children received the same quantity of RUTF and from the 3rd week of treatment, the intervention group's RUTF ration was reduced to 7 sachets per week for children weighing less than 7 kg and 14 sachets per week for those weighing more than 7 kg. The dose of RUTF for the control group remained the same, in accordance with the national protocol for the management of acute malnutrition. The treatment and monitoring of the children remained the same for the 2 groups of children throughout the duration of the treatment.

Table 1 : Dose of RUTF per group in number of sachets per week

Weight (kg)	Control group Standard dose		Intervention group Reduced dose			
	Admission-Discharge		Weeks 1-2		Week 3 - discharge	
	Sachets/week	Kcal/kg/d	Sachets/week	Kcal/kg/d	Sachets/week	Kcal/kg/d
3.0-3.4	9	168-190	9	168-190	7	147-167
3.5 - 4.9	11	183-204	11	183-204	7	102-143
5.0 - 6.9	14	155-214	14	155-214	7	72-100
7.0 - 9.9	21	144-204	21	144-204	14	101-143
10.0 - 14.9	28	144-214	28	144-214	14	67-100

The study was conducted in a double-blind fashion, and neither the participants nor the researchers knew their randomisation group. Only the head nurse delivering the intervention, the Action Again Hunger (AAH) field coordinator based in Mbuji Mayi and the study manager (lead for AAH) had the randomisation lists, as well as the AAH France data manager who created the lists for the study.

The acceptable weight gain was 5 g/kg/day for each participant, regardless of the group to which they belonged. The value of the acceptable weight gain was calculated from data for children treated for SAM in the same health zones before the start of this study. Non-inferiority was concluded if the mean weight gain was 4.5 g/kg/day in the intervention group. This mean weight gain was calculated on the basis of data from children treated for SAM within the care programme set up by AAH in the same health zones (Bonzola and Nzaba) in 2021.

Sample size

Assuming a power of 80% and a one-sided significance level α=0.05, a minimum of 335 children were required in each group to verify that the maximum real difference between the intervention groups was no greater than 0.5 g/kg/day. Taking into account a lost to follow-up rate of 20%, the total sample size was rounded up to 1,000 children. During the study in 2021, the sample size was increased by 117 children following the discovery of the use of the unisex Weight for Height table in health centres, which de facto leads to over-classification of MAM girls as MAS (18). Given that in the DRC, the unisex table for the weight-for-height index is used in real conditions, the main analyses will be performed on the full sample including patients identified as SAM because of this unisex table (18), then excluding them at the sensitivity analysis stage.

The randomisation lists were extended for each health centre to include this additional number of children.

Selecting children

Patients were recruited in the health centres, whether they came directly from the community, were screened by community health workers, were referred by the health centre or presented spontaneously for consultation. The community health workers were responsible for raising awareness at home in order to facilitate recruitment within the allotted time. They were paid $2 per child screened and admitted to the health centre.

If the child met the admission criteria, he or she was admitted to the study after the parents had signed a written consent form, informed by information given in the language of the family caregiver by the nurse in charge or his or her deputy (nurs). On admission, anthropometric measurements including weight, height, MUAC and nutritional edema were taken on all children.

Approximately 2 children were enrolled per day per health center, which took about 2 months to complete. Confidentiality was ensured for each individual who was selected for the study through pseudonymisation of questionnaires and data protection.

Inclusion criteria

To take part in this study, individuals had to meet the following criteria:

- Be aged between 6 and 59 months;
- Be SAM: PT<-3 Z-score and/or mid-upper arm circumference (MUAC) < 115mm and/or bilateral edema (+, ++);
- Have no medical complications;
- Have a good appetite test result;
- Live in the selected health zone;
- Consent to take part in the study (informed consent);
- Have no other sibling already admitted to the study. When there are several participants in the same sibling, only the first will be included in the EfRAMAS project. The others will be treated according to the national programme but will not be included in the study.

Non-inclusion criteria

- An inconclusive appetite test (negative test);
- Having another sibling (SAM) already enrolled in the study;
- Having a declared allergy to peanuts and/or milk;
- Having already received treatment for SAM in the last 6 months, including readmissions after dropping out of the programme, relapses and medical transfers;
- Malformation or disability or chronic pathologies that may affect food intake, such as cleft palate, cerebral palsy, trisomy 2, sickle cell anaemia, etc.;
- Having already received treatment for SAM in the last 6 months, including readmissions after dropping out of the programme, relapses and medical transfers.

Finally, the initial protocol did not include the safety analyses recommended for an efficacy trial. The authors wished to include these analyses after the fact, when data collection in the field was underway, but this was not possible due to the lack of an expert available quickly before the end of the children's treatment, and then due to the lack of time before the end of the treatment to make the data available to an internal expert at the very least (19).

The analyses for the secondary outcomes will consist of evaluating the effectiveness of the reduced dose in terms of :

- **Performance indicators:** compare the two groups in terms of cure rates, drop-out rates, mortality, non-responders and referrals to hospital,

- **Indicators** of the occurrence of serious adverse events or illnesses (bearing in mind that hospitalisation may have been refused by the family or the carer may have initiated a supervised feeding protocol rather than using referral), over the whole cohort. Serious adverse events include

1) Appearance of signs of serious medical complications (pneumonia, general dehydration, etc.),
2) Increase/development of nutritional edema,
3) Weight loss for 2 consecutive weighings,

4) Weight loss of more than 5% of body weight at any visit,
5) Stagnant weight for 3 consecutive weighings,
6) Failure of appetite test at any visit (7).

- **Rate of relapse as SAM or MAM** (according to initial definition) 3 months after cure, compared between the 2 groups.

Finally, exploratory analyses will be carried out for this outcome:

- **Duration of edema melt** (in days) since admission.

3. statistical principles applied to this study

Level of statistical significance

For this study, the level of significance was set at 0.05 (i.e. $p<0.05$).

Description and justification of the adjustment

For this study, we did not adjust for the multiplicity of analyses and the impact on type 1 error was not anticipated.

Confidence interval

The confidence interval was set at 95% for this study.

Adherence to the intervention –

According to the research protocol, to verify adherence to the intervention, the carer had to return the empty sachets of RUTF and the full but unconsumed sachets to health center in order to check that the child had indeed consumed the entire prescribed dose. The family carers were made aware of the fact that other members of the family should not take the RUTF instead of the child, even for reasons of breastfeeding or feeding the other children.

In the field, it was difficult for the family carers to return all the sachets of RUTF distributed the previous week. To check adherence to the treatment, the community health workers organised surprise visits to households to check that it was the MAS children who were actually consuming the RUTF.

Direct observations were carried out in the field as part of the acceptability study, conducted in parallel with the clinical trial, and interviews (individual and focus group) were conducted with various groups highlighting the prevailing food insecurity and widespread sharing of any available food, including RUTF.

The research teams (AAH, School of Public Health-Kinshasa and National Nutrition Program) trained the providers at the start of the study and provided supervision with more than 24 weeks spent in the field by one or other of the researchers in rotation.

Deviations from the protocol

Some family caregivers registered the same child in several health centers at the same time in order to have more RUTF. At the first visit, after the history-taking with the family carer, in order to check whether the child had already been registered elsewhere, the providers placed a few sachets of RUTF on the floor and the children who had already benefited from the SAM

treatment rushed to pick them up without the family carer ordering them to do so. If the nurs had any doubts, he would admit the child to the programme but not to the research. An AAH supervisor would come and take photos and dissuade the mothers from cheating.

Children who received the wrong dosage (randomisation error) at the start of or during treatment will be included in the intention-to-treat analyses (ITT - the maximum number of children enrolled in the study is kept, regardless of their discharge, for generally poorer results, but reflecting reality), but not in the per protocol analyses (PP - only children who are cured and who have received the planned treatment perfectly are kept, giving ideal results).

According to the research protocol, in order to be declared cured, participants had to meet the criteria on two successive weeks. In practice, some children were classified as cured after one week instead of two. For these children, they will be included in the ITT analysis and will then be the subject of a sensitivity analysis to determine whether or not they influence the final results. A protocol violation is defined as an incorrect randomisation at the outset or an error in the dose received more than 2 times during the course of treatment.

If 1 or 2 dose errors max are observed during the course of treatment and from the 4th week onwards, the child will be classified in 1 other category "distribution error". The child is then kept in the same group for analysis.

If 1 dose error occurs at any time before the 3rd visit, this is a breach of protocol. The child is then excluded from the PP analyses.

4. Non-inferiority hypothesis.

The null hypothesis (H0) states that the reduced dose is not non-inferior to the standard treatment in terms of velocity of weight gain. This hypothesis includes any result where the one- or two-tailed 95% CI is greater than the non-inferiority margin of 0.5 g/kg/d. Whereas the alternative hypothesis (H1) of non-inferiority is accepted if the difference in weight gain velocitys less than 0.5g/kg/d including the lower bound of the one-sided 95% confidence interval (20,21).

If the difference between the 2 groups is greater than 0.5 g/kg/d, including the two-sided 95% CI, the intervention can be considered inferior.

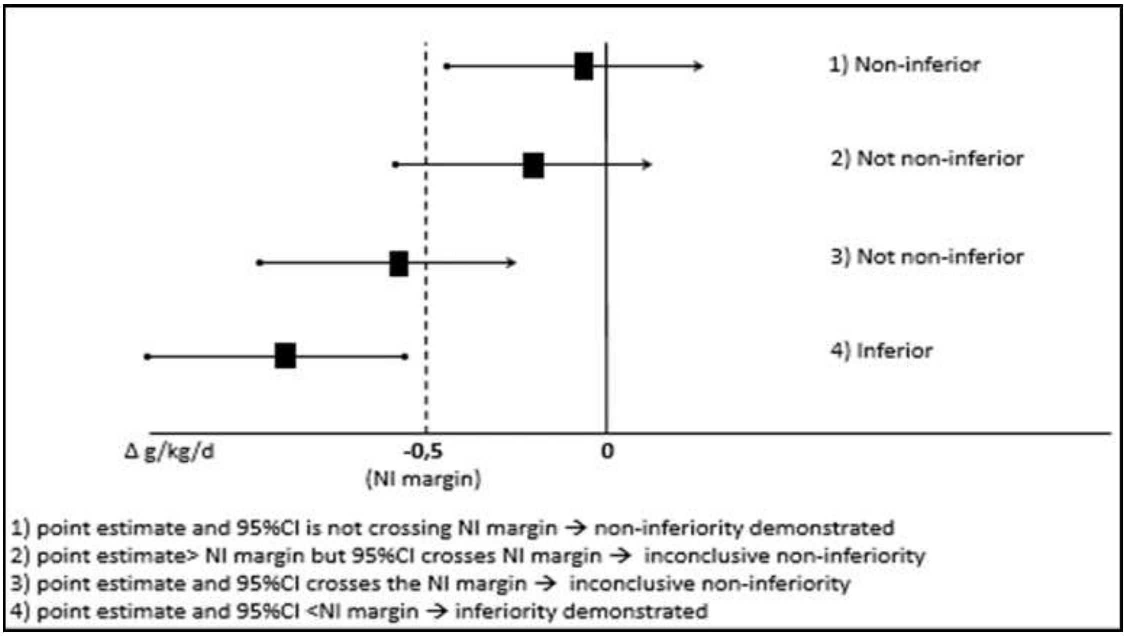

Figure 1: Possible results of the non-inferiority hypothesis

For the secondary outcomes, it will first be necessary to check whether the statistical power is sufficient before launching the analyses to test the hypothesis of non-inferiority.

5. intention-to-treat versus per-protocol analysis

In accordance with the recommendations for non-inferiority trials, two types of analysis will be performed for the primary endpoint of weight gain velocity (21-23):

- intention-to-treat (ITT) analysis including all randomised children (more realistic result)
- and per-protocol (PP) analysis considering only those who received the treatment.

The same will be done for length of stay and performance indicators (cure rate, drop-out rate, mortality rate, non-responders, referrals to inpatient in order to understand possible differences between the ITT and PP analyses.

The ITT analysis will include all subjects randomised to the study who have data on the variables of interest, regardless of any protocol violations, i.e. children: cured (even if only 1 visit), dropouts, deaths, referred to inpatient, non-responders (even if truly cured but not discharged on time, or stayed longer than 16 wk or other), consent withdrawals (all data up to actual withdrawal).

The PP analysis will include children who were correctly randomised and who completed the treatment, those declared cured, deceased, who received the treatment according to the research protocol and declared cured or non-responders (having exceeded 12 weeks of treatment without reaching the exit criteria) but will exclude confirmed dropouts, lost to follow-up, randomisation errors and withdrawals of consent (Figure 2).

According to the preliminary results of the qualitative survey carried out as part of this study, siblings frequently shared the RUTF, even though family carers were keen to comply with the nurses' instructions, because of the high level of food insecurity in the two health zones. We therefore postulate that MAS children consume around 50% of the quantity of RUTF distributed to them. This will be our starting hypothesis for the main analyses.

Sensitivity analyses will be carried out:

- On the main outcome to check whether or not a robust result is obtained, depending on whether the assumption of consumption of RUTF is low (25% of the prescribed dose is consumed) or high (75% of the prescribed dose is consumed);
- Definition of recovery: To see if anthropometric measurements improve if children are discharged after two consecutive weeks;
- Excluding girls MAM but who were classified as MAS due to the use of the unisex table;
- Excluding children from health center Dubai whose data quality is not good.

6. Flow of study participants;

On admission, for each participant, the inclusion criteria are checked: weight fo height Z-score (WHZ) < - 3 and/or MUAC < 115mm and/or bilateral edema (+, ++). The flow of these eligible children will be indicated according to the different phases: admission, management, follow-up and analysis. Children who do not meet the established criteria will be excluded from the final analyses. This includes those who have already been randomised but whose measurements, after verification, turn out to be erroneous. Even after discharge from treatment, children declared cured benefited from anthropometric and medical follow-up to assess the relapse rate for up to 3 months after discharge from treatment.

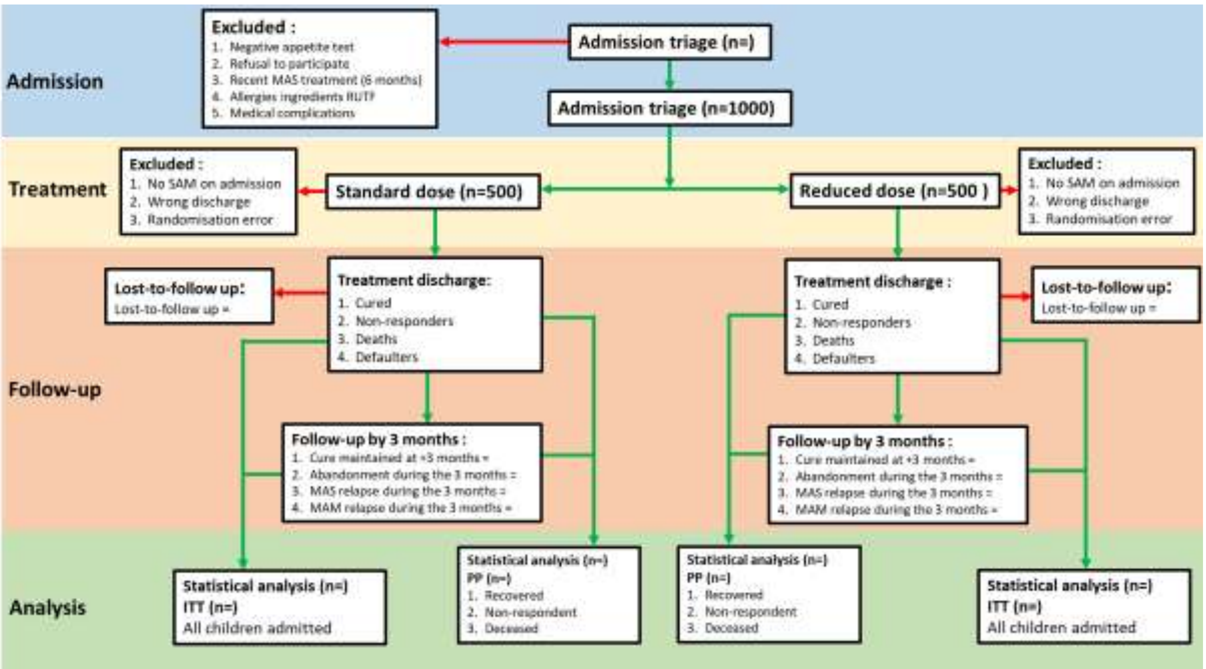

**Referred to inpatient = Treatment failure, these are children who have shown signs of danger.*

Figure 2: Patient flow in the EfRAMAS trial

7. Descriptive statisticss

Descriptive statistics will be used to describe the characteristics of participants in the two groups included in the intention-to-treat analysis.

For continuous variables, we will visually inspect the bell curves to assess whether they are normally distributed within the intervention groups, with a log transformation of variables with a log normal distribution. Population characteristics (Table 1) will be reported by intervention

group in the form of proportions, means (SD), geometric mean (geometric SD) and median (25th and 75th percentiles) as appropriate, with the corresponding group size.

Tableau 2 : Description of the basic characteristics of the children and their families in the 2 groups

Variable	Type	Units/categories
Age	continuous	Months
Sex	binary	% Male Female
Weight	continuous	Kg
Height	continuous	cm
MUAC	continuous	mm
Edema	binary	Yes, no
Admission criteria	ordinal	% WHZ<-3 et MUAC>= 115mm, % MUAC < 115 mm et WHZ >=-3, % WHZ<-3 et MUAC < 115 mm, % œdèmes, dont 3 sous catégories (% oedèmes et WHZ<-3, % oedèmes et MUAC<115 mm, % oedèmes et WHZ<-3 et MUAC<115 mm)
WHZ	continuous	Z-score
HAZ	continuous	Z-score
WAZ	continuous	Z-score
Diseases / follow-up acute respiratory infection according to family carers	ordinal	%fever, % acute respiratory infection, %malaria (fever), %diarrhoea*, %weight loss, %lack of appetite, %vomiting, %cough, %other (dermatological problems, other pathologies HIV, Tuberculosis, measles)
Medical treatment given by TSTs	ordinal	Amoxicillin 7d Y/N, albendazole Y/N, antimalarial Y/N or a score (to count who has had the 3 treatments, or 2 or 1)
Maternal age	continuous	years
Maternal education	binary	% no formal education
Cultural group	ordinal	Detail the main categories
Proportion of children still breastfed on admission	continuous	%
Profession of head of household	ordinal	Detail the main professions declared
Proportion of households without access to water in the previous month (Q83)	ordinal	Classify according to answers into 3 categories (<5d in the month ; between 5 and 15 days in the month more than 15 days in a month)
Household Food Insecurity (Access Scale) score	ordinal	% food security % mild food insecurity % moderate food insecurity % severe food insecurity
Urban or rural health centre	binary	Urban, rural

Age: between 6 and 59 months on admission
MUAC: less than 115mm if used as an admission criterion
WHZ, weight-for-height index
TAZ: height-for-age index
PAZ: weight-for-age index
ARF: Acute Respiratory Failure
Medical treatment: systematic medical treatment that all children admitted to the health center receive. It includes systematic deworming, vitamin A supplementation and vaccination where necessary.
Maternal education: level of education of the mother of a child admitted to health center
HFIAS (Household Food Insecurity Access Scale): Household Food Access Scale

8. General aspects of analysing the effects of the intervention

Where possible, effects will be evaluated. P-values and 95% confidence intervals, when reported, will be two-tailed unless otherwise stated. Outcome variables by intervention group will be described as proportions, means (standard deviation), geometric means (geometric standard deviation) and medians (25th and 75th percentiles) as appropriate, with corresponding group sizes. Intervention effects will be assessed as absolute differences (proportions and means) or relative differences in geometric means (variables with lognormal distributions) between groups. To facilitate interpretation, the Odds Ratios resulting from these analyses will be converted into differences in proportions.

To enable the results to be extrapolated to other contexts, we will also consider reporting relative effects for binary outcomes (i.e. proportion ratios). Results that are not normally distributed, even after log transformation, will be compared using non-parametric tests (for example, the Mann-Whitney U test).

9. Weight gain velocity

Weight measured at each weekly visit will be used to calculate weight gain velocity.

The difference in weight gain velocity between the two groups will be analysed both

- for the total duration of treatment (admission to discharge) and
- for the period between week 3 of treatment and discharge (eliminating the first 2 weeks of treatment which are identical between the 2 groups).

The following formulae will be used:

a. Total weight gain velocity :

$$\frac{\left(\frac{Weight\ at\ discharge - weight\ at\ admission\ (g)}{weight\ at\ admission\ (kg)}\right)}{Lenght\ of\ stay\ (days)}$$

b. Velocity of weight gain from the change in dosage, either from the 2nd visit or, if data is missing from the 2nd visit, from the 3rd visit :

$$\frac{\left(\frac{Weight\ at\ discharge - \ Weight\ at\ 3rd\ week\ of\ visit\ (g)}{weight\ at\ admission\ (kg)}\right)}{Lenght\ of\ stay\ - 21\ (days)}$$

Table 3 : Statistical analysis of weight gain velocity

Point final	Primary analysis	Additional analysis
**Total rate of weight gain from admission to discharge**	<u>**Non-inferiority :**</u><br>● Linear mixed-effects model of log*-transformed data (see below)<br>● One-tailed test (α=0.05)<br>● ITT and PP	**(See below)**
	<u>**The difference between control and intervention:**</u><br>● Linear mixed-effects model on log* transformed data<br>● Two-tailed test (α=0.05)<br>● ITT and PP<br>● Separately for cured children only.<br><u>*Fixed effects:*</u><br>- Intervention<br><u>*Random effects :*</u><br>- Health centre<br>- Research team<br>- Participant identifier<br><u>*Potential confounding factors:*</u><br>- Weight on admission,<br>- MUAC on admission,<br>- WHZ score on admission,<br>- Duration of treatment	<u>**Interaction test (ITT only)**</u> **:**<br>- T-test on sub-groups of children<br><u>*Potential effect modifiers*</u><br>- sex<br>- age category on admission (<12 months/≥12 months),<br>- admission criteria (WHZ and MUAC),<br>- Presence of bilateral edema<br>- Episode of morbidity during treatment<br>- Month of admissionSi interaction : analyse de sous-groupe par âge, sex, dose<br><u>**Unadjusted model**</u>
**Rate of weight gain after the first 2 or 3 weeks**	As above	As above

*Weight gain data should be skewed to the right

10.Length of stay

The length of stay will be calculated in days elapsed between admission to the nutrition programme and discharge for all children in ITT and PP. A separate analysis will be carried out for the other discharge categories: referral to inpatient, drop-out and lost to follow-up.

For example, the duration may be shorter for referrals and dropouts, with the result that the overall duration is reduced in the ITT analysis, giving a false positive result.

Table 4 : Suggested statistical analysis for length of stay

Point final	Primary analysis	Additional analysis
**Length of stay**	<u>**The difference between control and intervention :**</u><br>● Linear mixed effects model on log* transformed data<br>● Two-tailed trial<br>● Both ITT and PP (as will inform possible difference in WGV)<br>● Separately for recover, referred and defaulted.<br><u>*Fixed effects:*</u><br>- Intervention<br><u>*Random effects :*</u><br>- Health centre<br>- Research team<br>- Participant identifier<br><u>*Potential confounding factors:*</u><br>- Weight on admission,<br>- MUAC on admission,<br>- WHZ score on admission,<br>- Duration of treatment	<u>**Interaction test (ITT only) :**</u><br>● T-test (if normally distributed, otherwise Man Whitney?)<br><u>***Potential effect modifiers***</u><br>- Sex<br>- Age category on admission (<12 months/≥12 months),<br>- Admission criteria (WHZ and MUAC),<br>- Growth retardation on admission<br>- Distance to CoS (<30min/≥30min),<br>- Presence of bilateral edema<br>- Episode of morbidity during treatment<br>- Visits missed during treatment<br><br>If interaction: sub-group analysis<br><br><u>**Unadjusted model**</u>

*Les données devraient être faussées vers la droite

11. Programmatic results

The performance of the treatment of undernutrition will be calculated as follows:

The denominator will be the total number of children admitted, i.e. all the children enrolled in the study, whatever their mode of discharge - i.e. cured, abandoned, dead, non-responders, referred to inpatient, lost to follow-up. Of course, the total number of children enrolled and the total number discharged must be equal. Taux de Guérison selon les enfants enrôlés dans l'étude

1) Recovery rate = number cured/total number of children admitted

$$\text{Recovery rate} = \frac{number\ cured}{total\ number\ of\ children\ discharged} = \frac{number\ cured}{total\ number\ of\ children\ admitted}$$

   a. Healing according to WHO
   b. Healing according to SPHERE
   c. According to 1 single visit and not 2 consecutive visits
   d. Healing according to MUAC alone
   e. Healing maintained at +3m
   f. Healing in relation to the different exit categories

2) $$\text{Death rate} = \frac{number\ of\ deaths}{total\ number\ of\ children\ discharged} = \frac{number\ of\ deaths}{total\ number\ of\ children\ admitted}$$

   a. Deaths by exit category

3) $$\text{Defaulters} = \frac{defaulters\ number}{total\ number\ of\ children\ discharged}$$

   a. Absent for 2 consecutive visits and not confirmed by a home visit,
   b. Absent for 2 consecutive visits and confirmed by a home visit
   c. Withdrawal from the various discharge categories

4) $$\text{Non-response rate} = \frac{Number\ of\ non-responders}{total\ number\ of\ children\ discharged}$$

   a. Non-respondent in relation to the various exit categories

5) $$\text{Inpatient reference rate} = \frac{number\ of\ referrals}{total\ number\ of\ children\ discharged}$$

   a. For medical reasons
   b. For stagnant weight or weight loss

6) $$\text{Lost-to-follow up rate} = \frac{number\ lost\ to\ follow-up}{total\ number\ of\ children\ admitted} = \frac{number\ lost\ to\ follow-up}{total\ number\ of\ children\ discharged}$$

   a. Lost sight of the different exit categories

To be declared cured, each participant must meet the cure criteria according to their admission category: WHZ index ≥ -1.5 z-score or MUAC ≥125 mm, or both, and absence of edema in all cases, either at two consecutive visits or at a single consecutive visit.

Z score categories at discharge will be calculated on the basis of admission and discharge data with WHO anthro. It will thus be possible to obtain the percentage of "strict recovery" (indices recalculated a posteriori) and the percentage of "recovery in routine practice" (declared as such by the nurses when they were not). Children who do not recover after 12 weeks of treatment are declared non-responders to the treatment. These include children who only met the recovery criteria for the 1st time at the 12th visit. Failures include confirmed dropouts, i.e. children who missed 2 consecutive visits, were found alive by the community health workers and confirmed that they had dropped out of treatment.

At the weekly medical visit, the reason for the absence was asked and the answers classified as:

1) Reasons related to treatment (Refusal to continue treatment, relapse, referral, staff strike, holiday, other)
2) Reasons related to ability to come to visits (Companion travelled, Companion ill, health center inaccessible,).

Lost to follow-up are children who have missed 2 consecutive visits and for whom no contact has been able to confirm the child if alive. In a sensitivity analysis on the programmatic results, the lost to follow-up will be placed first with the dropouts, then with the sick to see if this influences the potential differences between the 2 groups.

Table 5 : Suggested statistical analysis of programmatic results

**Point final**	**Primary analysis**	**Additional analysis**
**Programmatic results:**<br>- **cure,**<br>- **referral,**<br>- **abandonment,**<br>- **lost to follow-up,**<br>- **non-response,**<br>- **death**	**Difference between control and intervention**<br>● Mixed-effects logistic regression model<br>● Two-way trial<br>● ITT<br>● Separately for each outcome<br>*Fixed effects:*<br>- Intervention<br>*Random effects :*<br>- Health centre<br>- Research team<br>- Participant identifier<br>*Potential confounding factors:*<br>- Weight on admission,<br>- MUAC on admission,<br>- WHZ score on admission,<br>- Duration of treatment	**Interaction test (recovery only):**<br>- Chi-square<br>***Potential effect modifiers***<br>- Sex<br>- Age category on admission (<12 months/≥12 months),<br>- Admission criteria (MUAC, WHZ),<br>- Growth retardation on admission<br>- Distance to CoS (<30min/≥30min),<br>- Presence of bilateral edema<br>- Episode of morbidity during treatment<br>- Visits missed during treatment<br>If interaction: sub-group analysis<br>**Unadjusted model**
**Reason for fault**<br>- **treatment-related**<br>- **capacity-related**<br>- **study-related**	**Difference between control and intervention**<br>● Chi-square	

12. Événements indésirables graves et maladies

Serious adverse effects (SAEs) are defined as medical complications requiring patients to be hospitalised. In practice, they do not always lead to patients being transferred to the inpatient.

As SAEs do not always lead to referral to the inpatient, and are not systematically recorded as such, we need to identify "a posteriori" whether they occurred, by analysing the data, even when the children were not referred to the inpatient. We will identify SAEs at each weekly visit as follows:

- For the appearance of at least one serious sign of medical complications (fever, diarrhoea, coughing and vomiting): an SAE variable per weekly visit was created and recoded for each serious sign as SAE_s1, SAE_s2, SAE_s3, etc. in order to assess how the evocative clinical signs had evolved compared with those of the previous visit.
- For the increase/development of nutritional edema: an edema variable has been created and recoded as + (slight), ++ (moderate) or +++ (generalised) depending on the appearance of edema or an increase in its intensity compared with the previous visit
- For weight loss during 2 consecutive weighings: at each visit, we will check for negative weight gain between t-1 and t, and between t-2 and t-1, and so on for up to 12 complete visits;
- For weight loss of more than 5% of body weight : at each visit we will check for the presence of a drop in weight gain of more than 5% between t-1 and t, then between t-2 and t-1, and so on up to 12 complete visits;
- For weight stagnating for 3 consecutive weighings: at each visit we will check if (weight at t - weight at t-1) + (weight at t-1 - weight at t-2) <100 grams and no illness.

Serious adverse effects will also be classified as :

1) **Medical complications:** these include diarrhoea, vomiting, cough, fever and the appearance or worsening of edema. For each SAE, the frequency was specified;
2) **Weight loss** defined as weight loss ≥ 5% over 2 weeks with no apparent illness explaining this (according to the initial reference category 6)
3) **Stagnant weight** defined as no more than 100 g weight gain over 2 weeks with no apparent illness explaining this.

The type of symptoms and the estimated number of days the child was symptomatic are recorded as reported by the carer at each weekly visit. In this way, the frequency (how many visits an illness was reported at during the past week) and the total number of days of illness

during treatment can be estimated and compared. Only illnesses reported after admission from the first treatment visit will be taken into account.

Table 6 : Suggested statistical analysis for serious adverse events and diseases

Point final	Primary analysis	Additional analysis
**Serious adverse events (whether or not involving referral to hospital following detection of medical complications)**	**<u>Différence entre contrôle et intervention</u>**<br>● Chi-squared test<br>● Two-tailed trial<br>● ITT<br>● Separately for each main referral category (medical complication, weight loss and stagnant weight)<br>● If difference in total number → separately for each category.	**<u>Interaction test (recovery only):</u>**<br>- Chi-square<br>*<u>Potential effect modifiers</u>*<br>- Sex<br>- Age category on admission (<12 months/≥12 months),<br>- Admission criteria (MUAC & WHZ),<br>- Growth retardation on admission<br>- Presence of bilateral edema<br>- Episode of morbidity during treatment<br>- Month of admission<br>- Visits missed during treatment<br>- HFIAS score low or >= acceptable (2 categories)
**Disease frequency** (number of visits in which a disease is reported in the past week)	**<u>Différence entre contrôle et intervention</u>**<br>● Test U de Man Whitney (car les données devraient être non normales, mais peut-être log-normales → test t ?)<br>● essai bilatéral<br>● ITT	Si différence constatée : analyse par maladie
**Nombre de jours de maladie au cours du Treatment** (*total number)	**<u>The difference between control and intervention</u>**<br>● Man Whitney U test (as data should be non-normal, but possibly log-normal → t-test?)<br>● two-tailed trial<br>● ITT	If a difference is observed: analysis by disease The aim will be to define the weight of each disease (in no. of days/out of total no. of days for all diseases) and to identify the most "burdensome" diseases on the MAS treatment compared in the two bars of the study.

**Total number: this is the sum of the number of days during which the child had diarrhoea, fever, cough and vomiting.*

13.<u>Relapse rate</u>

The relapse rate will be calculated among children who have been cured of the initial MAS treatment after a 3-month follow-up. Four possible end points are defined for these children:

1) No relapse : Cure maintained at +3 months
2) Discontinued during 3 months follow-up
3) Relapsed as DSS at any time during the 3 month follow-up period
4) Relapsed as MAM at any time during the 3 month period DSS relapse is defined as a child presenting with WHZ<-3, and/or MUAC<115mm or any degree of bilateral edema within 3 months of being treated for DSS and declared cured.

MAM relapse is defined as having a -3≤WHZ<-2, and/or a 115 ≤MUAC <125mm and without DSS criteria (WHZ<-3 and/or MUAC<115mm and/or bilateral edema) within three months of being treated for malnutrition and declared cured.

Table 7 : Suggested statistical analysis for relapse rate

Point final	Analyse primaire	Analyse supplémentaire
**Relapse as MAS**	**<u>The difference between control and intervention :</u>**<br>● **Survival analyses**<br>● Two-way trial<br>● ITT	**<u>Testing interaction :</u>**<br>● Chi-square test (or perhaps Fisher's exact test as there are very few observations...)<br>*<u>Potential effect modifiers:</u>*<br>- *Sex,*<br>- *Age category at admission (<12months/≥12months),*<br>- *Admission criteria (WHZ & MUAC),*<br>- *Presence of bilateral edema*<br>- *Episode of morbidity during treatment*<br>- *Visits missed during treatment*<br>- *HFIAS score low or ≥ acceptable (2 categories).*

14. Duration of edema melting

The duration of edema melting will be calculated in days elapsed between admission to the nutrition programme and the date of complete disappearance of edema of the lower limbs for children admitted with edema. The edema must begin to melt by the 14th day after the start of treatment and the edema must have completely disappeared by the 21st day, otherwise the treatment will have failed.

Only cases of bilateral edema detected on admission are included. Edema that develops during the course of treatment is considered a medical complication, and the corresponding patients are referred to the inpatient. The latter cases are not taken into account when calculating the duration of edema melting.

Table 8 : Suggested statistical analysis for the duration of edema melting

Point final	Analyse primaire	Analyse supplémentaire
**Length of stay**	**<u>The difference between control and intervention:</u>**<br>● Linear mixed effects model on log* transformed data<br>● Two-tailed trial<br>● Both ITT and PP (as will inform possible difference in WGV)<br>● Separately for recover, referred and defaulted.<br>*<u>Fixed effects:</u>*<br>- Intervention<br>*<u>Random effects :</u>*<br>- Health centre<br>- Research team<br>- Participant identifier<br>*<u>Potential confounding factors:</u>*<br>- Weight on admission,<br>- MUAC on admission,<br>- WHZ score on admission,<br>- Duration of treatment	**<u>Interaction test (ITT only):</u>**<br>● T-test (if normally distributed, otherwise Man Whitney ?)<br>*<u>Modificateurs d'effets potentiels</u>*<br>- Sex<br>- Age category at admission (<12 months/≥12 months),<br>- Admission criteria (WHZ & MUAC),<br>- HFIAS score low or ≥ acceptable (2 categories)<br>- Episode of morbidity during treatment<br>- Visits missed during treatment<br>- Month of admission<br>If interaction: sub-group analysis<br>**<u>Unadjusted model</u>**
*Data should be skewed to the right		

All statistical analyses will be carried out using Stata software version 17.0 (Stata Inc., USA).

15.Références bibliographiques

1. Unicef. Selon les estimations, 10,4 millions d'enfants en République démocratique du Congo, dans le nord-est du Nigéria, au Sahel central, au Soudan du Sud et au Yémen souffriront de malnutrition aiguë en 2021 [Internet]. [cité 21 févr 2021]. Disponible sur: https://www.unicef.org/drcongo/communiques-presse/enfants-malnutrition-aigue-2021
2. Headey D. Impacts of COVID-19 on childhood malnutrition and nutrition-related mortality. The Lancet. 2020;396:3.
3. SOS Enfants. L'émaciation sévère : Une urgence passée sous silence qui ménace la survie des enfants [Internet]. Unicef; 2022 mai [cité 24 juin 2022] p. 20. Disponible sur: https://www.unicef.org/media/122336/file/UNICEF-CA-2022-Wasting-FR.pdf
4. Fore HH, Dongyu Q, Beasley DM, Ghebreyesus TA. Child malnutrition and COVID-19: the time to act is now. Lancet. 2020;396(10250):517-8.
5. Garza C, Onis M. Relationale for developing a new international growth référence. Food and Nutrition Bulletin. 2004;25(1).
6. WHO. WHO child growth standards and the identification of severe acute malnutrition in infants and children. A Joint Statement. Genève: WHO; 2009.
7. Ministère de la Santé / RDC. Protocole National Prise en charge de la Malnutrition Aigue. Kinshasa, République Démocratique du Congo: Ministère de la Santé / RDC; 2016 p. 227.
8. Schoonees A, Lombard MJ, Musekiwa A, Nel E, Volmink J. Ready-to-use therapeutic food (RUTF) for home-based nutritional rehabilitation of severe acute malnutrition in children from six months to five years of age. Cochrane Developmental, Psychosocial and Learning Problems Group, éditeur. Cochrane Database of Systematic Reviews [Internet]. 15 mai 2019 [cité 19 juin 2021]; Disponible sur: http://doi.wiley.com/10.1002/14651858.CD009000.pub3
9. Collins S, Sadler K. Outpatient care for severely malnourished children in emergency relief programmes: a retrospective cohort study. Lancet. 7 déc 2002;360(9348):1824-30.
10. Tadesse E, Berhane Y, Hjern A, Olsson P, Ekström EC. Perceptions of usage and unintended consequences of provision of ready-to-use therapeutic food for management of severe acute child malnutrition. A qualitative study in Southern Ethiopia. Health Policy Plan. déc 2015;30(10):1334-41.
11. James PT, Van den Briel N, Rozet A, Israël A, Fenn B, Navarro-Colorado C. Low-dose RUTF protocol and improved service delivery lead to good programme outcomes in the treatment of uncomplicated SAM : a programme report from Myanmar. Matern Child Nutr. oct 2015;11(4):859-69.
12. Bailey J, Opondo C, Lelijveld N, Marron B, Onyo P, Musyoki EN, et al. A simplified, combined protocol versus standard treatment for acute malnutrition in children 6–59 months (ComPAS trial): A cluster-randomized controlled non-inferiority trial in Kenya and South Sudan. Tumwine JK, éditeur. PLoS Med. 9 juill 2020;17(7):e1003192.
13. Kangas ST, Salpéteur C, Nikièma V, Talley L, Ritz C, Friis H, et al. Impact of reduced dose of ready-to-use therapeutic foods in children with uncomplicated severe acute malnutrition: A randomised non-inferiority trial in Burkina Faso. Persson LÅ, éditeur. PLoS Med. 27 août 2019;16(8):e1002887.
14. Cazes C, Phelan K, Hubert V, Boubacar H, Bozama LI, Sakubu GT, et al. Simplifying and optimising the management of uncomplicated acute malnutrition in children aged 6–59

months in the Democratic Republic of the Congo (OptiMA-DRC): a non-inferiority, randomised controlled trial. The Lancet Global Health. avr 2022;10(4):e510-20.

15. Maust A, Koroma AS, Abla C, Molokwu N, Ryan KN, Singh L, et al. Severe and Moderate Acute Malnutrition Can Be Successfully Managed with an Integrated Protocol in Sierra Leone. The Journal of Nutrition. 1 nov 2015;145(11):2604-9.
16. James PT, Van den Briel N, Rozet A, Israël A, Fenn B, Navarro-Colorado C. Low-dose RUTF protocol and improved service delivery lead to good programme outcomes in the treatment of uncomplicated SAM : a programme report from Myanmar. Matern Child Nutr. oct 2015;11(4):859-69.
17. Kangas ST, Kaestel P, Salpéteur C, Nikièma V, Talley L, Briend A, et al. Body composition during outpatient treatment of severe acute malnutrition: Results from a randomised trial testing different doses of ready-to-use therapeutic foods. Clinical Nutrition. nov 2020;39(11):3426-33.
18. en-net | Urgent: Weight for Height Z-scores: Unisex Z-score table used in West African countries vs. calculated Z score values [Internet]. [cité 7 nov 2022]. Disponible sur: https://www.en-net.org/question/1826.aspx
19. Harvard Catalyst. DATA AND SAFETY MONITORING GUIDANCE [Internet]. Harvard Clinical and Translational Science Center; 2020 [cité 18 avr 2023]. Disponible sur: https://catalyst.harvard.edu/wp-content/uploads/regulatory/DSMB-P_Guidance.pdf
20. Wiens BL. Choosing an equivalence limit for noninferiority or equivalence studies. Control Clin Trials. févr 2002;23(1):2-14.
21. E 10 Choice of Control Group in Clinical Trials. 2006;30.
22. Le Henanff A, Giraudeau B, Baron G, Ravaud P. Quality of Reporting of Noninferiority and Equivalence Randomized Trials. JAMA. 8 mars 2006;295(10):1147.
23. Christensen E. Methodology of superiority vs. equivalence trials and non-inferiority trials. Journal of Hepatology. 2007;8.
