# Supplementary material for: Effectiveness of a reduced dose of ready-to-use therapeutic food in community-based management of severe acute malnutrition: A non-inferiority randomized controlled trial in the Democratic Republic of Congo
Source: PLoS Med. 2025 May 16;22(5):e1004606. doi: 10.1371/journal.pmed.1004606 (PMC12084047; doi:10.1371/journal.pmed.1004606)
Supplement: S1 Text — (DOCX) [file pmed.1004606.s004.docx]

**PMEDICINE-D-24-02867R1**

**Effectiveness of a reduced dose of ready-to-use therapeutic food in community-based management of severe acute malnutrition: a randomized non-inferiority trial in the Democratic Republic of Congo**

## By Julien NTAONGO ALENDI and colleagues

**Supplementary material in**

Content

**[Table A. Programmatic outcomes* in the reduced and standard dose groups of children using PP analyses.](#_Toc196304606)** [2](#_Toc196304606)

[**Table B. Subgroup analysis of total weight gain velocity (g/kg/d)** 3](#_Toc196304607)

[**Table C. Subgroup analysis of weight gain velocity (g/kg/d) after two weeks of treatment** 4](#_Toc196304608)

[**Table D. Subgroup analysis of Length of stay (days)** 5](#_Toc196304609)

[**Table E. Subgroup analysis of recovery (percentage)** 6](#_Toc196304610)

[**Table F. Subgroup analysis of defaulted (percentage)** 7](#_Toc196304611)

[**Table G. Subgroup analysis of false discharge (percentage)** 8](#_Toc196304612)

[**Table H. Subgroup analysis of lost to follow up (percentage)** 9](#_Toc196304613)

[**Table I. Subgroup analysis of relapse (percentage)** 10](#_Toc196304614)

[**Table J. Subgroup analysis of serious adverse events and illnesses (percentage)** 11](#_Toc196304615)

[**Table K. Subgroup analysis of duration edema melting** 12](#_Toc196304616)

[**Table L. Subgroup analysis of MUAC gain velocity (mm/week)** 13](#_Toc196304617)

## **Table A. Programmatic outcomes* in the reduced and standard dose groups of children using PP analyses.**

| Outcome | Reduced RUTF | | Standard RUTF | | Unadjusted model |  | Adjusted model |  |
| --- | --- | --- | --- | --- | --- | --- | --- | --- |
|  | n |  | n |  | Difference (95% CI) | p-value | Difference (95% CI) | p-value |
| Recovery | 302 | 186 (61.59) | 329 | 217 (65.96) | -4.62 (-11.80; 2.56) | 0,21 | -1.53 (-7.64; 4.58) | 0,62 |
| False discharge | 302 | 97 (32.12) | 329 | 89 (27.05) | 5.27 (-1.47; 12.01) | 0,12 | 2.08 (-4.98; 9.15) | 0,56 |
| Non responders | 302 | 1 (0.33) | 329 | 1 (0.30) |  |  |  |  |
| Lost-to-follow up | 302 | 19 (6.29) | 329 | 21 (6.38) | -0.23 (-5.03; 4.57) | 0,93 | 0.81 (-4.16; 5.79) | 0,21 |
| Edema melting | 25 | 14 [0-14] | 22 | 7 [7-14] | 1.07 (-2.72; 4.87) | 0,58 | 1.14 (-2.77; 5.05) | 0,57 |
| Serious undesirable effects |  |  |  |  |  |  |  |  |
| Weight loss | 302 | 6 (1.99) | 329 | 1 (0.30) | 1.97 (-0.37; 4.31) | 0,07 | 1.15 (-0.32; 2.62) | 0,41 |
| Stagnant weight | 302 | 37 (12.25) | 329 | 42 (12.77) | 0.34 (-4.65; 5.34) | 0,89 | -0.44 (-5.69; 4.81) | 0,34 |
| Medical complication | 302 | 48 (15.89) | 329 | 48 (14.59) | 2.35 (-3.37; 8.07) | 0,42 | 1.56 (-4.48; 7.60) | 0,24 |
| LoS, days | 302 | 42 [35-49] | 329 | 42 [35-51] | -0.75 (-2.83; 1.33) | 0,48 | -0.48 (-2.49; 1.53) | 0,64 |
| Relapse | 142 | 4 (2.82) | 167 | 5 (2.99) | 0.46 (-2.36; 3.28) | 0,75 | -0.08 (-2.88; 2.72) | 0,24 |

** Values are presented as mean ± standard deviation, or n (%), or IQR [min, max].*

*RUTF=ready-to-use therapeutic food. CI = Confidence interval*

## **Table B. Subgroup analysis of total weight gain velocity (g/kg/d)**

| Variables | Reduced dose | | Standard dose | | Unadjusted difference (95% CI) | p | Adjusted difference (95% CI) | p |
| --- | --- | --- | --- | --- | --- | --- | --- | --- |
|  | n | Mean ± SD | n | Mean ± SD |  |  |  |  |
| Child age |  |  |  |  |  | <0.001 |  | 0.001 |
| - Age <12 mo | 49 | 6.53 ± 2.99 | 47 | 5.18±2.40 | 1.21 (0.26, 2.16) | 0.013 | 0.77 (-0.12, 1.65) | 0.090 |
| - Age ≥12 mo | 441 | 4.69 ± 2.21 | 431 | 5.08 ± 2.26 | - 0.37 (-0.63, -0.11) | 0.006 | -0.23 (-0.48, 0.02) | 0.071 |
| Child sex |  |  |  |  |  | 0.898 |  | 0.613 |
| - male | 268 | 4.93 ± 2.41 | 257 | 5.08 ± 2.18 |  |  |  |  |
| - female | 222 | 4.81 ± 2.31 | 221 | 5.11 ± 2.38 |  |  |  |  |
| Admission criteria |  |  |  |  |  | 0.686 |  | 0.761 |
| - MUAC only | 212 | 4.38 ± 2.11 | 217 | 4.51 ± 2.13 |  |  |  |  |
| - WHZ only | 121 | 5.47 ± 2.37 | 99 | 5.72 ± 2.34 |  |  |  |  |
| - Edema only | 36 | 3.08 ± 1.49 | 26 | 3.66 ± 2.13 |  |  |  |  |
| - MUAC and WHZ | 118 | 5.69 ± 2.52 | 135 | 5.85 ± 2.11 |  |  |  |  |
| - MUAC and Edema | 3 | 5.44 ± 2.19 | 1 | 6.05 ± 0.00 |  |  |  |  |
| Edema at any visit |  |  |  |  |  | 0.235 |  | 0.461 |
| - Absence of Edema | 451 | 5.02 ± 2.37 | 451 | 5.17 ± 2.26 |  |  |  |  |
| - Presence of Edema | 39 | 3.26 ± 1.64 | 27 | 3.75 ± 2.14 |  |  |  |  |
| Morbidity |  |  |  |  |  | 0.666 |  | 0.784 |
| - Yes | 400 | 5.11 ± 2.37 | 387 | 5.39 ± 2.28 |  |  |  |  |
| - No | 90 | 3.85 ± 2.04 | 91 | 3.81 ± 1.74 |  |  |  |  |
| Admission months |  |  |  |  |  | 0.172 |  | 0.267 |
| - Dry season | 219 | 4.58 ± 2.43 | 212 | 4.93 ± 2.26 |  |  |  |  |
| - Wet season | 271 | 5.11 ± 2.28 | 266 | 5.22 ± 2.29 |  |  |  |  |
| Missed visits |  |  |  |  |  | 0.546 |  | 0.079 |
| - Yes | 70 | 3.85 ± 1.75 | 71 | 4.16 ± 2.41 | - 0.40 (-1.00, 0.19) | 0.187 | -0.56 (-1.14, 0.01) | 0.876 |
| - No | 420 | 5.05 ± 2.41 | 407 | 5.26 ± 2.28 | - 0.19 (0.47, 0.09) | 0.184 | -0.02 (-0.28, 0.24) | 0.054 |

*MUAC=mid-upper-arm circumference. RUTF=ready-to-use therapeutic food. WHZ=weight for-height Z. SD = Standard deviation. CI = Confidence interval*

## **Table C. Subgroup analysis of weight gain velocity (g/kg/d) after two weeks of treatment**

| Variables | Reduced dose | | Standard dose | | Unadjusted difference (95% CI) | p | Adjusted difference (95% CI) | p |
| --- | --- | --- | --- | --- | --- | --- | --- | --- |
|  | n | Mean ± SD | n | Mean ± SD |  |  |  |  |
| Child age |  |  |  |  |  | 0.003 |  | 0.035 |
| - Age <12 mo | 46 | 5.82 ± 2.96 | 40 | 4.96 ± 2.79 | 0.93 (-0.19, 2.05) | 0.103 | 0.34 (- 0.66, 1.33) | 0.51 |
| - Age ≥12 mo | 409 | 4.08 ± 1.94 | 400 | 4.47 ± 2.15 | - - 0.37 (- 0.61, 0.13) | 0.002 | - 0.24 (- 0.47, - 0.00) | 0.048 |
| Child sex |  |  |  |  |  | 0.074 |  | 0.273 |
| - male | 247 | 4.34 ± 2.06 | 239 | 4.44 ± 2.13 | -0.03 (- 0.35, 0.30) | 0.866 |  |  |
| - female | 208 | 4.09 ± 2.20 | 201 | 4.61 ± 2.33 | -0.46 (-0.84, -0.09) | 0.015 |  |  |
| Admission criteria |  |  |  |  |  | 0.471 |  | 0.864 |
| - MUAC only | 198 | 3.67 ± 2.11 | 196 | 4.06 ± 2.39 |  |  |  |  |
| - WHZ only | 112 | 4.69 ± 188 | 92 | 4.83 ± 1.79 |  |  |  |  |
| - Edema only | 33 | 3.56 ± 1.71 | 26 | 3.60 ± 1.98 |  |  |  |  |
| - MUAC and WHZ | 110 | 5.03 ± 2.10 | 125 | 5.19 ± 2.06 |  |  |  |  |
| - MUAC and Edema | 2 | 7.74 ± 0 | 1 | 4.76 ± 0 |  |  |  |  |
| Edema at any visit |  |  |  |  |  | 0.779 |  | 0.736 |
| - Absence of Edema | 420 | 4.30 ± 2.13 | 413 | 4.58 ± 2.23 |  |  |  |  |
| - Presence of Edema | 35 | 3.80 ± 2.09 | 27 | 3.64 ± 1.96 |  |  |  |  |
| Morbidity |  |  |  |  |  | 0.436 |  | 0.754 |
| - Yes | 89 | 2.76 ± 2.05 | 87 | 2.74 ± 2.06 |  |  |  |  |
| - No | 366 | 4.62 ± 1.99 | 353 | 4.96 ± 2.04 |  |  |  |  |
| Admission months |  |  |  |  |  | 0.793 |  | 0.388 |
| - Dry season | 201 | 3.96 ± 2.20 | 190 | 4.15 ± 2.34 |  |  |  |  |
| - Wet season | 254 | 4.49 ± 2.05 | 250 | 4.80 ± 2.09 |  |  |  |  |
| Missed visits |  |  |  |  |  | 0.876 |  | 0.630 |
| - Yes | 58 | 3.48 ± 2.08 | 54 | 3.61 ± 2.41 |  |  |  |  |
| - No | 397 | 4.37 ± 2.12 | 386 | 4.65 ± 2.16 |  |  |  |  |

*MUAC=mid-upper-arm circumference. RUTF=ready-to-use therapeutic food. WHZ=weight for-height Z. SD = Standard deviation. CI = Confidence interval*

## **Table D. Subgroup analysis of Length of stay (days)**

| Variables | Reduced dose | | Standard dose | | Unadjusted difference (95% CI) | p | Adjusted difference (95% CI) | p |
| --- | --- | --- | --- | --- | --- | --- | --- | --- |
|  | n | Mean ± SD | n | Mean ± SD |  |  |  |  |
| Child sex |  |  |  |  |  | 0.016 |  | 0.050 |
| - male | 268 | 44.8 ± 15.1 | 257 | 43.5 ± 14.2 | 1.47 (-0.87, 3.81) | 0.217 | 1.48 (-0.77, 3.74) | 0.197 |
| - female | 222 | 43.9 ± 14.1 | 221 | 46.2 ± 14.3 | -2.47 (-5.02, 0.08) | 0.057 | - 1.72 (-4.15, 0.71) | 0.165 |
| Child age |  |  |  |  |  | 0.383 |  | 0.941 |
| - Age <12 mo | 49 | 49.5 ± 15.9 | 47 | 47.7 ± 14.9 |  |  |  |  |
| - Age ≥12 mo | 441 | 43.9 ± 14.4 | 431 | 44.5 ± 15.9 |  |  |  |  |
| Admission criteria |  |  |  |  |  | 0.265 |  | 0.165 |
| - MUAC only | 212 | 43.2 ± 13.2 | 217 | 44.5 ± 13.3 |  |  |  |  |
| - WHZ only | 121 | 42.6 ± 12.9 | 99 | 38.8 ± 12.1 |  |  |  |  |
| - Edema only | 36 | 32.8 ± 11.3 | 26 | 31.0 ± 8.41 |  |  |  |  |
| - MUAC and WHZ | 118 | 51.8 ± 16.3 | 135 | 52.3 ± 14.0 |  |  |  |  |
| - MUAC and Edema | 3 | 56.0 ± 12.1 | 1 | 49.0 |  |  |  |  |
| - Yes | 275 | 44.3 ± 14.3 | 276 | 44.3 ± 14.7 |  |  |  |  |
| - No | 203 | 45.4 ± 14.1 | 214 | 44.6 ± 14.6 |  |  |  |  |
| Distance to Health centre |  |  |  |  |  | 0.208 |  | 0.196 |
| - < 30 minutes | 293 | 43.7 ± 14.7 | 284 | 44.2 ± 14.8 |  |  |  |  |
| - > 30 minutes | 185 | 46.5 ± 13.4 | 206 | 44.9 ± 14.4 |  |  |  |  |
| Edema at any visit |  |  |  |  |  | 0.417 |  | 0.196 |
| - Absence of Edema | 451 | 45.6 ± 14.1 | 451 | 45.3 ± 14.5 |  |  |  |  |
| - Presence of Edema | 39 | 34.6 ± 12.8 | 27 | 31.6 ± 8.95 |  |  |  |  |
| Morbidity |  |  |  |  |  | 0.763 |  | 0.734 |
| - Yes | 91 | 52.1 ± 12.7 | 90 | 52.1 ± 14.9 |  |  |  |  |
| - No | 387 | 43.1 ± 14.1 | 400 | 42.7 ± 14.0 |  |  |  |  |
| Missed visits |  |  |  |  |  | 0.225 |  | 0.329 |
| - Yes | 71 | 48.7 ± 15.6 | 70 | 50.5 ± 17.4 |  |  |  |  |
| - No | 407 | 44.1 ± 13.9 | 420 | 43.4 ± 13.9 |  |  |  |  |
| Admission months |  |  |  |  |  | 0.036 |  | 0.081 |
| - Dry season | 219 | 45.4 ± 16.0 | 212 | 43.5 ± 14.2 | 1.80 (-0.92, 4.52) | 0.194 | 1.56 ( -1.20, 4.33) | 0.267 |
| - Wet season | 271 | 43.7 ± 14.2 | 266 | 45.8 ± 14.3 | -1.89 (- 4.12, 0.32) | 0.094 | - 1.20 (-3.21, 0.81) | 0.241 |

*MUAC=mid-upper-arm circumference. RUTF=ready-to-use therapeutic food. WHZ=weight for-height Z. SD = Standard deviation. CI = Confidence interval*

## **Table E. Subgroup analysis of recovery (percentage)**

| Variables | Reduced dose | | Standard dose | | Unadjusted difference (95% CI) | p | Adjusted difference (95% CI) | p |
| --- | --- | --- | --- | --- | --- | --- | --- | --- |
|  | n | Percentage | n | Percentage |  |  |  |  |
| Child sex |  |  |  |  |  | 0.226 |  | 0.531 |
| - male | 268 | 54.1 | 257 | 61.2 |  |  |  |  |
| - female | 222 | 77.6 | 221 | 73.3 |  |  |  |  |
| Child age |  |  |  |  |  | 0.948 |  | 0.368 |
| - Age <12 mo | 49 | 59.2 | 47 | 59.6 |  |  |  |  |
| - Age ≥12 mo | 441 | 64.9 | 431 | 67.8 |  |  |  |  |
| Admission criteria |  |  |  |  |  | 0.243 |  | 0.204 |
| - MUAC only | 212 | 70.8 | 217 | 69.6 |  |  |  |  |
| - WHZ only | 121 | 63.6 | 99 | 61.6 |  |  |  |  |
| - Edema only | 36 | 69.4 | 26 | 73.1 |  |  |  |  |
| - MUAC and WHZ | 118 | 51.7 | 135 | 65.2 |  |  |  |  |
| - MUAC and Edema | 1 | 100 | 3 | 66.7 |  |  |  |  |
| Stunting at admission |  |  |  |  |  | 0.505 |  | 0.497 |
| - Yes | 276 | 66.7 | 275 | 69.1 |  |  |  |  |
| - No | 214 | 61.2 | 203 | 63.0 |  |  |  |  |
| Health centre distance |  |  |  |  |  | 0.297 |  | 0.701 |
| - < 30 minutes | 284 | 61.6 | 293 | 67.9 |  |  |  |  |
| - ≥ 30 minutes | 206 | 67.9 | 185 | 65.4 |  |  |  |  |
| Edema at any visit |  |  |  |  |  | 0.690 |  | 0.417 |
| - Absence of Edema | 451 | 63.9 | 451 | 66.5 |  |  |  |  |
| - Presence of Edema | 39 | 69.2 | 27 | 74.1 |  |  |  |  |
| Morbidity |  |  |  |  |  | 0.071 |  | 0.237 |
| - Yes | 400 | 68.5 | 387 | 68.9 | -0.01 (- 0.33, 0.32) | 0.965 |  |  |
| - No | 90 | 45.6 | 91 | 58.2 | 0.51 (- 1.10, 0.77) | 0.089 |  |  |
| Missed visits |  |  |  |  |  | 0.116 |  | 0.138 |
| - Yes | 70 | 51.4 | 71 | 62.0 |  |  |  |  |
| - No | 420 | 66.4 | 407 | 67.8 |  |  |  |  |

*MUAC=mid-upper-arm circumference. RUTF=ready-to-use therapeutic food. WHZ=weight for-height Z. SD = Standard deviation. CI = Confidence interval*

## **Table F. Subgroup analysis of defaulted (percentage)**

| Variables | Reduced dose | | Standard dose | | Unadjusted difference (95% CI) | p | Adjusted difference (95% CI) | p |
| --- | --- | --- | --- | --- | --- | --- | --- | --- |
|  | n | Percentage | n | Percentage |  |  |  |  |
| Child sex |  |  |  |  |  | 0.058 |  | 0.028 |
| - male | 268 | 1.87 | 257 | 2.72 | -0.62 (-1.86, 0.60) | 0.318 | - 0.96 (-2.37, 0.45) | 0.183 |
| - female | 222 | 3.60 | 221 | 1.00 | - -1.58 (-0.24, 3.18) | 0.053 | 1.46 (-0.21, 3.14) | 0.086 |
| Child age |  |  |  |  |  | 0.870 |  | 0.832 |
| - Age <12 mo | 49 | 2.04 | 47 | 2.12 |  |  |  |  |
| - Age ≥12 mo | 441 | 2.72 | 431 | 1.85 |  |  |  |  |
| Admission criteria |  |  |  |  |  | 0.490 |  | 0.624 |
| - MUAC only | 212 | 4.72 | 217 | 2.30 |  |  |  |  |
| - WHZ only | 121 | 0.00 | 99 | 2.02 |  |  |  |  |
| - Edema only | 36 | 2.78 | 26 | 0.00 |  |  |  |  |
| - MUAC and WHZ | 118 | 1.69 | 135 | 1.48 |  |  |  |  |
| - MUAC and Edema | 3 | 0.00 | 1 | 0.00 |  |  |  |  |
| Stunting at admission |  |  |  |  |  | 0.898 |  | 0.745 |
| - Yes | 276 | 2.54 | 275 | 1.45 |  |  |  |  |
| - No | 214 | 2.80 | 203 | 2.46 |  |  |  |  |
| Health centre distance |  |  |  |  |  | 0.518 |  | 0.577 |
| - < 30 minutes | 284 | 3.87 | 293 | 2.39 |  |  |  |  |
| - ≥ 30 minutes | 206 | 0.97 | 185 | 1.08 |  |  |  |  |
| Edema at any visit |  |  |  |  |  |  |  |  |
| - Absence of Edema | 451 | 2.66 | 451 | 2.00 |  |  |  |  |
| - Presence of Edema | 39 | 2.56 | 27 | 0.00 |  |  |  |  |
| Morbidity |  |  |  |  |  | 0.561 |  | 0.900 |
| - Yes | 400 | 2.25 | 387 | 1.81 |  |  |  |  |
| - No | 90 | 4.44 | 91 | 2.20 |  |  |  |  |

*MUAC=mid-upper-arm circumference. RUTF=ready-to-use therapeutic food. WHZ=weight for-height Z. SD = Standard deviation. CI = Confidence interval*

## **Table G. Subgroup analysis of false discharge (percentage)**

| Variables | Reduced dose | | Standard dose | | Unadjusted difference (95% CI) | p | Adjusted difference (95% CI) | p |
| --- | --- | --- | --- | --- | --- | --- | --- | --- |
|  | n | Percentage | n | Percentage |  |  |  |  |
| Child sex |  |  |  |  |  | 0.114 |  | 0.251 |
| - male | 268 | 39.6 | 257 | 32.3 |  |  |  |  |
| - female | 222 | 14.4 | 221 | 17.6 |  |  |  |  |
| Child age |  |  |  |  |  | 0.946 |  | 0.742 |
| - Age <12 mo | 49 | 32.7 | 47 | 29.8 |  |  |  |  |
| - Age ≥12 mo | 441 | 27.7 | 431 | 25.1 |  |  |  |  |
| Admission criteria |  |  |  |  |  | 0.467 |  | 0.493 |
| - MUAC only | 212 | 19.3 | 217 | 20.7 |  |  |  |  |
| - WHZ only | 121 | 34.7 | 99 | 33.3 |  |  |  |  |
| - Edema only | 36 | 27.8 | 26 | 23.1 |  |  |  |  |
| - MUAC and WHZ | 118 | 38.1 | 135 | 28.2 |  |  |  |  |
| - MUAC and Edema | 3 | 0.00 | 1 | 0.00 |  |  |  |  |
| Stunting at admission |  |  |  |  |  | 0.620 |  | 0.485 |
| - Yes | 276 | 25.4 | 275 | 23.6 |  |  |  |  |
| - No | 214 | 31.8 | 203 | 28.1 |  |  |  |  |
| Health centre distance |  |  |  |  |  | 0.251 |  | 0.652 |
| - < 30 minutes | 284 | 29.9 | 293 | 23.5 |  |  |  |  |
| - ≥ 30 minutes | 206 | 25.7 | 185 | 28.6 |  |  |  |  |
| Edema at any visit |  |  |  |  |  | 0.805 |  | 0.493 |
| - Absence of Edema | 451 | 28.4 | 451 | 25.7 |  |  |  |  |
| - Presence of Edema | 39 | 25.6 | 27 | 22.2 |  |  |  |  |
| Morbidity |  |  |  |  |  | 0.331 |  | 0.409 |
| - Yes | 400 | 25.0 | 387 | 23.5 |  |  |  |  |
| - No | 90 | 42.2 | 91 | 34.1 |  |  |  |  |
| Admission months |  |  |  |  |  | 0.260 |  | 0.261 |
| - Dry season | 219 | 32.9 | 212 | 33.5 |  |  |  |  |
| - Wet season | 271 | 24.4 | 266 | 19.2 |  |  |  |  |
| Misseds visits |  |  |  |  |  | 0.253 |  | 0.288 |
| - Yes | 70 | 30.0 | 71 | 21.1 |  |  |  |  |
| - No | 420 | 27.9 | 407 | 26.3 |  |  |  |  |

*MUAC=mid-upper-arm circumference. RUTF=ready-to-use therapeutic food. WHZ=weight for-height Z. SD = Standard deviation. CI = Confidence interval*

## **Table H. Subgroup analysis of lost to follow up (percentage)**

| Variables | Reduced dose | | Standard dose | | Unadjusted difference (95% CI) | p | Adjusted difference (95% CI) | p |
| --- | --- | --- | --- | --- | --- | --- | --- | --- |
|  | n | Percentage | n | Percentage |  |  |  |  |
| Child sex |  |  |  |  |  | 0.279 |  | 0.529 |
| - male | 268 | 4.48 | 257 | 3.11 |  |  |  |  |
| - female | 222 | 4.95 | 221 | 7.69 |  |  |  |  |
| Child age |  |  |  |  |  | 0.920 |  | 0.355 |
| - Age <12 mo | 49 | 6.12 | 47 | 8.51 |  |  |  |  |
| - Age ≥12 mo | 441 | 4.53 | 431 | 4.87 |  |  |  |  |
| Admission criteria |  |  |  |  |  | 0.134 |  | 0.115 |
| - MUAC only | 212 | 4.72 | 217 | 7.37 |  |  |  |  |
| - WHZ only | 121 | 1.65 | 99 | 3.03 |  |  |  |  |
| - Edema only | 36 | 0.00 | 26 | 3.85 |  |  |  |  |
| - MUAC and WHZ | 118 | 8.47 | 135 | 3.70 |  |  |  |  |
| - MUAC and Edema | 3 | 33.3 | 1 | 0.00 |  |  |  |  |
| Stunting at admission |  |  |  |  |  | 0.999 |  | 0.970 |
| - Yes | 276 | 5.43 | 275 | 5.45 |  |  |  |  |
| - No | 214 | 3.74 | 203 | 4.92 |  |  |  |  |
| Health centre distance |  |  |  |  |  | 0.392 |  | 0.363 |
| - < 30 minutes | 284 | 4.23 | 293 | 5.80 |  |  |  |  |
| - ≥ 30 minutes | 206 | 5.34 | 185 | 4.32 |  |  |  |  |
| Edema at any visit |  |  |  |  |  | 0.861 |  | 0.674 |
| - Absence of Edema | 451 | 4.88 | 451 | 5.32 |  |  |  |  |
| - Presence of Edema | 39 | 2.56 | 27 | 3.70 |  |  |  |  |
| Morbidity |  |  |  |  |  | 0.480 |  | 0.758 |
| - Yes | 400 | 4.25 | 387 | 5.17 |  |  |  |  |
| - No | 90 | 6.67 | 91 | 5.49 |  |  |  |  |

*MUAC=mid-upper-arm circumference. RUTF=ready-to-use therapeutic food. WHZ=weight for-height Z. SD = Standard deviation. CI = Confidence interval*

## **Table I. Subgroup analysis of relapse (percentage)**

| Variables | Reduced dose | | Standard dose | | Unadjusted difference (95% CI) | p | Adjusted difference (95% CI) | p |
| --- | --- | --- | --- | --- | --- | --- | --- | --- |
|  | n | Percentage | n | Percentage |  |  |  |  |
| Child sex |  |  |  |  |  | 0.780 |  | 0.915 |
| - male | 111 | 3.60 | 122 | 3.28 |  |  |  |  |
| - female | 134 | 2.24 | 128 | 1.56 |  |  |  |  |
| Child age |  |  |  |  |  | 0.889 |  | 0.735 |
| - Age <12 mo | 21 | 4.76 | 20 | 5.00 |  |  |  |  |
| - Age ≥12 mo | 224 | 2.68 | 230 | 2.17 |  |  |  |  |
| Admission criteria |  |  |  |  |  | 0.911 |  | 0.952 |
| - MUAC only | 125 | 1.00 | 129 | 0.00 |  |  |  |  |
| - WHZ only | 55 | 5.45 | 42 | 7.14 |  |  |  |  |
| - Edema only | 21 | 4.76 | 18 | 0.00 |  |  |  |  |
| - MUAC and WHZ | 42 | 4.76 | 61 | 4.92 |  |  |  |  |
| - MUAC and Edema | 2 | 0.00 | 0 | 0.00 |  |  |  |  |
| Stunting at admission |  |  |  |  |  | 0.445 |  | 0.522 |
| - Yes | 147 | 1.36 | 152 | 1.97 |  |  |  |  |
| - No | 98 | 5.10 | 98 | 3.06 |  |  |  |  |
| Health centre distance |  |  |  |  |  | 0.566 |  | 0.129 |
| - < 30 minutes | 145 | 2.07 | 159 | 2.52 |  |  |  |  |
| - ≥ 30 minutes | 100 | 4.00 | 91 | 2.20 |  |  |  |  |
| Morbidity |  |  |  |  |  |  |  |  |
| - Yes | 31 | 0.00 | 44 | 2.27 |  |  |  |  |
| - No | 214 | 3.27 | 206 | 2.42 |  |  |  |  |
| Misseds visits |  |  |  |  |  | 0.416 |  | 0.676 |
| - Yes | 27 | 3.70 | 34 | 5.88 |  |  |  |  |
| - No | 218 | 2.75 | 216 | 1.85 |  |  |  |  |

*MUAC=mid-upper-arm circumference. RUTF=ready-to-use therapeutic food. WHZ=weight for-height Z. SD = Standard deviation. CI = Confidence interval*

## **Table J. Subgroup analysis of serious adverse events and illnesses (percentage)**

| Variables | Reduced dose | | Standard dose | | Unadjusted difference (95% CI) | p | Adjusted difference (95% CI) | p |
| --- | --- | --- | --- | --- | --- | --- | --- | --- |
|  | n | Percentage | n | Percentage |  |  |  |  |
| Child sex |  |  |  |  |  | 0.670 |  | 0.897 |
| - male | 268 | 18.7 | 257 | 18.7 |  |  |  |  |
| - female | 222 | 18.0 | 221 | 19.5 |  |  |  |  |
| Child age |  |  |  |  |  | 0.341 |  | 0.446 |
| - Age <12 mo | 49 | 16.3 | 47 | 25.5 |  |  |  |  |
| - Age ≥12 mo | 441 | 18.6 | 431 | 18.3 |  |  |  |  |
| Admission criteria |  |  |  |  |  | 0.031 |  | 0.045 |
| - MUAC only | 212 | 18.9 | 217 | 28.1 | 0.50 (0.02, 0.67) | 0.040 | 0.48 (-0.02, 0.98) | 0.058 |
| - WHZ only | 121 | 17.4 | 99 | 10.1 | -0.87 (-1.80, 0.05) | 0.065 | - 0.84 (-1.80, 0.13) | 0.088 |
| - Edema only | 36 | 19.4 | 26 | 11.5 | -0.62 (-2.07, 0.84) | 0.408 | No observations |  |
| - MUAC and WHZ | 118 | 18.6 | 135 | 12.6 | - 0.42 (-1.17, 0.32) | 0.264 | -0.21 (-0.99, 0.58) | 0.605 |
| - MUAC and Edema | 3 | 0.00 | 1 | 0.00 |  |  |  |  |
| Edema at any visit |  |  |  |  |  | 0.419 |  | 0.286 |
| - Absence of Edema | 39 | 18.0 | 27 | 11.1 |  |  |  |  |
| - Presence of Edema | 451 | 19.5 | 451 | 18.4 |  |  |  |  |
| Admission months |  |  |  |  |  | 0.031 |  | 0.053 |
| - Dry season | 219 | 20.1 | 212 | 26.9 | -0.40 (-0.89, 0.10) | 0.118 | 0.49 (-1.03, 0.04) | 0.070 |
| - Wet season | 271 | 17.0 | 266 | 12.8 | 0.36 (-0.13, 0.85) | 0.151 | 0.22 (-0.31, 0.75) | 0.420 |
| Missed visits |  |  |  |  |  | 0.746 |  | 0.758 |
| - Yes | 70 | 21.4 | 71 | 25.4 |  |  |  |  |
| - No | 420 | 18.9 | 407 | 17.9 |  |  |  |  |
| Household [1]Food security |  |  |  |  |  | 0.341 |  | 0.542 |
| - Food security | 10 | 2.00 | 6 | 3.33 |  |  |  |  |
| - Mildly food insecure | 3 | 0.00 | 8 | 12.5 |  |  |  |  |
| - Moderately or severely food insecure | 477 | 18.4 | 464 | 18.9 |  |  |  |  |

*MUAC=mid-upper-arm circumference. RUTF=ready-to-use therapeutic food. WHZ=weight for-height Z. SD = Standard deviation. CI = Confidence interval*

## **Table K. Subgroup analysis of duration edema melting**

| Variables | Reduced dose | | Standard dose | | Unadjusted difference (95% CI) | p | Adjusted difference (95% CI) | p |
| --- | --- | --- | --- | --- | --- | --- | --- | --- |
|  | n | Percentage | n | Percentage |  |  |  |  |
| Child sex |  |  |  |  |  | 0.526 |  | No observation |
| - male | 19 | 11.5 ± 7.83 | 15 | 8.87 ± 7.70 |  |  |  |  |
| - female | 20 | 11.6 ± 9.05 | 11 | 9.55 ± 5.66 |  |  |  |  |
| Child age |  |  |  |  |  | 0.098 |  | 0.230 |
| - Age <12 mo | 1 | 0.00 | 1 | 7.00 ± 0.00 | - Colinearity with dosage |  |  |  |
| - Age ≥12 mo | 38 | 11.8 ± 8.26 | 25 | 9.24 ± 6.92 | - 2.03 (-1.39, 5.46) | 0.244 |  |  |
| Morbidity |  |  |  |  |  | 0.241 |  | 0.225 |
| - Yes | 32 | 10.8 ± 7.36 | 24 | 8.46 ± 5.83 |  |  |  |  |
| - No | 7 | 14.9 ± 12.1 | 2 | 17.5 ± 14.8 |  |  |  |  |
| Missed visits |  |  |  |  |  | 0.807 |  | 0.468 |
| - Yes | 33 | 12.1 ± 8.73 | 22 | 9.55 ± 7.02 |  |  |  |  |
| - No | 6 | 8.17 ± 5.27 | 4 | 7.00 ± 5.72 |  |  |  |  |
| Admission months |  |  |  |  |  | 0.536 |  | 0.468 |
| - Dry season | 15 | 14.5 ± 9.19 | 11 | 10.8 ± 8.49 |  |  |  |  |
| - Wet season | 24 | 9.67 ± 7.42 | 15 | 7.93 ± 5.20 |  |  |  |  |

*MUAC=mid-upper-arm circumference. RUTF=ready-to-use therapeutic food. WHZ=weight for-height Z. SD = Standard deviation. CI = Confidence interval*

## **Table L. Subgroup analysis of MUAC gain velocity (mm/week)**

| Variables | Reduced dose | | Standard dose | | Unadjusted difference (95% CI) | p | Adjusted difference (95% CI) | p |
| --- | --- | --- | --- | --- | --- | --- | --- | --- |
|  | n | Mean ± SD | n | Mean ± SD |  |  |  |  |
| Child age |  |  |  |  |  | 0.053 |  | 0.469 |
| - Age <12 mo | 49 | 2.40 ± 1.28 | 47 | 2.18 ± 0.89 | 0.21 (-0.22, 0.63) | 0.345 |  |  |
| - Age ≥12 mo | 441 | 2.14 ± 0.96 | 431 | 2.34 ± 0.95 | - -0.18 (-0.29, - 0.07) | 0.001 |  |  |
| Child sex |  |  |  |  |  | 0.117 |  | 0.217 |
| - male | 268 | 2.09 ± 0.99 | 257 | 2.35 ± 0.93 |  |  |  |  |
| - female | 222 | 2.25 ± 0.99 | 221 | 2.28 ± 0.96 |  |  |  |  |
| Admission criteria |  |  |  |  |  | 0.150 |  | 0.198 |
| - MUAC only | 212 | 2.44 ± 0.92 | 217 | 2.40 ± 0.94 |  |  |  |  |
| - WHZ only | 121 | 1.87 ± 0.96 | 99 | 2.13 ± 0.96 |  |  |  |  |
| - Edema only | 36 | 1.04 ± 0.94 | 26 | 1.45 ± 1.09 |  |  |  |  |
| - MUAC and WHZ | 118 | 2.30 ± 0.88 | 135 | 2.49 ± 0.81 |  |  |  |  |
| - MUAC and Edema | 3 | 2.72 ± 0.82 | 1 | 2.14 |  |  |  |  |
| Edema at any visit |  |  |  |  |  | 0.696 |  | 0.528 |
| - Absence of Edema | 451 | 2.25 ± 0.95 | 451 | 2.37 ± 0.92 |  |  |  |  |
| - Presence of Edema | 39 | 1.17 ± 1.03 | 27 | 1.48 ± 1.07 |  |  |  |  |
| Morbidity |  |  |  |  |  | 0.757 |  | 0.959 |
| - Yes | 86 | 1.88 ± 0.71 | 89 | 2.07 ± 0.93 |  |  |  |  |
| - No | 404 | 2.23 ± 1.04 | 389 | 2.38 ± 0.94 |  |  |  |  |
| Admission months |  |  |  |  |  | 0.024 |  | 0.015 |
| - Dry season | 219 | 2.08 ± 1.14 | 212 | 2.40 ± 1.06 | -0.29 (-0.48, -0.10) | 0.002 | -0.25 (-0.43, -0.07) | 0.007 |
| - Wet season | 271 | 2.24 ± 0.86 | 266 | 2.26 ± 0.85 | -0.37 (-0.16, 0.09) | 0.571 | 0.02 (-0.09, 0.13) | 0.745 |
| Missed visits |  |  |  |  |  | 0.289 |  | 0.068 |
| - Yes | 70 | 1.84 ± 0.82 | 71 | 2.14 ± 0.92 |  |  | -0.06 (-0.17, 0.06) | 0.334 |
| - No | 420 | 2.22 ± 1.01 | 407 | 2.35 ± 0.95 |  |  | -0.28 (-0.54, -0.02) | 0.035 |

*MUAC=mid-upper-arm circumference. RUTF=ready-to-use therapeutic food. WHZ=weight for-height Z. SD = Standard deviation. CI = Confidence interval*
